# Diverse Synthesis of Fused Polyheterocyclic Compounds via [3 + 2] Cycloaddition of In Situ-Generated Heteroaromatic *N*-Ylides and Electron-Deficient Olefins

**DOI:** 10.3390/molecules28114410

**Published:** 2023-05-29

**Authors:** Zhen-Hua Wang, Tong Zhang, Li-Wen Shen, Xiu Yang, Yan-Ping Zhang, Yong You, Jian-Qiang Zhao, Wei-Cheng Yuan

**Affiliations:** 1 Innovation Research Center of Chiral Drugs, Institute for Advanced Study, Chengdu University, Chengdu 610106, China; 15680279213@163.com (T.Z.); yangxiucdu@163.com (X.Y.); zhangyanping@cdu.edu.cn (Y.-P.Z.); youyong@cdu.edu.cn (Y.Y.); zhaojianqiang@cdu.edu.cn (J.-Q.Z.); 2 College of Chemistry and Chemical Engineering, Zunyi Normal University, Zunyi 563006, China; shenlw1994@163.com

**Keywords:** [3 + 2] cycloaddition, diverse synthesis, heteroaromatic *N*-ylides, electron-deficient olefins, fused polyheterocycles

## Abstract

[3 + 2] Cycloaddition reactions of heteroaromatic *N*-ylides with electron-deficient olefins have been developed. The heteroaromatic *N*-ylides, in situ generated from *N*-phenacylbenzothiazolium bromides, can smoothly react with maleimides under very mild conditions, affording fused polycyclic octahydropyrrolo[3,4-*c*]pyrroles in good-to-excellent isolated yields. This reaction concept could also be extended to 3-trifluoroethylidene oxindoles and benzylidenemalononitriles as electron-deficient olefins for accessing highly functionalized polyheterocyclic compounds. A gram-scale experiment was also carried out to verify the practicability of the methodology.

## 1. Introduction

**Figure 1:**
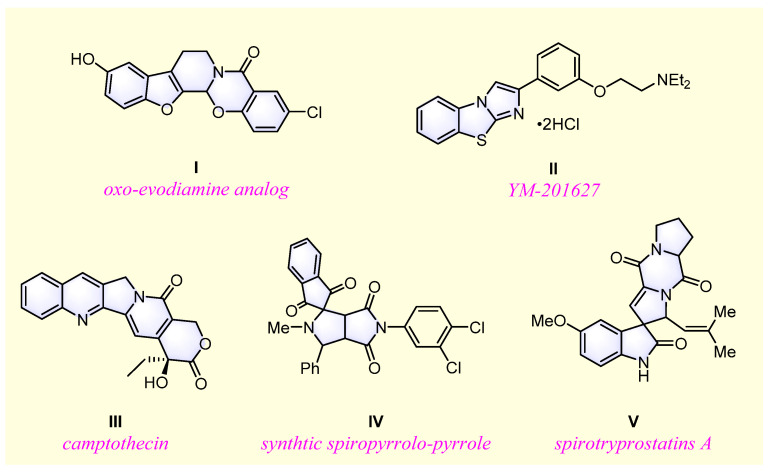
Representative biologically active molecules containing fused polyheterocyclic skeleton.

Polyheterocyclic skeletons are frequently found as the common backbone in a variety of natural alkaloids and synthetic organic molecules with remarkable biological activities [1,2,3,4]. As shown in Figure 1, the oxo-evodiamine analogue (**I**), YM-201627 (**II**), and camptothecin (**III**) have been verified to be tumor inhibitors. Spirocyclic furan analog (**IV**) has been identified as a potent inhibitor of bacterial phenylalanyl-*t*RNA synthetase. Spirotryprostatin A (**V**) has been isolated as a novel cell cycle inhibitor in mammals. Therefore, the construction of highly functionalized polyheterocyclic compounds continues to be an important area of research in modern organic synthetic chemistry. A large number of efficient synthetic tactics have been developed for the synthesis of various functionalized polyheterocycles [5,6,7,8,9,10]. Among the various reported methods, [3 + 2] cycloaddition has become one of the most powerful and straightforward synthetic approaches that can be used to construct polycyclic compounds [11,12,13,14,15,16].

**Scheme 1:**
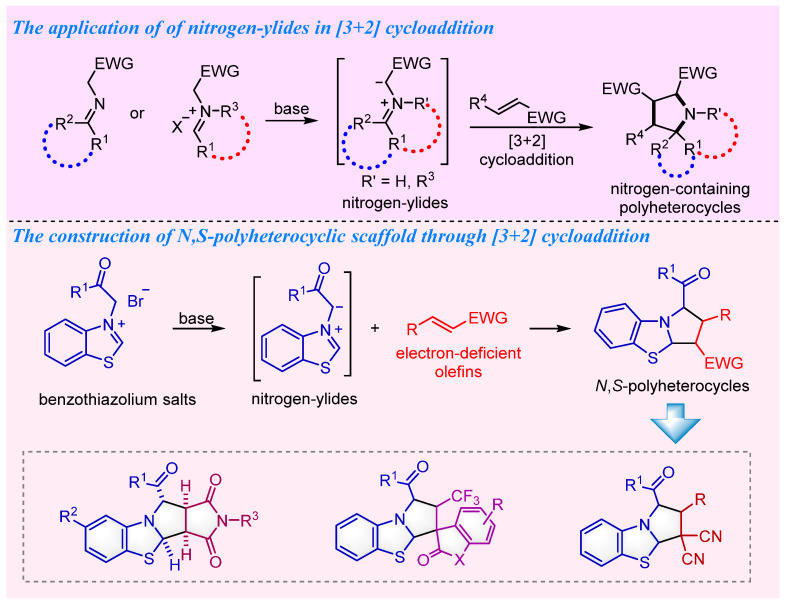
Profile of [3 + 2] cycloaddition of nitrogen-ylides in situ generated from imines or heteroarenium salts and the strategy for the diverse synthesis of structurally diverse fused polyheterocyclic compounds in this study.

Nitrogen-ylides, in situ generated from imines or heteroarenium salts, have proven to be versatile and valuable three-atom 4π-component synthons for accessing diverse nitrogen-containing heterocyclic compounds (Scheme 1, top) [17,18,19,20,21,22]. Various types of [3 + 2] cycloadditions involving imines have been sufficiently reported [23,24,25,26,27,28,29,30,31,32]. In contrast, the study of the use of heteroarenium salts for [3 + 2] cycloadditions remains relatively underdeveloped, which may be due to the extra stability of the aromaticity of aromatic heterocyclic ring. Thus far, heteroarenium salts derived from quinoline, isoquinoline, pyridine, and benzothiazole have been successfully used in various annulation processes to access diverse nitrogen-containing polyheterocycles [33,34,35,36,37,38,39,40,41,42,43,44]. In particular, benzothiazolium salts serving as heteroaromatic *N*-ylide precursors are extremely valuable for the simple and efficient synthesis of *N*,*S*-polyheterocyclic derivatives which are frequently found in natural products and pharmaceuticals [45,46,47,48]. However, a literature search revealed that, although many types of electron-deficient olefins acting as 2π-component have been successfully applied in [3 + 2] cycloaddition with various three-atom 4π-component partners [11,12,13,14,15,16,49,50], the related [3 + 2] cycloaddition reaction concerning the heteroaromatic *N*-ylides, in situ generated from benzothiazolium salts, with diverse electron-deficient olefins is very limited [51,52,53,54,55,56]. In this context, given the importance of *N*,S-polyheterocyclic scaffolds in medicinal and natural product chemistry and the great potential of the benzothiazolium salts for cycloaddition, expanding the application of benzothiazolium salts in the reaction with different type of electron-deficient olefins for the [3 + 2] cycloaddition to access structurally diverse polyheterocyclic compounds is highly desired. Therefore, based on our unremitting efforts in developing new synthetic methods for the construction of structurally diverse heterocyclic compounds [57,58,59,60,61], the [3 + 2] cycloaddition reactions of benzothiazolium salts and diverse electron-deficient olefins including maleimides, 3-trifluoroethylidene oxindoles, and benzylidenemalononitriles have been realized, furnishing varieties of functionalized fused polyheterocyclic compounds (Scheme 1, bottom). Herein, we hope to report the results of our study.

## 2. Results and Discussion

Initially, optimization of the reaction conditions was conducted by choosing benzothiazolium salt **1a** and *N*-phenylmaleimide **2a** as the model substrates (Table 1). Several inorganic bases were first tested in DCM at room temperature, and the use of Na_2_CO_3_ gave product **3aa** in a relatively high yield (entry 3 vs. entries 1, 2, and 4). Interestingly, triethylamine furnished trace product (entry 5). Upon further solvent examination (entries 6–9), the toluene was found to be an appropriate medium to yield the corresponding cycloaddition product with a 99% yield (entry 6). As a result, the best reaction condition consisted of a 1.5 equivalent of Na_2_CO_3_ and toluene as the solvent at room temperature.

**Scheme 2:**
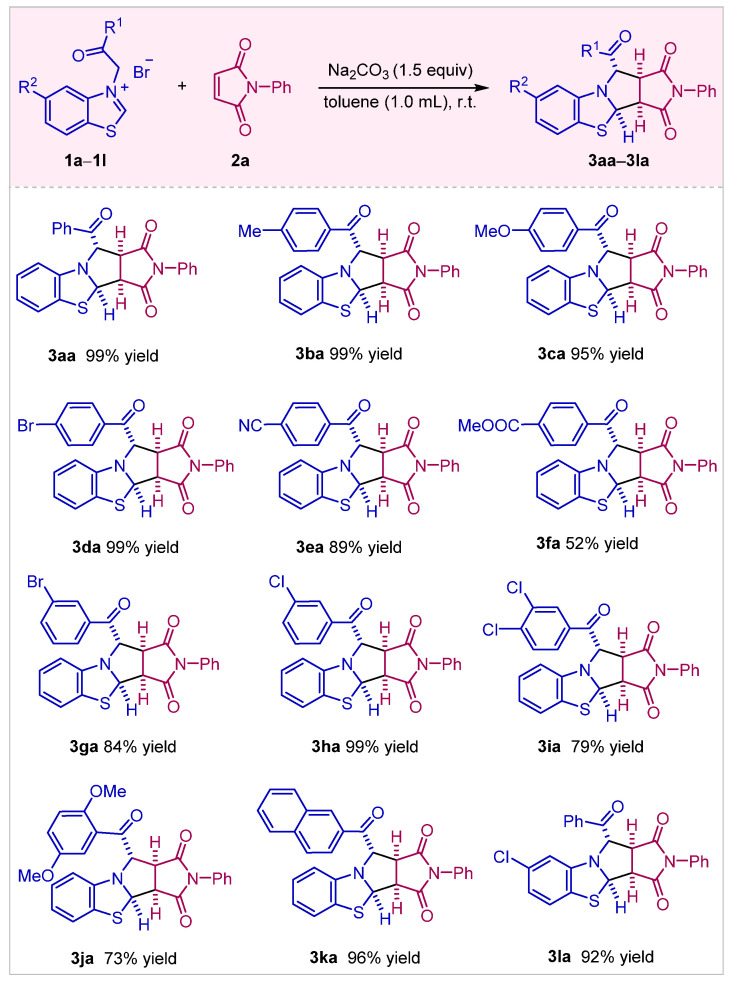
The substrate scope of benzothiazolium salts **1**. Reaction conditions: The reaction was carried out with **1a**–**1l** (0.15 mmol), **2a** (0.10 mmol), and Na_2_CO_3_ (0.15 mmol) in toluene (1.0 mL) at room temperature for 3 h. The yields refer to the isolated yield of product.

To verify the scalability of the developed [3 + 2] cycloaddition, the optimal reaction conditions were applied to other benzothiazolium salts (Scheme 2). The electronic nature and size of the substituents on benzoyl group showed little influence on the reactivity of the reaction, and the desired products **3ba**–**3ha** could be isolated in good to excellent yields (52–99%). Benzothiazolium salts **1i** and **1j** with two substituents on the benzene ring delivered good yields for **3ia** (79%) and **3ja** (73%). As for the introduction of naphthyl to benzothiazolium salt, the reaction also proceeded smoothly to provide the corresponding octahydropyrrolo[3,4-*c*]pyrrole **3ka** with a 96% yield. A smooth conversion of the substrate **1l**-bearing chlorine substituent on benzothiazole was observed in the reaction with **2a** to give product **3la** in a 92% yield.

**Scheme 3:**
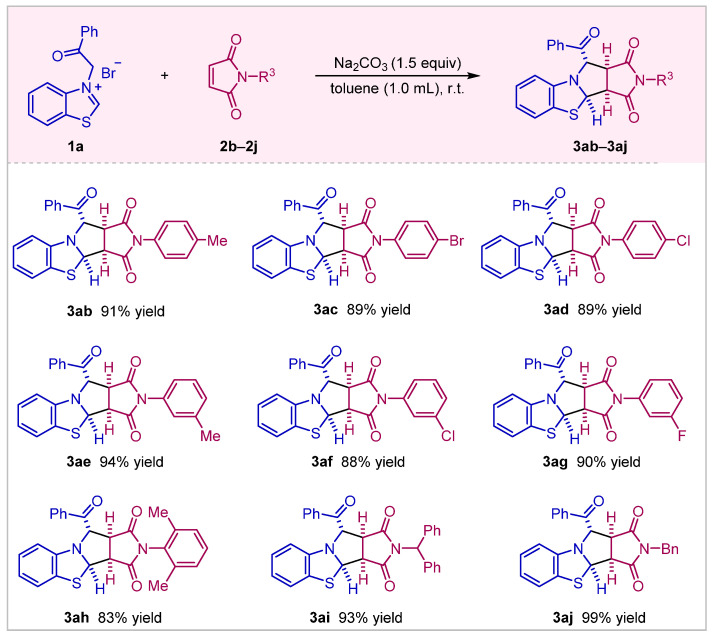
The substrate scope for maleimides **2**. Reaction conditions: The reaction was carried out with **1a** (0.15 mmol), **2b**–**2j** (0.10 mmol), and Na_2_CO_3_ (0.15 mmol) in toluene (1.0 mL) at room temperature for 3 h.

Following this, the reaction scope with respect to maleimides **2** was investigated (Scheme 3). The electronic properties of the substituent on the *para*-position of the phenyl ring had almost no effect on the developed transformation, leading to products **3ab**–**3ad** in excellent isolated yields (89–91%). Similarly, *meta*-substituted maleimides **2e**–**2g** were well tolerated in [3 + 2] cycloaddition and smoothly switched into octahydropyrrolo[3,4-*c*]pyrroles **3ae**–**3ag** with 88–94% yields. The reaction involving maleimide **2h** also performed very well, and a good yield was produced. Moreover, *N*-alkyl substituted maleimides **2i** and **2j** were compatible with the current system, resulting in 93% and 99% yields, respectively.

**Scheme 4:**
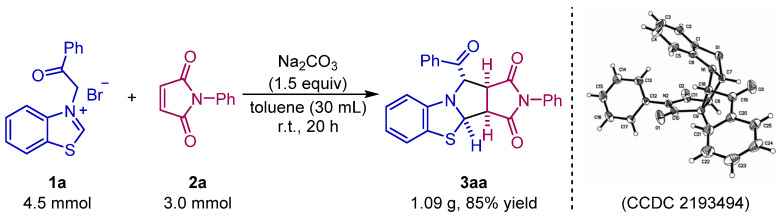
Scale-up experiment.

The synthetic practicability of the developed [3 + 2] cycloaddition was demonstrated by the scale-up experiment of benzothiazolium salt **1a** and *N-*phenylmaleimide **2a** under the standard conditions, and the product octahydropyrrolo[3,4-*c*]pyrrole **3aa** was isolated with a 85% yield (Scheme 4). In addition, the structure of **3aa** was unambiguously determined using single crystal X-ray diffraction analysis (Scheme 4, CCDC 2193494 (**3aa**) contains the supplementary crystallographic data for this paper. For details, see the Supporting Information).

**Scheme 5:**
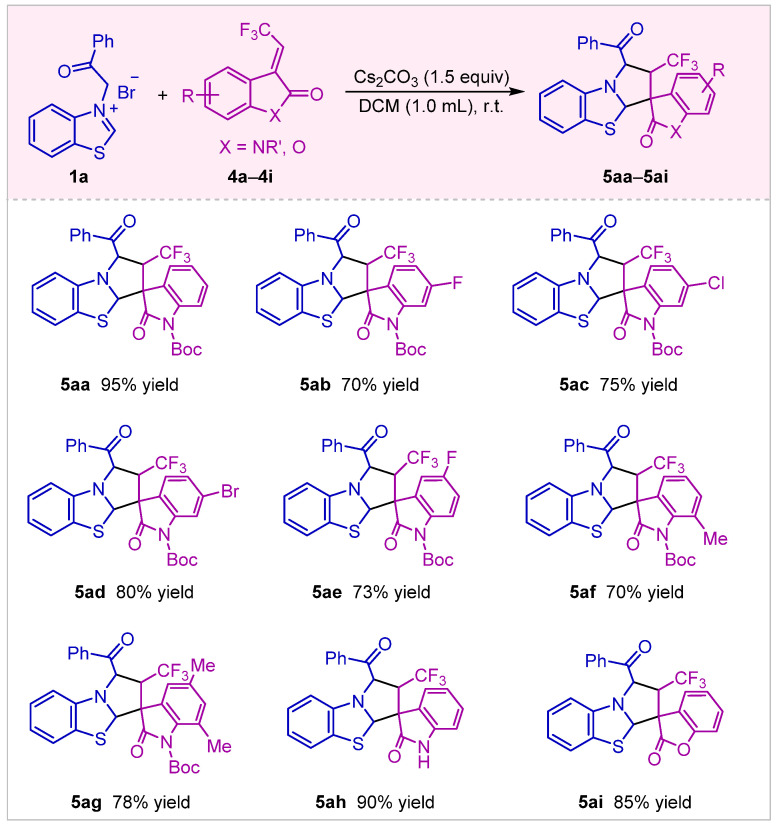
The [3 + 2] cycloaddition reaction of benzothiazolium salt with 3-trifluoroethylidene oxindoles. Reaction conditions: The reaction was carried out with **1a** (0.15 mmol), **4a**–**4i** (0.10 mmol), and Cs_2_CO_3_ (0.15 mmol) in DCM (1.0 mL) at room temperature for 18 h.

Owing to the unique properties of the trifluoromethyl unit in promoting the metabolic stability and bioavailability of many bioactive compounds [62,63], several CF_3_-containing heterocyclic compounds, in particular of trifluoromethyl-substituted pyrrolidines, have been synthesized with [3 + 2] cycloaddition reaction in the past few years [64,65,66,67,68]. To expand the application of benzothiazolium salts in constructing spiro-pyrrolidines, the [3 + 2] cycloaddition between benzothiazolium salts and 3-trifluoroethylidene oxindoles was conducted. After a careful screening of bases and solvents (For the detail of the procedure, see Supporting Information), the reaction of benzothiazolium salt **1a** with 3-trifluoroethylidene oxindole **4a** proceeded smoothly to produce the CF_3_-containing tetrahydrobenzo[*d*]pyrrolo[2,1-*b*]thiazole **5aa** with a 95% isolated yield (Scheme 5). On this basis, other 3-trifluoroethylidene oxindoles **4b**–**4h** were evaluated under the optimal reaction conditions, these substrates could be smoothly converted into the corresponding cycloadducts **5ab**–**5ah** in good yields (70–90%). Moreover, 3-trifluoroethylidene benzofuranone was also compatible with the developed system to give product **5ai** with a 85% yield.

**Scheme 6:**
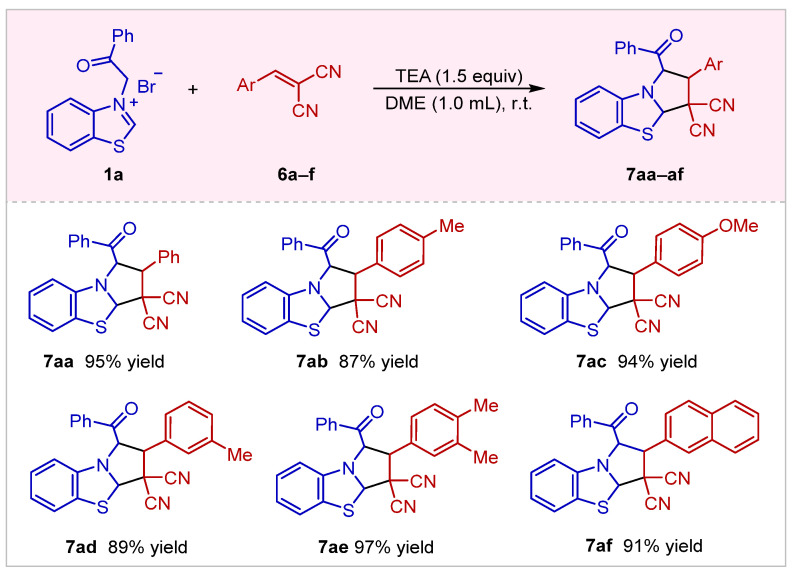
The [3 + 2] cycloaddition reaction of benzothiazolium salt with various benzylidenemalononitriles. Reaction conditions: The reaction was carried out with **1a** (0.15 mmol), **6a**–**6f** (0.10 mmol), and TEA (0.15 mmol) in 1,2-dimethoxyethane (DME, 1.0 mL) at room temperature for 3 h.

Encouraged by the above success, we questioned whether the application of benzothiazolium salt can be further expanded. Subsequently, many benzylidenemalononitriles **6** were surveyed via reaction with **1a**. The reaction optimization study demonstrated that triethylamine (TEA) was the best base for promoting the transformation (For the detail of the procedure, see Supporting Information). The reaction between **1a** and **6a** could furnish the desired product **7aa** in 95% yield (Scheme 6). Further studies indicated that the substituents on the aryl ring (Ar) of benzylidenemalononitriles have a limited effect on the reactivity of the reaction. The benzylidenemalononitriles **6b**–**6f** were compatible with the reaction condition to give the corresponding products **7ab**–**7af** in 87–97% yields.

## 3. Materials and Methods

### 3.1. General Information

Reagents were purchased from commercial sources and were used as received unless mentioned otherwise. Reactions were monitored with TLC. The NMR spectra were recorded with a Bruker Avance NEO 400 or 300. ^1^H NMR and ^13^C NMR spectra were recorded in CDCl_3_ or DMSO-*d*_6_. ^1^H NMR chemical shifts are reported in ppm relative to tetramethylsilane (TMS) with the solvent resonance employed as the internal standard (CDCl_3_ at 7.26 ppm, DMSO-*d*_6_ at 2.50 ppm). Data are reported as follows: chemical shift, multiplicity (s = singlet, br s = broad singlet, d = doublet, t = triplet, q = quartet, m = multiplet), coupling constants (Hz), and integration. ^13^C NMR chemical shifts are reported in ppm from tetramethylsilane (TMS) with the solvent resonance as the internal standard (CDCl_3_ at 77.16 ppm, DMSO-*d*_6_ at 39.52 ppm). Melting points products were recorded on a Büchi Melting Point B-545. The HRMS was recorded with an Agilent 6545 LC/Q-TOF mass spectrometer.

### 3.2. General Experimental Procedures for the Synthesis of Compounds ***3*** (Scheme 2 and Scheme 3)

In an ordinary vial charged with a magnetic stirring bar, *N*-phenacylbenzothiazolium bromides **1** (0.15 mmol, 1.5 equiv), maleimides **2** (0.1 mmol, 1.0 equiv), Na_2_CO_3_ (0.15 mmol, 1.5 equiv), and toluene (1.0 mL) were successively added. Then, the mixture was stirred at room temperature for the indicated time. Products **3** were isolated using flash chromatography on silica gel.

*10-Benzoyl-2-phenyl-3a,3b,10,10a-tetrahydro-1H-benzo[d]pyrrolo[3′,4′:3,4]pyrrolo[2,1-b]thiazole-1,3(2H)-dione* (**3aa**): Eluent: petroleum ether/EtOAc = 4:1, white solid (42.2 mg, 99% yield); R*_f_* = 0.50 (petroleum ether/EtOAc = 3:1); m.p. 190.2–192.4 °C; ^1^H NMR (400 MHz, DMSO-*d*_6_) *δ* 8.18 (d, *J =* 7.4 Hz, 2H, Ar-H), 7.71 (dd, *J =* 7.2 Hz, 1H, Ar-H), 7.64–7.54 (m, 2H, Ar-H), 7.36–7.28 (m, 4H, Ar-H), 7.21 (d, *J =* 7.6 Hz, 1H, Ar-H), 7.08 (dd, *J =* 7.7 Hz, 1H, Ar-H), 6.87 (dd, *J =* 7.5 Hz, 1H, Ar-H), 6.46 (dd, *J =* 6.7, 3.0 Hz, 2H, Ar-H), 6.31 (s, 1H, N-CH), 5.47 (d, *J =* 8.3 Hz, 1H, S-CH-N), 4.13 (d, *J =* 7.8 Hz, 1H, CH), 3.66 (t, *J =* 8.1 Hz, 1H, CH); ^13^C NMR (101 MHz, DMSO-*d*_6_) *δ* 195.2 (C=O), 176.3 (C=O), 173.4 (C=O), 146.5, 133.9, 133.8, 132.0, 129.1, 128.8, 128.7, 128.5, 126.7, 126.2, 125.2, 122.6, 121.8, 110.4, 71.3 (N-C), 68.3 (S-C-N), 50.3 (CH), 47.9 (CH); IR (neat) *ν* 3068, 2968, 1773, 1696, 1683, 1595, 1577, 1499, 1461, 1448, 1389, 1320, 1292, 1222, 1204, 1176, 1163, 991, 747, 688 cm^−1^; HRMS (ESI) *m*/*z*: [M + H]^+^ calcd for C_25_H_19_N_2_O_3_S 427.1111; found 427.1119.
*10-(4-Methylbenzoyl)-2-phenyl-3a,3b,10,10a-tetrahydro-1H-benzo[d]pyrrolo[3′,4′:3,4]pyrrolo[2,1-b]thiazole-1,3(2H)-dione* (**3ba**): Eluent: petroleum ether/EtOAc = 4:1, white solid (43.6 mg, 99% yield); R*_f_* = 0.54 (petroleum ether/EtOAc = 3:1); m.p. 195.4–197.3 °C; ^1^H NMR (400 MHz, DMSO-*d*_6_) *δ* 8.07 (d, *J =* 8.1 Hz, 2H, Ar-H), 7.40 (d, *J =* 8.0 Hz, 2H, Ar-H), 7.34–7.30 (m, 3H, Ar-H), 7.29 (d, *J =* 8.2 Hz, 1H, Ar-H), 7.20 (d, *J =* 7.6 Hz, 1H, Ar-H), 7.07 (dd, *J =* 7.7 Hz, 1H, Ar-H), 6.87 (dd, *J =* 7.5 Hz, 1H, Ar-H), 6.45 (dd, *J =* 6.4, 3.1 Hz, 2H, Ar-H), 6.25 (s, 1H, N-CH), 5.46 (d, *J =* 8.3 Hz, 1H, S-CH-N), 4.09 (d, *J =* 7.8 Hz, 1H, CH), 3.64 (t, *J =* 8.1 Hz, 1H, CH), 2.40 (s, 3H, CH_3_); **^1^**^3^C NMR (101 MHz, DMSO-*d*_6_) *δ* 194.7 (C=O), 176.4 (C=O), 173.4 (C=O), 146.6, 144.6, 132.1, 131.4, 129.4, 129.3, 128.8, 128.5, 126.7, 126.2, 125.2, 122.6, 121.8, 110.4, 71.4 (N-C), 68.2 (S-C-N), 50.3 (CH), 48.0 (CH), 21.3 (CH_3_); IR (neat) *ν* 3063, 3025, 2940, 1776, 1708, 1677, 1603, 1578, 1495, 1468, 1453, 1387, 1256, 1224, 1207, 1176, 1165, 1024, 990, 803, 751, 687 cm^−1^; HRMS (ESI) *m*/*z*: [M + H]^+^ calcd for C_26_H_21_N_2_O_3_S 441.1267; found 441.1275.
*10-(4-Methoxybenzoyl)-2-phenyl-3a,3b,10,10a-tetrahydro-1H-benzo[d]pyrrolo[3′,4′:3,4]pyrrolo[2,1-b]thiazole-1,3(2H)-dione* (**3ca**): Eluent: petroleum ether/EtOAc = 4:1, white solid (43.4 mg, 95% yield); R*_f_* = 0.35 (petroleum ether/EtOAc = 3:1); m.p. 189.0–189.7 °C; ^1^H NMR (400 MHz, DMSO-*d*_6_) *δ* 8.16 (d, *J =* 8.7 Hz, 2H, Ar-H), 7.37–7.27 (m, 4H, Ar-H), 7.21 (d, *J =* 7.6 Hz, 1H, Ar-H), 7.12 (d, *J =* 8.7 Hz, 2H, Ar-H), 7.07 (dd, *J =* 7.6 Hz, 1H, Ar-H), 6.87 (dd, *J =* 7.5 Hz, 1H, Ar-H), 6.47–6.43 (m, 2H, Ar-H), 6.24 (s, 1H, N-CH), 5.46 (d, *J =* 8.3 Hz, 1H, S-CH-N), 4.10 (d, *J =* 7.8 Hz, 1H, CH), 3.86 (s, 3H, OCH_3_), 3.64 (t, *J =* 8.1 Hz, 1H, CH); ^13^C NMR (101 MHz, DMSO-*d*_6_) *δ* 193.4 (C=O), 176.4 (C=O), 173.4 (C=O), 163.7, 146.6, 132.1, 131.6, 128.7, 128.5, 126.7, 126.5, 126.1, 125.2, 122.6, 121.8, 114.1, 110.4, 71.4 (N-C), 68.0 (S-C), 55.7 (OCH_3_), 50.3 (CH), 48.0 (CH); IR (neat) *ν* 3060, 2917, 2847, 1777, 1707, 1675, 1596, 1574, 1495, 1469, 1388, 1320, 1254, 1226, 1207, 1173, 1164, 1022, 819, 752, 688 cm^−1^; HRMS (ESI) *m*/*z*: [M + H]^+^ calcd for C_26_H_21_N_2_O_4_S 457.1217; found 457.1227.
*10-(4-Bromobenzoyl)-2-phenyl-3a,3b,10,10a-tetrahydro-1H-benzo[d]pyrrolo[3′,4′:3,4]pyrrolo[2,1-b]thiazole-1,3(2H)-dione* (**3da**): Eluent: petroleum ether/EtOAc = 4:1, white solid (50.0 mg, 99% yield); R*_f_* = 0.57 (petroleum ether/EtOAc = 3:1); m.p. 177.5–179.2 °C; ^1^H NMR (400 MHz, DMSO-*d*_6_) *δ* 8.07 (d, *J =* 8.4 Hz, 2H, Ar-H), 7.82 (d, *J =* 8.3 Hz, 2H, Ar-H), 7.37–7.26 (m, 4H, Ar-H), 7.21 (d, *J =* 7.6 Hz, 1H, Ar-H), 7.08 (dd, *J =* 7.7 Hz, 1H, Ar-H), 6.87 (dd, *J =* 7.5 Hz, 1H, Ar-H), 6.44 (dd, *J =* 6.5, 3.1 Hz, 2H, Ar-H), 6.28 (s, 1H, N-CH), 5.43 (d, *J =* 8.4 Hz, 1H, S-CH-N), 4.13 (d, *J =* 7.8 Hz, 1H, CH), 3.63 (t, *J =* 8.1 Hz, 1H, CH); ^13^C NMR (101 MHz, DMSO-*d*_6_) *δ* 194.6 (C=O), 176.3 (C=O), 173.3 (C=O), 146.4, 133.1, 132.0, 131.8, 131.0, 128.7, 128.5, 128.0, 126.7, 126.2, 125.2, 122.6, 121.9, 110.4, 71.3 (N-C), 68.4 (S-C-N), 50.3 (CH), 47.7 (CH); IR (neat) *ν* 3064, 2925, 2849, 1777, 1707, 1680, 1581, 1498, 1467, 1452, 1391, 1256, 1220, 1210, 1180, 1168, 1068, 1026, 1006, 859, 806, 755, 687 cm^−1^; HRMS (ESI) *m*/*z*: [M + H]^+^ calcd for C_25_H_18_BrN_2_O_3_S 505.0216, 507.0198; found 505.0218, 507.0203.
*4-(1,3-Dioxo-2-phenyl-2,3,3a,3b,10,10a-hexahydro-1H-benzo[d]pyrrolo[3′,4′:3,4]pyrrolo[2,1-b]thiazole-10-carbonyl)benzonitrile* (**3ea**): Eluent: petroleum ether/EtOAc = 4:1, white solid (40.2 mg, 89% yield); R*_f_* = 0.24 (petroleum ether/EtOAc = 3:1); m.p. 182.1–184.3 °C; ^1^H NMR (400 MHz, DMSO-*d*_6_) *δ* 8.26 (d, *J =* 8.2 Hz, 2H, Ar-H), 8.08 (d, *J =* 8.2 Hz, 2H, Ar-H), 7.38–7.27 (m, 4H, Ar-H), 7.22 (d, *J =* 7.5 Hz, 1H, Ar-H), 7.09 (dd, *J =* 7.6 Hz, 1H, Ar-H), 6.88 (dd, *J =* 7.5 Hz, 1H, Ar-H), 6.50–6.41 (m, 2H, Ar-H), 6.35 (s, 1H, N-CH), 5.42 (d, *J =* 8.4 Hz, 1H, S-CH-N), 4.17 (d, *J =* 7.8 Hz, 1H, CH), 3.64 (t, *J =* 8.1 Hz, 1H, CH); ^13^C NMR (101 MHz, DMSO-*d*_6_) *δ* 195.0 (C=O), 176.2 (C=O), 173.3 (C=O), 146.4, 137.7, 132.7, 132.0, 129.6, 128.7, 128.5, 126.7, 126.2, 125.2, 122.7, 122.0, 118.1, 115.5, 110.5, 71.2 (N-C), 68.7 (S-C-N), 50.3 (CH), 47.5 (CH); IR (neat) *ν* 3060, 2964, 2920, 2850, 2229, 1777, 1703, 1678, 1579, 1499, 1466, 1448, 1387, 1195, 1177, 1171, 1009, 752, 742, 686 cm^−1^; HRMS (ESI) *m*/*z*: [M + H]^+^ calcd for C_26_H_18_N_3_O_3_S 452.1063; found 452.1070.
*Methyl 4-(1,3-dioxo-2-phenyl-2,3,3a,3b,10,10a-hexahydro-1H-benzo[d]pyrrolo[3′,4′:3,4]pyrrolo[2,1-b]thiazole-10-carbonyl) benzoate* (**3fa**): Eluent: petroleum ether/EtOAc = 4:1, white solid (25.2 mg, 52% yield); R*_f_* = 0.31 (petroleum ether/EtOAc = 3:1); m.p. 183.9–186.2 °C; ^1^H NMR (400 MHz, DMSO-*d*_6_) *δ* 8.26 (d, *J =* 8.5 Hz, 2H, Ar-H), 8.13 (d, *J =* 8.4 Hz, 2H, Ar-H), 7.36–7.26 (m, 4H, Ar-H), 7.21 (d, *J =* 8.2 Hz, 1H, Ar-H), 7.08 (dd, *J =* 7.3 Hz, 1H, Ar-H), 6.88 (dd, *J =* 7.5 Hz, 1H, Ar-H), 6.50–6.40 (m, 2H, Ar-H), 6.32 (s, 1H, N-CH), 5.44 (d, *J =* 8.4 Hz, 1H, S-CH-N), 4.14 (d, *J =* 7.9 Hz, 1H, CH), 3.89 (s, 3H, CH_3_), 3.65 (t, *J =* 8.1 Hz, 1H, CH); ^13^C NMR (101 MHz, DMSO-*d*_6_) *δ* 195.1 (C=O), 176.2 (C=O), 173.3 (C=O), 165.5 (C=O), 146.4, 137.7, 133.6, 132.0, 129.4, 129.3, 128.7, 128.5, 126.7, 126.2, 125.2, 122.6, 122.0, 110.5, 71.2 (N-C), 68.6 (S-C-N), 52.6 (CH_3_), 50.3 (CH), 47.7 (CH); IR (neat) *ν* 3058, 2953, 2920, 2850, 1777, 1726, 1706, 1685, 1575, 1496, 1467, 1388, 1283, 1258, 1223, 1179, 1169, 1106, 1014, 868, 755, 707, 687 cm^−1^; HRMS (ESI) *m*/*z*: [M + H]^+^ calcd for C_27_H_21_N_2_O_5_S 485.1166; found 485.1171.
*10-(3-Bromobenzoyl)-2-phenyl-3a,3b,10,10a-tetrahydro-1H-benzo[d]pyrrolo[3′,4′:3,4]pyrrolo[2,1-b]thiazole-1,3(2H)-dione* (**3ga**): Eluent: petroleum ether/EtOAc = 4:1, white solid (42.4 mg, 84% yield); R*_f_* = 0.48 (petroleum ether/EtOAc = 3:1); m.p. 177.5–179.2 °C; ^1^H NMR (400 MHz, DMSO-*d*_6_) *δ* 8.28 (s, 1H, Ar-H), 8.15 (d, *J =* 7.9 Hz, 1H, Ar-H), 7.90 (d, *J =* 9.0 Hz, 1H, Ar-H), 7.56 (dd, *J =* 7.9 Hz, 1H, Ar-H), 7.37–7.28 (m, 4H, Ar-H), 7.21 (d, *J =* 8.2 Hz, 1H, Ar-H), 7.09 (dd, *J =* 8.1 Hz, 1H, Ar-H), 6.88 (dd, *J =* 7.5 Hz, 1H, Ar-H), 6.51–6.39 (m, 2H, Ar-H), 6.31 (s, 1H, N-CH), 5.46 (d, *J =* 8.4 Hz, 1H, S-CH-N), 4.13 (d, *J =* 7.9 Hz, 1H, CH), 3.63 (t, *J =* 8.1 Hz, 1H, CH); ^13^C NMR (101 MHz, DMSO-*d*_6_) *δ* 194.3 (C=O), 176.2 (C=O), 173.3 (C=O), 146.5, 136.3, 136.1, 132.0, 131.7, 131.0, 128.7, 128.5, 128.0, 126.7, 126.2, 125.1, 122.6, 122.0, 121.9, 110.4, 71.3 (N-C), 68.6 (S-C-N), 50.2 (CH), 47.8 (CH); IR (neat) *ν* 3061, 2963, 2923, 2850, 1779, 1709, 1698, 1681, 1566, 1499, 1464, 1450, 1390, 1369, 1220, 1210, 1182, 1164, 1135, 1030, 843, 767, 744, 694 cm^−1^; HRMS (ESI) *m*/*z*: [M + H]^+^ calcd for C_25_H_18_BrN_2_O_3_S 505.0216, 507.0198; found 505.0218, 507.0206.
*10-(3-Chlorobenzoyl)-2-phenyl-3a,3b,10,10a-tetrahydro-1H-benzo[d]pyrrolo[3′,4′:3,4]pyrrolo[2,1-b]thiazole-1,3(2H)-dione* (**3ha**): Eluent: petroleum ether/EtOAc = 4:1, white solid (45.6 mg, 99% yield); R*_f_* = 0.51 (petroleum ether/EtOAc = 3:1); m.p. 185.7–186.2 °C; ^1^H NMR (400 MHz, DMSO-*d*_6_) *δ* 8.14 (s, 1H, Ar-H), 8.11 (d, *J* = 7.9 Hz, 1H, Ar-H), 7.77 (d, *J* = 9.2 Hz, 1H, Ar-H), 7.63 (dd, *J* = 7.9 Hz, 1H, Ar-H), 7.39–7.26 (m, 4H, Ar-H), 7.21 (d, *J* = 7.5 Hz, 1H, Ar-H), 7.09 (dd, *J* = 7.7 Hz, 1H, Ar-H), 6.88 (dd, *J* = 7.5 Hz, 1H, Ar-H), 6.53–6.37 (m, 2H, Ar-H), 6.31 (s, 1H, N-CH), 5.46 (d, *J* = 8.4 Hz, 1H, S-CH-N), 4.13 (d, *J* = 7.9 Hz, 1H, CH), 3.63 (t, *J* = 8.1 Hz, 1H, CH); ^13^C NMR (101 MHz, DMSO-*d*_6_) *δ* 194.4 (C=O), 176.2 (C=O), 173.3 (C=O), 146.5, 135.9, 133.6, 133.5, 132.0, 130.8, 128.8, 128.7, 128.5, 127.7, 126.7, 126.2, 125.1, 122.6, 121.9, 110.4, 71.3 (N-C), 68.6 (S-C-N), 50.2 (CH), 47.8 (CH); IR (neat) *ν* 3064, 2977, 2920, 2850, 1780, 1708, 1678, 1579, 1498, 1466, 1450, 1387, 1253, 1222, 1196, 1180, 1162, 1028, 841, 753, 742, 685 cm^−1^; HRMS (ESI) *m*/*z*: [M + H]^+^ calcd for C_25_H_18_ClN_2_O_3_S 461.0721, 463.0700; found 461.0728, 463.0706.
*10-(3,4-Dichlorobenzoyl)-2-phenyl-3a,3b,10,10a-tetrahydro-1H-benzo[d]pyrrolo[3′,4′:3,4]pyrrolo[2,1-b]thiazole-1,3(2H)-dione* (**3ia**): Eluent: petroleum ether/EtOAc = 4:1, white solid (39.1 mg, 79% yield); R*_f_* = 0.51 (petroleum ether/EtOAc = 3:1); m.p. 212.4–213.7 °C; ^1^H NMR (400 MHz, DMSO-*d*_6_) *δ* 8.31 (d, *J* = 2.0 Hz, 1H, Ar-H), 8.08 (dd, *J* = 8.4, 2.0 Hz, 1H, Ar-H), 7.88 (d, *J* = 8.4 Hz, 1H, Ar-H), 7.40–7.28 (m, 4H, Ar-H), 7.22 (d, *J* = 7.5 Hz, 1H, Ar-H), 7.09 (dd, *J* = 7.3 Hz, 1H, Ar-H), 6.88 (dd, *J* = 7.4 Hz, 1H, Ar-H), 6.53–6.39 (m, 2H, Ar-H), 6.31 (s, 1H, N-CH), 5.45 (d, *J* = 8.4 Hz, 1H, S-CH-N), 4.14 (d, *J* = 7.9 Hz, 1H, CH), 3.63 (t, *J* = 8.1 Hz, 1H, CH); ^13^C NMR (101 MHz, DMSO-*d*_6_) *δ* 193.7 (C=O), 176.2 (C=O), 173.3 (C=O), 146.4, 136.6, 134.3, 132.0, 131.7, 131.1, 131.0, 129.1, 128.8, 128.6, 126.7, 126.2, 125.2, 122.7, 122.0, 110.4, 71.3 (N-C), 68.6 (S-C-N), 50.2 (CH), 47.7 (CH); IR (neat) *ν* 3098, 3063, 2973, 2963, 1778, 1708, 1685, 1578, 1498, 1469, 1450, 1387, 1344, 1264, 1220, 1169, 1027, 969, 807, 801, 756, 714, 686, 671 cm^−1^; HRMS (ESI) *m*/*z*: [M + H]^+^ calcd for C_25_H_17_Cl_2_N_2_O_3_S 495.0331, 497.0307, 499.0288; found 495.0336, 497.0312, 499.0295.
*10-(2,5-Dimethoxybenzoyl)-2-phenyl-3a,3b,10,10a-tetrahydro-1H-benzo[d]pyrrolo[3′,4′:3,4]pyrrolo[2,1-b]thiazole-1,3(2H)-dione* (**3ja**): Eluent: petroleum ether/EtOAc = 4:1, white solid (35.5 mg, 73% yield); R*_f_* = 0.29 (petroleum ether/EtOAc = 3:1); m.p. 197.5–199.2 °C; ^1^H NMR (400 MHz, DMSO-*d*_6_) *δ* 7.35–7.29 (m, 3H, Ar-H), 7.23 (d, *J* = 2.8 Hz, 1H, Ar-H), 7.20–7.12 (m, 3H, Ar-H), 7.05 (dd, *J* = 7.3 Hz, 1H, Ar-H), 6.89 (d, *J* = 7.9 Hz, 1H, Ar-H), 6.83 (dd, *J* = 7.5 Hz, 1H, Ar-H), 6.54–6.39 (m, 2H, Ar-H), 6.04 (s, 1H, N-CH), 5.49 (d, *J* = 8.4 Hz, 1H, S-CH-N), 4.10 (d, *J* = 7.8 Hz, 1H, CH), 3.91 (s, 3H, CH_3_), 3.76 (s, 3H, CH_3_), 3.63 (t, *J* = 8.1 Hz, 1H, CH); ^13^C NMR (101 MHz, DMSO-*d*_6_) *δ* 198.1 (C=O), 176.2 (C=O), 173.4 (C=O), 153.0, 152.3, 146.7, 132.1, 128.7, 128.5, 126.7, 126.1, 125.7, 125.1, 122.4, 121.6, 119.9, 114.5, 113.5, 110.3, 71.6 (N-C), 71.3 (S-C-N), 56.3 (OCH_3_), 55.6 (OCH_3_), 50.4 (CH), 47.7 (CH); IR (neat) *ν* 3060, 3011, 2948, 2923, 2830, 1779, 1711, 1675, 1577, 1494, 1458, 1416, 1378, 1222, 1188, 1173, 1013, 836, 811, 743, 731, 690, 622 cm^−1^; HRMS (ESI) *m*/*z*: [M + H]^+^ calcd for C_27_H_23_N_2_O_5_S 487.1322; found 487.1330.
*10-(2-Naphthoyl)-2-phenyl-3a,3b,10,10a-tetrahydro-1H-benzo[d]pyrrolo[3′,4′:3,4]pyrrolo[2,1-b]thiazole-1,3(2H)-dione* (**3ka**): Eluent: petroleum ether/EtOAc = 4:1, white solid (45.8 mg, 96% yield); R*_f_* = 0.51 (petroleum ether/EtOAc = 3:1); m.p. 201.3–203.2 °C; ^1^H NMR (400 MHz, DMSO-*d*_6_) *δ* 8.90 (s, 1H, Ar-H), 8.15 (dd, *J* = 8.6, 1.4 Hz, 1H, Ar-H), 8.10 (dd, *J* = 9.4 Hz, 2H, Ar-H), 8.03 (d, *J* = 8.1 Hz, 1H, Ar-H), 7.71 (dd, *J* = 7.0 Hz, 1H, Ar-H), 7.65 (dd, *J* = 7.0 Hz, 1H, Ar-H), 7.43 (d, *J* = 8.0 Hz, 1H, Ar-H), 7.37–7.30 (m, 3H, Ar-H), 7.22 (d, *J* = 7.1 Hz, 1H, Ar-H), 7.11 (dd, *J* = 7.7 Hz, 1H, Ar-H), 6.88 (dd, *J* = 7.5 Hz, 1H, Ar-H), 6.51–6.46 (m, 2H, Ar-H), 6.45 (s, 1H, N-CH), 5.53 (d, *J* = 8.3 Hz, 1H, S-CH-N), 4.17 (d, *J* = 7.9 Hz, 1H, CH), 3.69 (t, *J* = 8.1 Hz, 1H, CH); ^13^C NMR (101 MHz, DMSO-*d*_6_) *δ* 195.1 (C=O), 176.4 (C=O), 173.4 (C=O), 146.6, 135.3, 132.1, 131.9, 131.3, 131.1, 129.7, 129.1, 128.7, 128.5, 128.4, 127.8, 127.2, 126.7, 126.2, 125.2, 124.4, 122.6, 121.8, 110.5, 71.4 (N-C), 68.3 (S-C-N), 50.4 (CH), 48.1 (CH); IR (neat) *ν* 3059, 2919, 2849, 1781, 1708, 1680, 1497, 1462, 1381, 1274, 1179, 1154, 1124, 1024, 1002, 815, 746, 690 cm^−1^; HRMS (ESI) *m*/*z*: [M + H]^+^ calcd for C_29_H_21_N_2_O_3_S 477.1267; found 477.1276.
*10-Benzoyl-7-chloro-2-phenyl-3a,3b,10,10a-tetrahydro-1H-benzo[d]pyrrolo[3′,4′:3,4]pyrrolo[2,1-b]thiazole-1,3(2H)-dione* (**3la**): Eluent: petroleum ether/EtOAc = 4:1, white solid (42.4 mg, 92% yield); R*_f_* = 0.15 (petroleum ether/EtOAc = 3:1); m.p. 175.1–177.1 °C; ^1^H NMR (400 MHz, DMSO-*d*_6_) *δ* 8.19 (d, *J* = 7.4 Hz, 2H, Ar-H), 7.71 (dd, *J* = 7.4 Hz, 1H, Ar-H), 7.61 (dd, *J* = 7.6 Hz, 2H, Ar-H), 7.48 (d, *J* = 1.9 Hz, 1H, Ar-H), 7.37 (dd, *J* = 5.5, 1.9 Hz, 3H, Ar-H), 7.21 (d, *J* = 8.2 Hz, 1H, Ar-H), 6.87 (dd, *J* = 8.2, 1.9 Hz, 1H, Ar-H), 6.64–6.52 (m, 2H, Ar-H), 6.37 (s, 1H, N-CH), 5.59 (d, *J* = 8.3 Hz, 1H, S-CH-N), 4.10 (d, *J* = 7.8 Hz, 1H, CH), 3.67 (t, *J* = 8.1 Hz, 1H, CH); ^13^C NMR (101 MHz, DMSO-*d*_6_) *δ* 195.0 (C=O), 176.1 (C=O), 173.2 (C=O), 148.3, 133.9, 133.6, 132.0, 130.7, 129.2, 128.9, 128.8, 128.6, 126.4, 124.4, 123.4, 121.2, 110.3, 72.1 (N-C), 68.5 (S-C-N), 50.2 (CH), 48.3 (CH); IR (neat) *ν* 3078, 3058, 2963, 2817, 1788, 1722, 1706, 1682, 1595, 1578, 1498, 1458, 1448, 1381, 1251, 1227, 1196, 1166, 1153, 1020, 989, 867, 806, 752, 698, 691, 682 cm^−1^; HRMS (ESI) *m*/*z*: [M + H]^+^ calcd for C_25_H_18_ClN_2_O_3_S 461.0721, 463.0700; found 461.0722, 463.0701.
*10-Benzoyl-2-(p-tolyl)-3a,3b,10,10a-tetrahydro-1H-benzo[d]pyrrolo[3′,4′:3,4]pyrrolo[2,1-b]thiazole-1,3(2H)-dione* (**3ab**): Eluent: petroleum ether/EtOAc = 4:1, white solid (40.1 mg, 91% yield); R*_f_* = 0.15 (petroleum ether/EtOAc = 6:1); m.p. 185.7–187.5 °C; ^1^H NMR (400 MHz, DMSO-*d*_6_) *δ* 8.17 (d, *J* = 7.5 Hz, 2H, Ar-H), 7.70 (dd, *J* = 7.3 Hz, 1H, Ar-H), 7.59 (dd, *J* = 7.6 Hz, 2H, Ar-H), 7.30 (d, *J* = 8.0 Hz, 1H, Ar-H), 7.20 (d, *J* = 7.5 Hz, 1H, Ar-H), 7.12 (d, *J* = 8.1 Hz, 2H, Ar-H), 7.07 (dd, *J* = 7.6 Hz, 1H, Ar-H), 6.86 (dd, *J* = 7.5 Hz, 1H, Ar-H), 6.33 (d, *J* = 8.1 Hz, 2H, Ar-H), 6.29 (s, 1H, N-CH), 5.46 (d, *J* = 8.3 Hz, 1H, S-CH-N), 4.10 (d, *J* = 7.8 Hz, 1H, CH), 3.63 (t, *J* = 8.1 Hz, 1H, CH), 2.26 (s, 3H, CH_3_); ^13^C NMR (101 MHz, DMSO-*d*_6_) *δ* 195.2 (C=O), 176.4 (C=O), 173.4 (C=O), 146.5, 138.1, 133.9, 133.8, 129.4, 129.2, 129.1, 128.8, 126.4, 126.1, 125.2, 122.6, 121.8, 110.4, 71.3 (N-C), 68.3 (S-C-N), 50.2 (CH), 47.8 (CH), 20.7 (CH_3_); IR (neat) *ν* 2999, 2954, 2920, 2849, 1777, 1698, 1686, 1675, 1514, 1459, 1449, 1395, 1309, 1253, 1238, 1224, 1170, 1157, 847, 784, 703, 682, 640, 609 cm^−1^; HRMS (ESI) *m*/*z*: [M + H]^+^ calcd for C_26_H_21_N_2_O_3_S 441.1267; found 441.1273.
*10-Benzoyl-2-(4-bromophenyl)-3a,3b,10,10a-tetrahydro-1H-benzo[d]pyrrolo[3′,4′:3,4]pyrrolo[2,1-b]thiazole-1,3(2H)-dione* (**3ac**): Eluent: petroleum ether/EtOAc = 4:1, white solid (45.0 mg, 89% yield); R*_f_* = 0.15 (petroleum ether/EtOAc = 6:1); m.p. 180.4–183.7 °C; ^1^H NMR (400 MHz, DMSO-*d*_6_) *δ* 8.17 (d, *J* = 7.7 Hz, 2H, Ar-H), 7.70 (dd, *J* = 7.3 Hz, 1H, Ar-H), 7.59 (dd, *J* = 7.6 Hz, 2H, Ar-H), 7.55 (d, *J* = 8.4 Hz, 2H, Ar-H), 7.31 (d, *J* = 8.0 Hz, 1H, Ar-H), 7.20 (d, *J* = 7.6 Hz, 1H, Ar-H), 7.07 (dd, *J* = 7.7 Hz, 1H, Ar-H), 6.86 (dd, *J* = 7.5 Hz, 1H, Ar-H), 6.43 (d, *J* = 8.5 Hz, 2H, Ar-H), 6.31 (s, 1H, N-CH), 5.45 (d, *J* = 8.4 Hz, 1H, S-CH-N), 4.12 (d, *J* = 7.8 Hz, 1H, CH), 3.66 (t, *J* = 8.1 Hz, 1H, CH); ^13^C NMR (101 MHz, DMSO-*d*_6_) *δ* 195.1 (C=O), 176.1 (C=O), 173.1 (C=O), 146.5, 134.8, 133.9, 131.8, 131.2, 129.1, 128.8, 128.6, 126.2, 125.1, 122.6, 121.9, 121.6, 110.5, 71.4 (N-C), 68.3 (S-C-N), 50.4 (CH), 47.9 (CH); IR (neat) *ν* 3094, 3059, 2917, 2849, 1786, 1708, 1690, 1579, 1488, 1463, 1450, 1389, 1257, 1226, 1165, 1063, 1010, 845, 799, 738, 726, 705, 695, 634, 604 cm^−1^; HRMS (ESI) *m*/*z*: [M + H]^+^ calcd for C_25_H_18_BrN_2_O_3_S 505.0216, 507.0198; found 505.0217, 507.0203.
*10-Benzoyl-2-(4-chlorophenyl)-3a,3b,10,10a-tetrahydro-1H-benzo[d]pyrrolo[3′,4′:3,4]pyrrolo[2,1-b]thiazole-1,3(2H)-dione* (**3ad**): Eluent: petroleum ether/EtOAc = 4:1, white solid (41.0 mg, 89% yield); R*_f_* = 0.15 (petroleum ether/EtOAc = 6:1); m.p. 182.3–185.4 °C; ^1^H NMR (400 MHz, DMSO-*d*_6_) *δ* 8.18 (d, *J* = 8.1 Hz, 2H, Ar-H), 7.69 (dd, *J* = 7.3 Hz, 1H, Ar-H), 7.58 (dd, *J* = 7.6 Hz, 2H, Ar-H), 7.36 (d, *J* = 8.5 Hz, 2H, Ar-H), 7.27 (d, *J* = 7.8 Hz, 1H, Ar-H), 7.17 (d, *J* = 7.6 Hz, 1H, Ar-H), 7.06 (dd, *J* = 7.7 Hz, 1H, Ar-H), 6.86 (dd, *J* = 7.5 Hz, 1H, Ar-H), 6.50 (d, *J* = 8.8 Hz, 2H, Ar-H), 6.28 (s, 1H, N-CH), 5.47 (d, *J* = 8.2 Hz, 1H, S-CH-N), 4.13 (d, *J* = 7.9 Hz, 1H, CH), 3.68 (t, *J* = 8.1 Hz, 1H, CH); ^13^C NMR (101 MHz, DMSO-*d*_6_) *δ* 194.7 (C=O), 176.0 (C=O), 173.0 (C=O), 146.3, 133.8, 133.7, 133.2, 130.6, 129.0, 128.7, 128.6, 128.1, 126.0, 125.2, 122.5, 121.8, 110.3, 71.4 (N-C), 68.2 (S-C-N), 50.3 (CH), 47.9 (CH); IR (neat) *ν* 3060, 2984, 2919, 2850, 1787, 1709, 1690, 1579, 1491, 1465, 1450, 1390, 1260, 1227, 1168, 1083, 1016, 735, 700, 636, 605 cm^−1^; HRMS (ESI) *m*/*z*: [M + H]^+^ calcd for C_25_H_18_ClN_2_O_3_S 461.0721, 463.0700; found 461.0730, 463.0711.
*10-Benzoyl-2-(m-tolyl)-3a,3b,10,10a-tetrahydro-1H-benzo[d]pyrrolo[3′,4′:3,4]pyrrolo[2,1-b]thiazole-1,3(2H)-dione* (**3ae**): Eluent: petroleum ether/EtOAc = 4:1, white solid (41.4 mg, 94% yield); R*_f_* = 0.15 (petroleum ether/EtOAc = 6:1); m.p. 187.9–190.1 °C: ^1^H NMR (400 MHz, DMSO-*d*_6_) *δ* 8.17 (d, *J* = 7.3 Hz, 2H, Ar-H), 7.71 (dd, *J* = 7.4 Hz, 1H, Ar-H), 7.60 (dd, *J* = 7.6 Hz, 2H, Ar-H), 7.31 (d, *J* = 7.9 Hz, 1H, Ar-H), 7.21 (dd, *J* = 7.7 Hz, 2H, Ar-H), 7.13 (d, *J* = 7.6 Hz, 1H, Ar-H), 7.09 (dd, *J* = 8.2 Hz, 1H, Ar-H), 6.89 (dd, *J* = 7.3 Hz, 1H, Ar-H), 6.38 (d, *J* = 7.8 Hz, 1H, Ar-H), 6.30 (s, 1H, Ar-H), 6.01 (s, 1H, N-CH), 5.46 (d, *J* = 8.3 Hz, 1H, S-CH-N), 4.11 (d, *J* = 7.8 Hz, 1H, CH), 3.63 (t, *J* = 8.1 Hz, 1H, CH), 2.21 (s, 3H, CH_3_); ^13^C NMR (101 MHz, DMSO-*d*_6_) *δ* 195.1 (C=O), 176.4 (C=O), 173.4 (C=O), 146.6, 138.3, 133.9, 133.8, 132.0, 129.1, 128.8, 128.5, 127.1, 126.2, 125.2, 123.8, 122.6, 121.8, 110.4, 71.3 (N-C), 68.3 (S-C-N), 50.3 (CH), 47.8 (CH), 20.8 (CH_3_); IR (neat) *ν* 3059, 2963, 2919, 2850, 1785, 1708, 1685, 1596, 1579, 1490, 1460, 1446, 1383, 1256, 1231, 1224, 1186, 1168, 1130, 1028, 1016, 986, 884, 844, 768, 742, 704, 690, 630, 605, 735, 700, 636, 607 cm^−1^; HRMS (ESI) *m*/*z*: [M + H]^+^ calcd for C_26_H_21_N_2_O_3_S 441.1267; found 441.1275.
*10-Benzoyl-2-(3-chlorophenyl)-3a,3b,10,10a-tetrahydro-1H-benzo[d]pyrrolo[3′,4′:3,4]pyrrolo[2,1-b]thiazole-1,3(2H)-dione* (**3af**): Eluent: petroleum ether/EtOAc = 4:1, white solid (40.6 mg, 88% yield); R*_f_* = 0.15 (petroleum ether/EtOAc = 6:1); m.p. 213.4–214.7 °C; ^1^H NMR (400 MHz, DMSO-*d*_6_) *δ* 8.17 (d, *J* = 7.3 Hz, 2H, Ar-H), 7.70 (dd, *J* = 7.4 Hz, 1H, Ar-H), 7.59 (dd, *J* = 7.6 Hz, 2H, Ar-H), 7.45–7.36 (m, 2H, Ar-H), 7.34 (d, *J* = 8.0 Hz, 1H, Ar-H), 7.23 (d, *J* = 6.9 Hz, 1H, Ar-H), 7.10 (dd, *J* = 7.7 Hz, 1H, Ar-H), 6.88 (dd, *J* = 7.5 Hz, 1H, Ar-H), 6.57 (dt, *J* = 7.4, 1.7 Hz, 1H, Ar-H), 6.33 (s, 1H, N-CH), 6.32–6.31 (m, 1H, Ar-H), 5.45 (d, *J* = 8.3 Hz, 1H, S-CH-N), 4.13 (d, *J* = 7.8 Hz, 1H, CH), 3.67 (t, *J* = 8.1 Hz, 1H, CH); ^13^C NMR (101 MHz, DMSO-*d*_6_) *δ* 195.0 (C=O), 176.1 (C=O), 173.0 (C=O), 146.5, 133.9, 133.2, 132.9, 130.4, 129.1, 128.8, 128.6, 126.5, 126.2, 125.5, 125.2, 122.6, 122.0, 110.5, 71.4 (N-C), 68.3 (S-C-N), 50.4 (CH), 47.9 (CH); IR (neat) *ν* 3064, 2952, 2921, 2850, 1779, 1710, 1676, 1593, 1577, 1477, 1460, 1448, 1382, 1255, 1240, 1222, 1174, 1153, 979, 848, 783, 748, 740, 684, 678, 630 cm^−1^; HRMS (ESI) *m*/*z*: [M + H]^+^ calcd for C_25_H_18_ClN_2_O_3_S 461.0721, 463.0700; found 461.0729, 463.0705.
*10-Benzoyl-2-(3-fluorophenyl)-3a,3b,10,10a-tetrahydro-1H-benzo[d]pyrrolo[3′,4′:3,4]pyrrolo[2,1-b]thiazole-1,3(2H)-dione* (**3ag**): Eluent: petroleum ether/EtOAc = 4:1, white solid (40.0 mg, 90% yield); R*_f_* = 0.15 (petroleum ether/EtOAc = 6:1); m.p. 190.7–192.1 °C; ^1^H NMR (400 MHz, DMSO-*d*_6_) *δ* 8.18 (d, *J* = 7.2 Hz, 2H, Ar-H), 7.70 (dd, *J* = 7.4 Hz, 1H, Ar-H), 7.60 (dd, *J* = 7.6 Hz, 2H, Ar-H), 7.44–7.36 (m, 1H, Ar-H), 7.36–7.32 (m, 1H, Ar-H), 7.25–7.18 (m, 2H, Ar-H), 7.10 (dd, *J* = 7.2 Hz, 1H, Ar-H), 6.88 (dd, *J* = 7.5 Hz, 1H, Ar-H), 6.41 (d, *J* = 8.7 Hz, 1H, Ar-H), 6.33 (s, 1H, N-CH), 6.26–6.09 (m, 1H, Ar-H), 5.46 (d, *J* = 8.3 Hz, 1H, S-CH-N), 4.14 (d, *J* = 7.9 Hz, 1H, CH), 3.68 (dd, *J* = 8.1 Hz, 1H, CH); ^13^C NMR (101 MHz, DMSO-*d*_6_) *δ* 195.0 (C=O), 176.1 (C=O), 173.0 (C=O), 161.6 (d, *J* = 244.8 Hz, 1C), 146.5, 133.9, 133.4 (d, *J* = 10.2 Hz, 1C), 130.5 (d, *J* = 8.8 Hz, 1C), 129.1, 128.8, 126.2, 125.2, 122.9 (d, *J* = 3.0 Hz, 1C), 122.6, 121.9, 115.6 (d, *J* = 20.8 Hz, 1C), 113.8 (d, *J* = 23.6 Hz, 1C), 110.5, 71.4 (N-C), 68.3 (S-C-N), 50.4 (CH), 47.9 (CH); IR (neat) *ν* 3055, 2969, 2918, 1780, 1702, 1681, 1594, 1579, 1490, 1461, 1450, 1386, 1320, 1291, 1255, 1224, 1180, 1162, 1136, 995, 899, 787, 744, 721, 681, 606 cm^−1^; HRMS (ESI) *m*/*z*: [M + H]^+^ calcd for C_25_H_18_FN_2_O_3_S 445.1017; found 445.1024.
*10-Benzoyl-2-(2,6-dimethylphenyl)-3a,3b,10,10a-tetrahydro-1H-benzo[d]pyrrolo[3′,4′:3,4]pyrrolo[2,1-b]thiazole-1,3(2H)-dione* (**3ah**): Eluent: petroleum ether/EtOAc = 4:1, white solid (37.7 mg, 83% yield); R*_f_* = 0.23 (petroleum ether/EtOAc = 6:1); m.p. 201.3–203.1 °C; ^1^H NMR (400 MHz, DMSO-*d*_6_) *δ* 8.20 (d, *J* = 7.3 Hz, 2H, Ar-H), 7.71 (dd, *J* = 7.4 Hz, 1H, Ar-H), 7.64–7.56 (m, 2H, Ar-H), 7.22 (d, *J* = 8.0 Hz, 1H, Ar-H), 7.17 (d, *J* = 7.6 Hz, 1H, Ar-H), 7.11 (dd, *J* = 7.6 Hz, 2H, Ar-H), 7.06–6.95 (m, 2H, Ar-H), 6.78 (dd, *J* = 7.8 Hz, 1H, Ar-H), 6.21 (s, 1H, N-CH), 5.69 (d, *J* = 8.7 Hz, 1H, S-CH-N), 4.36 (d, *J* = 9.5 Hz, 1H, CH), 3.90 (t, *J* = 8.6 Hz, 1H, CH), 1.98 (s, 3H, CH_3_), 1.07 (s, 3H, CH_3_); ^13^C NMR (101 MHz, DMSO-*d*_6_) *δ* 195.3 (C=O), 176.1 (C=O), 172.8 (C=O), 145.8, 136.0, 135.6, 134.9, 133.9, 133.8, 130.4, 129.0, 128.8, 128.1, 126.0, 125.4, 122.4, 121.4, 109.9, 70.5 (N-C), 67.6 (S-C-N), 50.3 (CH), 47.7 (CH), 17.4 (CH_3_), 15.6 (CH_3_); IR (neat) *ν* 3060, 2965, 2920, 1780, 1706, 1684, 1596, 1577, 1466, 1446, 1368, 1232, 1192, 1173, 1028, 772, 740, 687 cm^−1^; HRMS (ESI) *m*/*z*: [M + H]^+^ calcd for C_27_H_23_N_2_O_3_S 455.1424; found 455.1427.
*2-Benzhydryl-10-benzoyl-3a,3b,10,10a-tetrahydro-1H-benzo[d]pyrrolo[3′,4′:3,4]pyrrolo[2,1-b]thiazole-1,3(2H)-dione* (**3ai**): Eluent: petroleum ether/EtOAc = 4:1, white solid (48.0 mg, 93% yield); R*_f_* = 0.33 (petroleum ether/EtOAc = 6:1); m.p. 204.3–205.7 °C; ^1^H NMR (400 MHz, DMSO-*d*_6_) *δ* 8.15 (d, *J* = 7.2 Hz, 2H, Ar-H), 7.69 (dd, *J* = 7.4 Hz, 1H, Ar-H), 7.63–7.53 (m, 2H, Ar-H), 7.26 (dd, *J* = 5.0, 1.8 Hz, 3H, Ar-H), 7.18 (d, *J* = 7.3 Hz, 1H, Ar-H), 7.14–7.07 (m, 4H, Ar-H), 7.07–7.02 (m, 1H, Ar-H), 6.95 (dd, *J* = 6.8, 2.9 Hz, 2H, Ar-H), 6.86 (dd, *J* = 7.8 Hz, 1H, Ar-H), 6.58 (d, *J* = 7.6 Hz, 2H, Ar-H), 6.16 (s, 1H, N-CH), 6.07 (s, 1H, N-CHPh_2_), 5.64 (d, *J* = 8.6 Hz, 1H, S-CH-N), 4.04 (d, *J* = 9.1 Hz, 1H, CH), 3.69 (t, *J* = 8.5 Hz, 1H, CH); ^13^C NMR (101 MHz, DMSO-*d*_6_) *δ* 195.2 (C=O), 176.6 (C=O), 173.7 (C=O), 145.7, 137.3, 137.2, 133.8, 133.7, 129.0, 128.8, 128.3, 128.2, 128.2, 127.7, 127.5, 127.0, 126.0, 124.8, 122.2, 121.4, 109.9, 70.7 (N-C), 67.9 (S-C-N), 58.0 (N-CHPh_2_), 50.0 (CH), 47.8 (CH); IR (neat) *ν* 3059, 2970, 2919, 1781, 1703, 1596, 1579, 1494, 1465, 1448, 1383, 1354, 1230, 1164, 1076, 1027, 743, 695, 605 cm^−1^; HRMS (ESI) *m*/*z*: [M + H]^+^ calcd for C_32_H_25_N_2_O_3_S 517.1580; found 517.1586.
*10-Benzoyl-2-benzyl-3a,3b,10,10a-tetrahydro-1H-benzo[d]pyrrolo[3′,4′:3,4]pyrrolo[2,1-b]thiazole-1,3(2H)-dione* (**3aj**): Eluent: petroleum ether/EtOAc = 4:1, white solid (43.6 mg, 99% yield); R*_f_* = 0.18 (petroleum ether/EtOAc = 6:1); m.p. 179.3–182.1 °C; ^1^H NMR (400 MHz, DMSO-*d*_6_) *δ* 8.17 (d, *J* = 7.3 Hz, 2H, Ar-H), 7.70 (t, *J* = 7.4 Hz, 1H, Ar-H), 7.59 (t, *J* = 7.6 Hz, 2H, Ar-H), 7.18–7.05 (m, 4H, Ar-H), 7.04 (d, *J* = 7.6 Hz, 1H, Ar-H), 6.99 (t, *J* = 7.2 Hz, 1H, Ar-H), 6.80 (t, *J* = 7.2 Hz, 1H, Ar-H), 6.65 (d, *J* = 7.0 Hz, 2H, Ar-H), 6.11 (s, 1H, N-CH), 5.54 (d, *J* = 8.5 Hz, 1H, S-CH-N), 4.38–4.16 (m, 2H, CH_2_), 4.02 (d, *J* = 8.2 Hz, 1H, CH), 3.65 (t, *J* = 8.3 Hz, 1H, CH); ^13^C NMR (101 MHz, DMSO-*d*_6_) *δ* 195.3 (C=O), 177.2 (C=O), 174.0 (C=O), 145.8, 135.2, 133.9, 133.8, 129.0, 128.8, 128.5, 126.9, 126.5, 125.9, 124.9, 122.2, 121.5, 109.8, 70.7 (N-C), 67.7 (S-C-N), 50.3 (CH), 47.8 (CH), 42.0 (CH_2_); IR (neat) *ν* 3061, 2970, 2922, 1781, 1700, 1595, 1579, 1465, 1448, 1428, 1395, 1340, 1227, 1164, 1154, 1026, 1001, 872, 741, 730, 694, 605 cm^−1^; HRMS (ESI) *m*/*z*: [M + H]^+^ calcd for C_26_H_21_N_2_O_3_S 441.1267; found 441.1275.

### 3.3. Experimental Procedures for the Scale-Up Experiment (Scheme 4)

To a solution of benzothiazolium salt **1a** (4.5 mmol, 1.5 equiv) and *N*-phenylmaleimide **2a** (3.0 mmol, 1.0 equiv) in toluene (30 mL) was added Na_2_CO_3_ (4.5 mmol, 1.5 equiv) successively. Then, the mixture was stirred at room temperature for 20 h. The product **3aa** (1.09 g, 85% yield) was isolated using flash chromatography on silica gel.

### 3.4. General Experimental Procedures for Synthesis of Compounds ***5*** (Scheme 5)

In an ordinary vial charged with a magnetic stirring bar, *N*-phenacylbenzothiazolium bromide **1a** (0.15 mmol, 1.5 equiv), **4** (0.1 mmol, 1.0 equiv), Na_2_CO_3_ (0.15 mmol, 1.5 equiv), and DCM (1.0 mL) were successively added. The mixture was stirred at room temperature for 20 h. Products **5** were isolated using flash chromatography on silica gel.

*Tert-butyl 1-benzoyl-2′-oxo-2-(trifluoromethyl)-1,2-dihydro-3aH-spiro[benzo[d]pyrrolo[2,1-b]thiazole-3,3′-indoline]-1′-carboxylate* (**5aa**): Eluent: petroleum ether/EtOAc = 40:1, white solid (53.8 mg, 95% yield); R*_f_* = 0.33 (petroleum ether/EtOAc = 20:1); m.p. 160.7–162.2 °C; ^1^H NMR (400 MHz, CDCl_3_) *δ* 8.16 (d, *J* = 7.0 Hz, 2H, Ar-H), 7.95 (d, *J* = 8.2 Hz, 1H, Ar-H), 7.71 (dd, *J* = 7.4 Hz, 1H, Ar-H), 7.65–7.54 (m, 2H, Ar-H), 7.31 (dd, *J* = 7.4 Hz, 1H, Ar-H), 7.02–6.91 (m, 1H, Ar-H), 6.88–6.81 (m, 1H, Ar-H), 6.80–6.72 (m, 2H, Ar-H), 6.47 (dd, *J* = 8.0 Hz, 2H, Ar-H), 6.05 (s, 1H, N-CH-S), 5.49 (d, *J* = 9.7 Hz, 1H, N-CH), 4.45–4.26 (m, 1H, CF_3_-CH), 1.66 (s, 9H, C(CH_3_)_3_); ^13^C NMR (101 MHz, CDCl_3_) *δ* 197.0 (C=O), 171.9 (C=O), 148.6, 147.1, 140.3, 135.4, 134.5, 129.7, 129.4, 128.7, 126.9, 126.1, 125.8, 124.5 (q, *J* = 278.9 Hz, 1C, CF_3_), 123.8, 122.7, 121.9, 121.8, 115.3, 108.9, 85.4 (C_Boc_), 81.0 (N-C-S), 66.2 (N-C), 62.2 (C-CONBoc), 54.7 (q, *J* = 28.5 Hz, 1C, CF_3_-C), 28.1 ((CH_3_)_3_); IR (neat) *ν* 3059, 2982, 2971, 2934, 1756, 1731, 1692, 1578, 1473, 1467, 1448, 1396, 1369, 1299, 1273, 1253, 1210, 1173, 1146, 1117, 1081, 972, 840, 759, 745, 699, 654 cm^−1^; HRMS (ESI) *m*/*z*: [M + H]^+^ calcd for C_30_H_26_F_3_N_2_O_4_S 567.1560; found 567.1565.
*Tert-butyl 1-benzoyl-6′-fluoro-2′-oxo-2-(trifluoromethyl)-1,2-dihydro-3aH-spiro[benzo[d]pyrrolo[2,1-b]thiazole-3,3′-indoline]-1′-carboxylate* (**5ab**): Eluent: petroleum ether/EtOAc = 40:1, white solid (40.9 mg, 70% yield); R*_f_* = 0.37 (petroleum ether/EtOAc = 20:1); m.p. 173.2–174.8 °C; ^1^H NMR (300 MHz, CDCl_3_) *δ* 8.14 (d, *J* = 7.3 Hz, 2H, Ar-H), 7.79–7.67 (m, 2H, Ar-H), 7.66–7.52 (m, 2H, Ar-H), 7.06–6.89 (m, 1H, Ar-H), 6.87–6.70 (m, 2H, Ar-H), 6.58–6.50 (m, 1H, Ar-H), 6.48 (d, *J* = 7.9 Hz, 1H, Ar-H), 6.41–6.26 (m, 1H, Ar-H), 5.98 (s, 1H, N-CH-S), 5.46 (d, *J* = 9.5 Hz, 1H, N-CH), 4.47–4.22 (m, 1H, CF_3_-CH), 1.66 (s, 9H, C(CH_3_)_3_); ^13^C NMR (75 MHz, CDCl_3_) *δ* 196.7 (C=O), 171.9 (C=O), 163.3 (d, *J* = 247.1 Hz, 1C), 148.4, 147.1, 141.7 (d, *J* = 12.5 Hz, 1C), 135.5, 134.5, 129.4, 128.8, 127.0 (d, *J* = 9.8 Hz, 1C), 126.8, 126.3, 124.6 (q, *J* = 278.9 Hz, 1C, CF_3_), 122.1, 122.0, 118.2 (d, *J* = 3.4 Hz, 1C), 110.7 (d, *J* = 22.7 Hz, 1C), 109.0, 104.2 (d, *J* = 29.8 Hz, 1C), 85.9 (C_Boc_), 81.0 (N-C-S), 66.1 (N-C), 62.0 (C-CONBoc), 54.5 (q, *J* = 28.5 Hz, 1C, CF_3_-C), 28.2 ((CH_3_)_3_); IR (neat) *ν* 3058, 2987, 2971, 2935, 1762, 1731, 1692, 1606, 1597, 1579, 1497, 1473, 1447, 1396, 1368, 1359, 1326, 1299, 1270, 1257, 1237, 1209, 1179, 1145, 1128, 1112, 1083, 1031, 889, 861, 848, 813, 755, 746 cm^−1^; HRMS (ESI) *m*/*z*: [M + H]^+^ calcd for C_30_H_25_F_4_N_2_O_4_S 585.1466; found 585.1474.
*Tert-butyl 1-benzoyl-6′-chloro-2′-oxo-2-(trifluoromethyl)-1,2-dihydro-3aH-spiro[benzo[d]pyrrolo[2,1-b]thiazole-3,3′-indoline]-1′-carboxylate* (**5ac**): Eluent: petroleum ether/EtOAc = 40:1, white solid (45.1 mg, 75% yield); R*_f_* = 0.37 (petroleum ether/EtOAc = 20:1); m.p. 165.4–167.0 °C; ^1^H NMR (300 MHz, CDCl_3_) *δ* 8.14 (d, *J* = 7.2 Hz, 2H, Ar-H), 8.02 (d, *J* = 2.0 Hz, 1H, Ar-H), 7.72 (dd, *J* = 7.4 Hz, 1H, Ar-H), 7.65–7.53 (m, 2H, Ar-H), 7.06–6.90 (m, 1H, Ar-H), 6.87–6.69 (m, 3H, Ar-H), 6.48 (d, *J* = 8.0 Hz, 1H, Ar-H), 6.31 (d, *J* = 8.2 Hz, 1H, Ar-H), 5.98 (s, 1H, N-CH-S), 5.46 (d, *J* = 9.5 Hz, 1H, N-CH), 4.48–4.21 (m, 1H, CF_3_-CH), 1.66 (s, 9H, C(CH_3_)_3_); ^13^C NMR (75 MHz, CDCl_3_) *δ* 196.7 (C=O), 171.6 (C=O), 148.4, 147.0, 141.4, 135.7, 135.5, 134.5, 129.4, 128.8, 126.7, 126.6, 126.3, 124.6 (d, *J* = 279.0 Hz, 1C, CF_3_), 123.9, 122.1, 122.0, 121.2, 116.2, 109.0, 85.9 (C_Boc_), 81.0 (N-C-S), 66.1 (N-C), 62.2 (C-CONBoc), 54.6 (q, *J* = 28.6 Hz, 1C, CF_3_-C), 28.2 ((CH_3_)_3_); IR (neat) *ν* 2981, 2936, 1764, 1735, 1687, 1604, 1596, 1581, 1483, 1469, 1448, 1426, 1396, 1367, 1354, 1321, 1275, 1238, 1210, 1182, 1165, 1142, 1125, 1086, 1081, 1011, 971, 747, 740 cm^−1^; HRMS (ESI) *m*/*z*: [M + H]^+^ calcd for C_30_H_25_ClF_3_N_2_O_4_S 601.1170, 603.1153; found 601.1178, 603.1164.
*Tert-butyl 1-benzoyl-6′-bromo-2′-oxo-2-(trifluoromethyl)-1,2-dihydro-3aH-spiro[benzo[d]pyrrolo[2,1-b]thiazole-3,3′-indoline]-1′-carboxylate* (**5ad**): Eluent: petroleum ether/EtOAc = 40:1, white solid (51.6 mg, 80% yield); R*_f_* = 0.37 (petroleum ether/EtOAc = 20:1); m.p. 179.7–181.6 °C; ^1^H NMR (300 MHz, CDCl_3_) *δ* 8.18 (d, *J* = 1.8 Hz, 1H, Ar-H), 8.14 (d, *J* = 7.1 Hz, 2H, Ar-H), 7.71 (dd, *J* = 7.3 Hz, 1H, Ar-H), 7.65–7.54 (m, 2H, Ar-H), 7.06–6.91 (m, 2H, Ar-H), 6.87–6.70 (m, 2H, Ar-H), 6.48 (d, *J* = 7.9 Hz, 1H, Ar-H), 6.25 (d, *J* = 8.2 Hz, 1H, Ar-H), 5.98 (s, 1H, N-CH-S), 5.46 (d, *J* = 9.4 Hz, 1H, N-CH), 4.49–4.20 (m, 1H, CF_3_-CH), 1.66 (s, 9H, C(CH_3_)_3_); ^13^C NMR (75 MHz, CDCl_3_) *δ* 196.6 (C=O), 171.5 (C=O), 148.4, 147.0, 141.5, 135.5, 134.5, 129.4, 128.7, 126.9, 126.8, 126.7, 126.3, 124.6 (q, *J* = 279.2 Hz, 1C, CF_3_), 123.6, 122.1, 122.0, 121.7, 118.9, 108.9, 85.9 (C_Boc_), 80.9 (N-C-S), 66.0 (N-C), 62.2 (C-CONBoc), 54.5 (q, *J* = 28.6 Hz, 1C, CF_3_-C), 28.1 ((CH_3_)_3_); IR (neat) *ν* 2981, 2935, 1764, 1733, 1686, 1598, 1580, 1468, 1448, 1396, 1367, 1353, 1319, 1274, 1237, 1209, 1181, 1165, 1142, 1126, 1089, 1029, 1010, 972, 871, 833, 798, 765, 748, 740, 653 cm^−1^; HRMS (ESI) *m*/*z*: [M + H]^+^ calcd for C_30_H_25_BrF_3_N_2_O_4_S 645.0665, 647.0649; found 645.0672, 647.0655.
*Tert-butyl 1-benzoyl-5′-fluoro-2′-oxo-2-(trifluoromethyl)-1,2-dihydro-3aH-spiro[benzo[d]pyrrolo[2,1-b]thiazole-3,3′-indoline]-1′-carboxylate* (**5ae**): Eluent: petroleum ether/EtOAc = 40:1, white solid (42.7 mg, 73% yield); R*_f_* = 0.37 (petroleum ether/EtOAc = 20:1); m.p. 150.7–152.5 °C; ^1^H NMR (300 MHz, CDCl_3_) *δ* 8.16 (d, *J* = 7.2 Hz, 2H, Ar-H), 7.94 (dd, *J* = 9.1, 4.7 Hz, 1H, Ar-H), 7.72 (dd, *J* = 7.4 Hz, 1H, Ar-H), 7.67–7.53 (m, 2H, Ar-H), 7.07–6.94 (m, 2H, Ar-H), 6.87–6.73 (m, 2H, Ar-H), 6.51 (d, *J* = 7.8 Hz, 1H, Ar-H), 6.11 (d, *J* = 6.9 Hz, 1H, Ar-H), 5.99 (s, 1H, N-CH-S), 5.49 (d, *J* = 9.4 Hz, 1H, N-CH), 4.53–4.15 (m, 1H, CF_3_-CH), 1.65 (s, 9H, C(CH_3_)_3_); ^13^C NMR (75 MHz, CDCl_3_) *δ* 196.6 (C=O), 171.5 (C=O), 159.1 (d, *J* = 243.6 Hz, 1C), 148.6, 146.9, 136.5 (d, *J* = 2.5 Hz, 1C), 135.5, 134.5, 129.4, 128.8, 126.5 (d, *J* = 16.4 Hz, 1C), 126.4, 124.6 (q, *J* = 278.9 Hz, 1C, CF_3_), 124.5 (d, *J* = 8.7 Hz, 1C), 122.2, 122.0, 116.6, 116.4 (d, *J* = 13.6 Hz, 1C), 113.5 (d, *J* = 26.1 Hz, 1C), 109.0, 85.6 (C_Boc_), 80.9 (N-C-S), 65.8 (N-C), 62.6 (C-CONBoc), 54.5 (q, *J* = 28.6 Hz, 1C, CF_3_-C), 28.2 ((CH_3_)_3_); IR (neat) *ν* 3058, 2983, 2936, 1798, 1761, 1741, 1735, 1690, 1607, 1594, 1579, 1481, 1471, 1450, 1396, 1371, 1343, 1324, 1295, 1272, 1242, 1212, 1172, 1145, 1125, 1084, 999, 819, 747, 732, 641 cm^−1^; HRMS (ESI) *m*/*z*: [M + H]^+^ calcd for C_30_H_25_F_4_N_2_O_4_S 585.1466; found 585.1470.
*Tert-butyl 1-benzoyl-7′-methyl-2′-oxo-2-(trifluoromethyl)-1,2-dihydro-3aH-spiro[benzo[d]pyrrolo[2,1-b]thiazole-3,3′-indoline]-1′-carboxylate* (**5af**): Eluent: petroleum ether/EtOAc = 40:1, white solid (40.6 mg, 70% yield); R*_f_* = 0.37 (petroleum ether/EtOAc = 20:1); m.p. 167.8–170.2 °C; ^1^H NMR (400 MHz, CDCl_3_) *δ* 8.16 (d, *J* = 7.8 Hz, 2H, Ar-H), 7.71 (dd, *J* = 7.3 Hz, 1H, Ar-H), 7.65–7.56 (m, 2H, Ar-H), 7.12 (d, *J* = 7.7 Hz, 1H, Ar-H), 6.95 (dd, *J* = 7.5 Hz, 1H, Ar-H), 6.83–6.69 (m, 3H, Ar-H), 6.44 (d, *J* = 7.8 Hz, 1H, Ar-H), 6.31 (d, *J* = 7.6 Hz, 1H, Ar-H), 6.06 (s, 1H, N-CH-S), 5.46 (d, *J* = 9.8 Hz, 1H, N-CH), 4.41–4.22 (m, 1H, CF_3_-CH), 2.26 (s, 3H, CH_3_), 1.64 (s, 9H, C(CH_3_)_3_); ^13^C NMR (101 MHz, CDCl_3_) *δ* 197.2 (C=O), 172.9 (C=O), 148.5, 147.1, 138.9, 135.5, 134.5, 132.7, 129.4, 128.8, 127.2, 126.0, 124.5 (q, *J* = 279.3 Hz, 1C, CF_3_), 124.0, 123.8, 123.5, 121.9, 121.8, 109.0, 85.7 (C_Boc_), 80.5 (N-C-S), 66.3 (N-C), 62.2 (C-CONBoc), 55.1 (q, *J* = 28.6 Hz, 1C, CF_3_-C), 27.9 ((CH_3_)_3_), 19.8 (CH_3_); IR (neat) *ν* 3056, 3011, 2984, 2965, 2923, 1735, 1697, 1596, 1577, 1473, 1448, 1396, 1368, 1353, 1296, 1272, 1248, 1203, 1177, 1144, 1116, 1033, 999, 971, 843, 781, 756, 744, 696 cm^−1^; HRMS (ESI) *m*/*z*: [M + H]^+^ calcd for C_31_H_28_F_3_N_2_O_4_S 581.1716; found 581.1721.
*Tert-butyl 1-benzoyl-5′,7′-dimethyl-2′-oxo-2-(trifluoromethyl)-1,2-dihydro-3aH-spiro[benzo[d]pyrrolo[2,1-b]thiazole-3,3′-indoline]-1′-carboxylate* (**5ag**): Eluent: petroleum ether/EtOAc = 40:1, white solid (46.4 mg, 78% yield); R*_f_* = 0.37 (petroleum ether/EtOAc = 20:1); m.p. 168.7–170.7 °C; ^1^H NMR (300 MHz, CDCl_3_) *δ* 8.17 (d, *J* = 7.2 Hz, 2H, Ar-H), 7.71 (dd, *J* = 7.4 Hz, 1H, Ar-H), 7.65–7.54 (m, 2H, Ar-H), 7.03–6.94 (m, 1H, Ar-H), 6.91 (s, 1H, Ar-H), 6.84–6.69 (m, 2H, Ar-H), 6.49 (d, *J* = 7.8 Hz, 1H, Ar-H), 6.00 (s, 2H, Ar-H, N-CH-S), 5.46 (d, *J* = 9.6 Hz, 1H, N-CH), 4.42–4.18 (m, 1H, CF_3_-CH), 2.21 (s, 3H, CH_3_), 1.93 (s, 3H, CH_3_), 1.63 (s, 9H, C(CH_3_)_3_); ^13^C NMR (75 MHz, CDCl_3_) *δ* 197.1 (C=O), 173.1 (C=O), 148.6, 147.5, 136.6, 135.6, 134.4, 133.3, 133.2, 129.4, 128.8, 127.3, 126.0, 124.6 (q, *J* = 279.1 Hz, 1C, CF_3_), 124.6, 124.0, 123.6, 121.8, 121.7, 108.9, 85.4 (C_Boc_), 80.6 (N-C-S), 66.5 (N-C), 62.5 (C-CONBoc), 54.6 (q, *J* = 28.4 Hz, 1C, CF_3_-C), 27.99 ((CH_3_)_3_), 20.8 (CH_3_), 19.7 (CH_3_); IR (neat) *ν* 3072, 2983, 2930, 1754, 1727, 1696, 1596, 1578, 1466, 1446, 1401, 1371, 1324, 1272, 1262, 1251, 1219, 1179, 1146, 1124, 1000, 971, 838, 827, 747, 696, 642 cm^−1^; HRMS (ESI) *m*/*z*: [M + H]^+^ calcd for C_32_H_30_F_3_N_2_O_4_S 595.1873; found 595.1876.
*1-Benzoyl-2-(trifluoromethyl)-1,2-dihydro-3aH-spiro[benzo[d]pyrrolo[2,1-b]thiazole-3,3′-indolin]-2′-one* (**5ah**): Eluent: petroleum ether/EtOAc = 4:1, white solid (42.0 mg, 90% yield); R*_f_* = 0.20 (petroleum ether/EtOAc = 6:1); m.p. 158.7–161.7 °C, ^1^H NMR (300 MHz, CDCl_3_) *δ* 9.41 (s, 1H, NH), 8.20 (d, *J* = 7.4 Hz, 2H, Ar-H), 7.72 (dd, *J* = 7.3 Hz, 1H, Ar-H), 7.66–7.53 (m, 2H, Ar-H), 7.24 (dd, *J* = 7.7 Hz, 1H, Ar-H), 7.10–6.90 (m, 2H, Ar-H), 6.87–6.65 (m, 3H, Ar-H), 6.51 (d, *J* = 7.8 Hz, 1H, Ar-H), 6.41 (d, *J* = 7.6 Hz, 1H, Ar-H), 6.02 (s, 1H, N-CH-S), 5.56 (d, *J* = 9.4 Hz, 1H, N-CH), 4.49–4.21 (m, 1H, CF_3_-CH); ^13^C NMR (75 MHz, CDCl_3_) *δ* 197.3 (C=O), 175.3 (C=O), 147.3, 141.4, 135.6, 134.4, 129.5, 129.4, 128.8, 127.0, 126.4, 126.0, 124.8 (q, *J* = 279.0 Hz, 1C, CF_3_), 124.2, 122.0, 121.9, 121.8, 110.7, 109.0, 80.1 (N-C-S), 66.1 (N-C), 62.2 (C-CONH), 53.7 (q, *J* = 28.6 Hz, 1C, CF_3_-C); IR (neat) *ν* 3205, 3098, 3063, 2959, 2929, 1716, 1680, 1618, 1580, 1467, 1395, 1272, 1237, 1213, 1204, 1170, 1125, 1110, 1072, 1027, 1013, 964, 743, 738, 734, 698, 683 cm^−1^;HRMS (ESI) *m*/*z*: [M + H]^+^ calcd for C_25_H_18_F_3_N_2_O_2_S 467.1036; found 467.1038.
*1′-Benzoyl-2′-(trifluoromethyl)-1′,2′-dihydro-2H,3a’H-spiro[benzofuran-3,3′-benzo[d]pyrrolo[2,1-b]thiazol]-2-one* (**5ai**): Eluent: petroleum ether/EtOAc = 40:1, white solid (39.7 mg, 85% yield); R*_f_* = 0.37 (petroleum ether/EtOAc = 20:1); m.p. 171.9–173.8 °C; ^1^H NMR (300 MHz, CDCl_3_) *δ* 8.16 (d, *J* = 7.4 Hz, 2H, Ar-H), 7.73 (dd, *J* = 7.4 Hz, 1H, Ar-H), 7.67–7.54 (m, 2H, Ar-H), 7.32 (dd, *J* = 7.9 Hz, 1H, Ar-H), 7.16 (d, *J* = 8.1 Hz, 1H, Ar-H), 7.08–6.94 (m, 1H, Ar-H), 6.90–6.74 (m, 3H, Ar-H), 6.54 (d, *J* = 7.8 Hz, 1H, Ar-H), 6.34 (d, *J* = 7.7 Hz, 1H, Ar-H), 5.99 (s, 1H, N-CH-S), 5.54 (d, *J* = 9.1 Hz, 1H, N-CH), 4.51–4.26 (m, 1H, CF_3_-CH); ^13^C NMR (75 MHz, CDCl_3_) *δ* 196.3 (C=O), 173.6 (C=O), 153.8, 146.8, 135.5, 134.6, 130.6, 129.5, 128.8, 126.4, 126.3, 124.5 (q, *J* = 278.9 Hz, 1C), 123.8, 122.3, 122.1, 122.0, 111.3, 109.1, 80.7 (N-C-S), 65.6 (N-C), 60.8 (C-CO_2_), 54.4 (q, *J* = 28.6 Hz, 1C, CF_3_-C); IR (neat) *ν* 3080, 3056, 2960, 2938, 1796, 1685, 1620, 1592, 1469, 1446, 1397, 1356, 1346, 1275, 1255, 1228, 1207, 1166, 1155, 1139, 1126, 1093, 977, 811, 752, 740, 693, 688, 652 cm^−1^; HRMS (ESI) *m*/*z*: [M + H]^+^ calcd for C_25_H_17_F_3_NO_3_S 468.0876; found 468.0891.

### 3.5. General Experimental Procedures for Synthesis of Compounds ***7*** (Scheme 6)

In an ordinary vial charged with a magnetic stirring bar, *N*-phenacylbenzothiazolium bromide **1a** (0.15 mmol, 1.5 equiv), 2-benzylidenemalononitrile **6a**–**h** (0.1 mmol, 1.0 equiv), TEA (0.15 mmol, 1.5 equiv), and DEM (1.0 mL) were added. The mixture was then stirred at room temperature for 3 h. Products **7** were isolated with flash chromatography on silica gel.

*1-Benzoyl-2-phenyl-1,2-dihydrobenzo[d]pyrrolo[2,1-b]thiazole-3,3(3aH)-dicarbonitrile* (**7aa**): Eluent: petroleum ether/DCM = 1:2, white solid (38.7 mg, 95% yield); R*_f_* = 0.17 (petroleum ether/EtOAc = 6:1); m.p. 80.5–82.4 °C; ^1^H NMR (400 MHz, CDCl_3_) *δ* 7.94–7.87 (m, 2H, Ar-H), 7.65–7.58 (m, 1H, Ar-H), 7.55–7.49 (m, 2H, Ar-H), 7.48–7.43 (m, 2H, Ar-H), 7.43–7.39 (m, 3H, Ar-H), 7.24–7.16 (m, 1H, Ar-H), 7.10–6.99 (m, 1H, Ar-H), 6.97–6.86 (m, 1H, Ar-H), 6.48 (d, *J* = 7.7 Hz, 1H, Ar-H), 6.07 (s, 1H, N-CH-S), 5.59 (d, *J* = 9.3 Hz, 1H, N-CH), 4.36 (d, *J* = 9.3 Hz, 1H, CH); ^13^C NMR (101 MHz, CDCl_3_) *δ* 196.9 (C=O), 146.3, 135.1, 134.7, 130.4, 130.1, 129.7, 129.3, 128.9, 128.8, 126.7, 124.8, 122.9, 122.6, 112.3, 111.0, 109.8, 79.5 (N-C-S), 69.2 (N-C), 58.0 (C), 51.0 (CH); IR (neat) *ν* 3066, 2935, 2839, 2221, 1730, 1696, 1671, 1663, 1603, 1577, 1511, 1469, 1447, 1308, 1254, 1223, 1177, 1021, 832, 750, 688, 612 cm^−1^; HRMS (ESI) *m*/*z*: [M + H]^+^ calcd for C_25_H_18_N_3_OS 408.1165; found 408.1170.
*1-Benzoyl-2-(p-tolyl)-1,2-dihydrobenzo[d]pyrrolo[2,1-b]thiazole-3,3(3aH)-dicarbonitrile* (**7ab**): Eluent: petroleum ether/DCM = 1:2, white solid (36.7 mg, 87% yield); R*_f_* = 0.23 (petroleum ether/EtOAc = 6:1); m.p. 82.7–84.6 °C; ^1^H NMR (400 MHz, CDCl_3_) *δ* 7.91 (d, *J* = 7.7 Hz, 2H, Ar-H), 7.65–7.59 (m, 1H, Ar-H), 7.49–7.43 (m, 2H, Ar-H), 7.42–7.38 (m, 2H, Ar-H), 7.23–7.17 (m, 3H, Ar-H), 7.02 (dd, *J* = 7.6 Hz, 1H, Ar-H), 6.91 (dd, *J* = 7.4 Hz, 1H, Ar-H), 6.46 (d, *J* = 7.7 Hz, 1H, Ar-H), 6.06 (s, 1H, N-CH-S), 5.56 (d, *J* = 9.4 Hz, 1H, N-CH), 4.33 (d, *J* = 9.3 Hz, 1H, CH), 2.34 (s, 3H, CH_3_); ^13^C NMR (101 MHz, CDCl_3_) *δ* 197.1 (C=O), 146.3, 140.5, 135.2, 134.7, 130.3, 129.3, 128.8, 128.7, 126.9, 126.7, 124.8, 122.8, 122.6, 112.4, 111.1, 109.8, 79.4 (N-C-S), 69.1 (N-C), 57.9 (C), 51.2 (CH), 21.3 (CH_3_); IR (neat) *ν* 3066, 2926, 2224, 2210, 1733, 1669, 1581, 1469, 1448, 1341, 1301, 1222, 1178, 1093, 1014, 828, 748, 686cm^−1^; HRMS (ESI) *m*/*z*: [M + H]^+^ calcd for C_26_H_20_N_3_OS 422.1322; found 422.1326.
*1-Benzoyl-2-(4-methoxyphenyl)-1,2-dihydrobenzo[d]pyrrolo[2,1-b] thiazole-3,3(3aH)-dicarbonitrile* (**7ac**): Eluent: petroleum ether/DCM = 1:2, white solid (41.1 mg, 94% yield); R*_f_* = 0.23 (petroleum ether/EtOAc = 6:1); m.p. 86.3–87.9 °C; ^1^H NMR (400 MHz, CDCl_3_) *δ* 7.90 (d, *J* = 7.6 Hz, 2H, Ar-H), 7.64–7.59 (m, 1H, Ar-H), 7.49–7.41 (m, 4H, Ar-H), 7.19 (d, *J* = 7.6 Hz, 1H, Ar-H), 7.05–6.98 (m, 1H, Ar-H), 6.94–6.88 (m, 3H, Ar-H), 6.44 (d, *J* = 7.9 Hz, 1H, Ar-H), 6.05 (s, 1H, N-CH-S), 5.52 (d, *J* = 9.2 Hz, 1H, N-CH), 4.30 (d, *J* = 9.5 Hz, 1H, CH), 3.79 (s, 3H, OCH_3_); ^13^C NMR (101 MHz, CDCl_3_) *δ* 197.2 (C=O), 161.1, 146.4, 135.2, 134.7, 130.2, 129.3, 128.8, 126.7, 124.9, 122.8, 122.6, 121.6, 115.0, 112.4, 111.2, 109.8, 79.3 (N-C-S), 69.3 (N-C), 57.8 (C), 55.5 (OCH_3_), 51.2 (CH); IR (neat) *ν* 3065, 2926, 2227, 2207, 1734, 1669, 1593, 1581, 1513, 1489, 1469, 1448, 1344, 1302, 1254, 1222, 1178, 1073, 1010, 822, 747, 687, 660 cm^−1^; HRMS (ESI) *m*/*z*: [M + H]^+^ calcd for C_26_H_20_N_3_O_2_S 438.1271; found 438.1274.
*1-Benzoyl-2-(m-tolyl)-1,2-dihydrobenzo[d]pyrrolo[2,1-b]thiazole-3,3(3aH)-dicarbonitrile* (**7ad**): Eluent: petroleum ether/DCM = 1:2, white solid (37.5 mg, 89% yield); R*_f_* = 0.32 (petroleum ether/EtOAc = 6:1); m.p. 101.3–102.8 ^o^C; ^1^H NMR (400 MHz, CDCl_3_) *δ* 7.92–7.88 (m, 2H, Ar-H), 7.64–7.58 (m, 1H, Ar-H), 7.49–7.43 (m, 2H, Ar-H), 7.36–7.32 (m, 1H, Ar-H), 7.32–7.26 (m, 2H, Ar-H), 7.22–7.18 (m, 2H, Ar-H), 7.03 (td, *J* = 7.7, 1.3 Hz, 1H, Ar-H), 6.92 (td, *J* = 7.6, 1.1 Hz, 1H, Ar-H), 6.46 (d, *J* = 7.8 Hz, 1H, Ar-H), 6.06 (s, 1H, N-CH-S), 5.56 (d, *J* = 9.4 Hz, 1H, N-CH), 4.31 (d, *J* = 9.4 Hz, 1H, CH), 2.33 (s, 3H, CH_3_); ^13^C NMR (101 MHz, CDCl_3_) *δ* 197.1 (C=O), 146.3, 139.6, 135.2, 134.7, 131.2, 130.0, 129.9, 129.5, 129.3, 128.8, 126.7, 125.6, 124.9, 122.9, 122.6, 112.4, 111.1, 109.8, 79.5 (N-C-S), 69.2 (N-C), 58.0 (C), 51.0 (CH), 21.5 (CH_3_); IR (neat) *ν* 3064, 2955, 2920, 2850, 2228, 2214, 1682, 1579, 1465, 1448, 1296, 1245, 1224, 1210, 1180, 1027, 1001, 964, 737, 705, 686, 660 cm^−1^; HRMS (ESI) *m*/*z*: [M + H]^+^ calcd for C_26_H_20_N_3_OS 422.1322; found 422.1325.
*1-Benzoyl-2-(3,4-dimethylphenyl)-1,2-dihydrobenzo[d]pyrrolo[2,1-b]thiazole-3,3(3aH)-dicarbonitrile* (**7ae**): Eluent: petroleum ether/DCM = 1:2, white solid (42.2 mg, 97% yield); R*_f_* = 0.26 (petroleum ether/EtOAc = 6:1); m.p. 88.5–90.4 °C; ^1^H NMR (400 MHz, CDCl_3_) *δ* 7.94–7.90 (m, 2H, Ar-H), 7.64–7.59 (m, 1H, Ar-H), 7.49–7.44 (m, 2H, Ar-H), 7.29–7.26 (m, 1H, Ar-H), 7.21–7.17 (m, 2H, Ar-H), 7.17–7.14 (m, 1H, Ar-H), 7.02 (td, *J* = 7.7, 1.3 Hz, 1H, Ar-H), 6.91 (td, *J* = 7.6, 1.1 Hz, 1H, Ar-H), 6.46 (d, *J* = 7.7 Hz, 1H, Ar-H), 6.05 (s, 1H, N-CH-S), 5.55 (d, *J* = 9.5 Hz, 1H, N-CH), 4.30 (d, *J* = 9.5 Hz, 1H, CH), 2.23 (s, 6H, (CH_3_)_2_); ^13^C NMR (101 MHz, CDCl_3_) *δ* 197.2 (C=O), 146.4, 139.2, 138.1, 135.3, 134.6, 130.8, 130.3, 129.2, 128.8, 127.2, 126.7, 125.8, 124.9, 122.8, 122.6, 112.5, 111.1, 109.8, 79.4 (N-C-S), 69.1 (N-C), 57.9 (C), 51.2 (CH), 19.9 (CH_3_), 19.7 (CH_3_); IR (neat) *ν* 3066, 3037, 2934, 2231, 2205, 1738, 1671, 1593, 1582, 1469, 1448, 1303, 1219, 1179, 1135, 1031, 824, 747, 686 cm^−1^; HRMS (ESI) *m*/*z*: [M + H]^+^ calcd for C_27_H_22_N_3_OS 436.1478; found 436.1472.
*1-Benzoyl-2-(naphthalen-2-yl)-1,2-dihydrobenzo[d]pyrrolo[2,1-b]thiazole-3,3(3aH)-dicarbonitrile* (**7af**): Eluent: petroleum ether/DCM = 1:2, white solid (41.6 mg, 91% yield); R*_f_* = 0.36 (petroleum ether/EtOAc = 6:1); m.p. 125.1–126.4 °C; ^1^H NMR (400 MHz, CDCl_3_) *δ* 7.79–7.76 (m, 2H, Ar-H), 7.72–7.68 (m, 1H, Ar-H), 7.55–7.51 (m, 1H, Ar-H), 7.39–7.35 (m, 2H, Ar-H), 7.35–7.30 (m, 3H, Ar-H), 7.30–7.22 (m, 2H, Ar-H), 7.21–7.13 (m, 2H, Ar-H), 7.00 (td, *J* = 7.7, 1.2 Hz, 1H, Ar-H), 6.88 (td, *J* = 7.6, 1.1 Hz, 1H, Ar-H), 6.46 (d, *J* = 7.9 Hz, 1H, Ar-H), 6.01 (s, 1H, N-CH-S), 5.42 (d, *J* = 9.0 Hz, 1H, N-CH), 5.20 (d, *J* = 8.9 Hz, 1H, CH); ^13^C NMR (101 MHz, CDCl_3_) *δ* 196.2 (C=O), 145.9, 136.1, 135.0, 134.6, 131.2, 130.8, 129.2, 129.1, 128.8, 128.7, 127.9, 126.8, 124.9, 123.1, 122.9, 112.0, 111.2, 109.7, 79.9 (N-C-S), 70.5 (N-C), 51.8 (C), 49.8 (CH); IR (neat) *ν* 3072, 2919, 2850, 2207, 1732, 1675, 1589, 1579, 1466, 1444, 1293, 1250, 1216, 1201, 1181, 1032, 855, 746, 703, 687 cm^−1^; HRMS (ESI) *m*/*z*: [M + H]^+^ calcd for C_29_H_20_N_3_OS 458.1322; found 458.1330.

## 4. Conclusions

In summary, we developed an efficient strategy for the [3 + 2] cycloaddition reactions of heteroaromatic *N*-ylides with different electron-deficient olefins for the diverse synthesis of fused polyheterocyclic compounds under mild reaction conditions. With the *N*-phenacylbenzothiazolium bromides as the heteroaromatic *N*-ylides precursors, the heteroaromatic *N*-ylides can smoothly react with maleimides to give fused polycyclic octahydropyrrolo[3,4-*c*]pyrroles in moderate to excellent isolated yields (52–99%). Moreover, this [3 + 2] cycloaddition concept could also be extended to 3-trifluoroethylidene oxindoles and benzylidenemalononitriles as electron-deficient olefins, affording a series of structurally diverse functionalized polyheterocyclic compounds in good to excellent yields (70–97%). Further studies about exploring benzothiazolium salts to synthesize other new structurally unique *N*,*S*-polyheterocycle compounds are currently underway in our laboratory.

## Figures and Tables

**Table 1:** Optimization of reaction conditions [a].

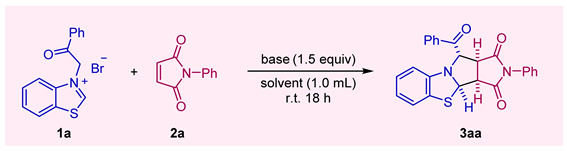			
Entry	Base	Solvent	Yield (%) [b]
1	Cs_2_CO_3_	DCM	63
2	K_3_PO_4_	DCM	88
3	Na_2_CO_3_	DCM	95
4	K_2_HPO_4_	DCM	75
5	TEA	DCM	n.d.
6	Na_2_CO_3_	toluene	99
7	Na_2_CO_3_	DCE	75
8	Na_2_CO_3_	MeCN	33
9	Na_2_CO_3_	THF	n.d.

[a] The reaction was carried out with **1a** (0.15 mmol), **2a** (0.10 mmol), and base (0.15 mmol) in solvent (1.0 mL) at room temperature for the specified reaction time. [b] Isolated yield. n.d.—not detected.

## Data Availability

All the required data are reported in the manuscript and Supplementary Materials.

## Supplementary Materials

The following supporting information can be downloaded at: https://www.mdpi.com/article/10.3390/molecules28114410/s1, X-ray data for products **3aa**; copies of ^1^H, ^13^C NMR spectra. References [69,70,71] are cited in the supplementary materials.

## Sample Availability

Samples of the compounds are available from the authors.
