# Supplementary material for: Diverse Synthesis of Fused Polyheterocyclic Compounds via [3 + 2] Cycloaddition of In Situ-Generated Heteroaromatic N-Ylides and Electron-Deficient Olefins
Source: Molecules. 2023 May 29;28(11):4410. doi: 10.3390/molecules28114410 (PMC10254280; doi:10.3390/molecules28114410)

Supporting Information

Diverse Synthesis of Fused Polyheterocyclic Compounds via [3 + 2] Cycloaddition of in Situ Generated Heteroaromatic *N*-Ylides and Electron-Deficient Olefins

Zhen-Hua Wang,*[,1] Tong Zhang,[1] Li-Wen Shen,[2] Xiu Yang,[1] Yan-Ping Zhang,[1] Yong You,[1] Jian-Qiang Zhao,[1] and Wei-Cheng Yuan*[,1]

[1]Innovation Research Center of Chiral Drugs, Institute for Advanced Study, Chengdu University, Chengdu 610106, China

[2]College of Chemistry and Chemical Engineering, Zunyi Normal College, Zunyi 563006, China

wangzhenhua@cdu.edu.cn

yuanwc@cioc.ac.cn

Table of Contents

1. Optimization of the [3 + 2] cycloaddition reaction of benzothiazolium salt with other olefinic dipolarophiles 2

2. X-ray crystal structure of **3aa** 3

3. ^{1}H, ^{13}C NMR for compounds **3**, **5**, and **7** 5

1. Optimization of the [3 + 2] cycloaddition reaction of benzothiazolium salt with other olefinic dipolarophiles

Table S1 Optimization of reaction conditions for the [3 + 2] cycloaddition reaction between benzothiazolium salt and 3-trifluoroethylidene oxindole[a]

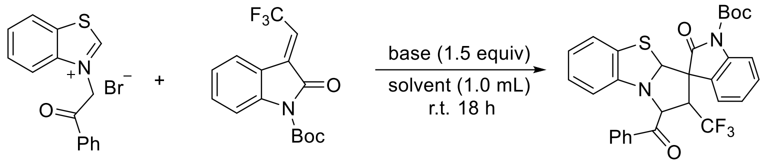

1a **4a** **5aa**

entry	base	solvent	yield[b]
1	Cs_2CO_3	DCM	95
2	K_3PO_4	DCM	51
4	DIPEA	DCM	85
5	TEA	DCM	69
6	Cs_2CO_3	Toluene	84
7	Cs_2CO_3	DCE	88
8	Cs_2CO_3	MeCN	71
9	Cs_2CO_3	THF	86

[a]The reaction was carried out with **1a** (0.15 mmol), **4a** (0.10 mmol), base (0.15 mmol) in solvent (1.0 mL) at room temperature for indicated time. [b]Isolated yield.

Table S2 Optimization of reaction conditions for the [3 + 2] cycloaddition reaction of benzothiazolium salt with benzylidenemalononitrile[a]

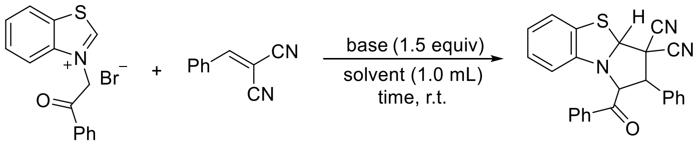

1a **6a** **7aa**

entry	base	solvent	time (h)	yield (%)[b]
1	Cs_2CO_3	THF	3	45
2	Cs_2CO_3	MeCN	2	43
3	Cs_2CO_3	toluene	24	12
4	Cs_2CO_3	EtOH	24	n.d.
5	Cs_2CO_3	$CHCl_3$	3	9
6	Cs_2CO_3	2-MeTHF	24	25
7	Cs_2CO_3	1,4-dioxane	3	46
8	Cs_2CO_3	DME	3	51
9	Cs_2CO_3	MTBE	3	32
10	K_3PO_4	DME	3	44
11	K_2CO_3	DME	3	94
12	Na_2CO_3	DME	3	93
13	Li_2CO_3	DME	3	n.d.

14	$NaHCO_3$	DME	3	72
15	NaOH	DME	3	25
16	DIPEA	DME	3	71
17	TEA	DME	3	95
18[c]	TEA	DME	3	72

[a] The reaction was carried out with **1a** (0.15 mmol), **6a** (0.10 mmol), base (0.15 mmol) in solvent (1.0 mL) at room temperature for indicated time. [b] Isolated yield. [c] 2.0 mL 1,2-dimethoxyethane (DME) was used.

2. X-ray crystal structure of 3aa

Single crystals of compound **3aa** were prepared through dissolving the sample in mixture solvent of EtOH at room temperature and crystalizing by slow evaporation of solvent. A suitable crystal was selected for structure determination on an 'Oxford Gemini E' diffractometer. The crystal was kept at 293 K during data collection. Using Olex2[1], the structure was solved with the ShelXT[2] structure solution program using Intrinsic Phasing and refined with the ShelXL[3] refinement package using Least Squares minimisation.

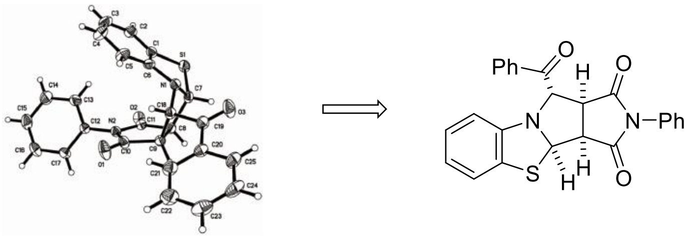

3aa (CCDC 2193494)

ORTEP of **3aa** (at the 50% probability level)

Identification code	**3aa**
Empirical formula	$C_{25}H_{18}N_2O_3S$
Formula weight	426.47
Temperature/K	293(2)
Crystal system	monoclinic
Space group	$P2_1/c$
a/Å	10.4876(6)
b/Å	8.2188(4)
c/Å	23.8232(9)
α/°	90
β/°	101.322(4)
γ/°	90
Volume/Å^3	2013.49(18)
Z	4
ρ_{calc}g/cm^3	1.407
μ/mm^{-1}	1.686

F(000)	888.0
Crystal size/mm^3	0.12 × 0.1 × 0.09
Radiation	CuKα (λ = 1.54184)
2Θ range for data collection/°	7.57 to 134.16
Index ranges	$-12 \le h \le 10$, $-9 \le k \le 6$, $-26 \le l \le 28$
Reflections collected	7242
Independent reflections	3593 [R_{int} = 0.0337, R_{sigma} = 0.0476]
Data/restraints/parameters	3593/0/280
Goodness-of-fit on F^2	1.033
Final R indexes [I>=2σ (I)]	R_1 = 0.0477, wR_2 = 0.1282
Final R indexes [all data]	R_1 = 0.0600, wR_2 = 0.1426
Largest diff. peak/hole / e Å^{-3}	0.23/-0.28

1. Dolomanov, O. V.; Bourhis, L. J.; Gildea, R. J, Howard, J. A. K; Puschmann, H. *J. Appl. Cryst.*, **2009**, *42*, 339-341.
2. Sheldrick, G. M. *Acta Cryst*. **2015**, A71, 3-8.
3. Sheldrick, G. M. *Acta Cryst.* **2015**, C71, 3-8.

3. ^{1}H, ^{13}C NMR for compounds 3, 5, and 7

^{1}H NMR (400 MHz, $CDCl_3$) of **3aa**

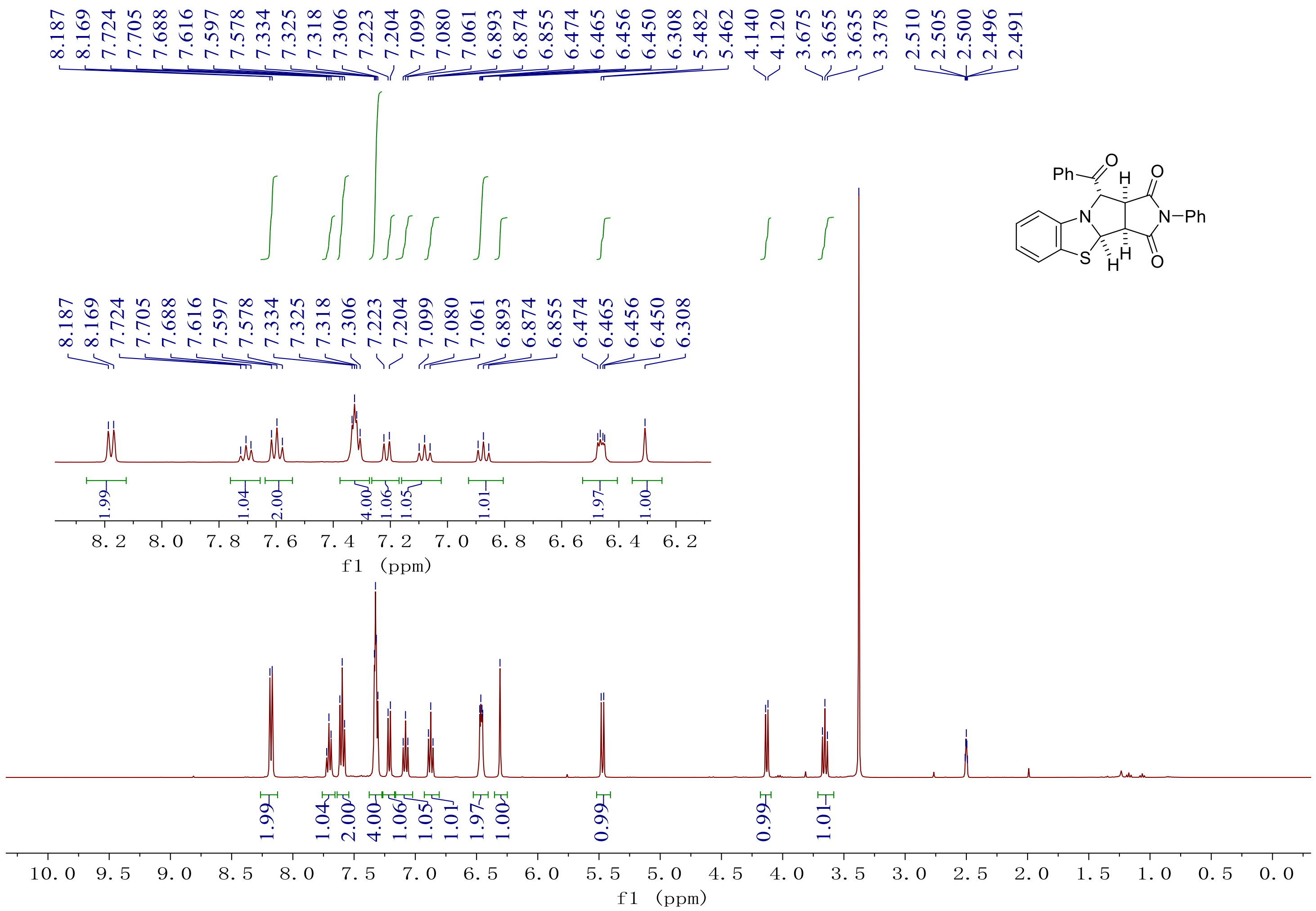

^{13}C NMR (101 MHz, $CDCl_3$) of **3aa**

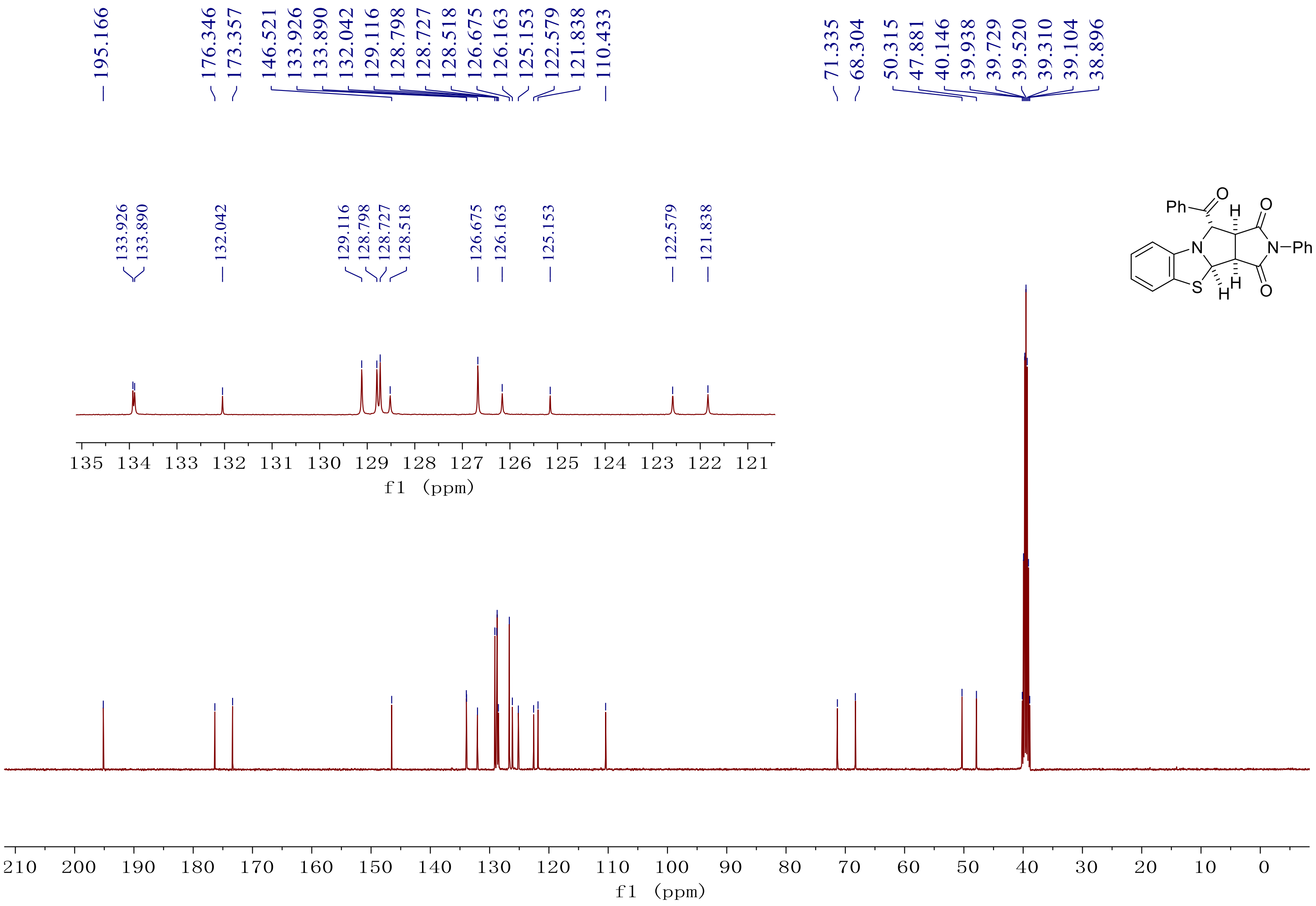

^{1}H NMR (400 MHz, $CDCl_3$) of **3ba**

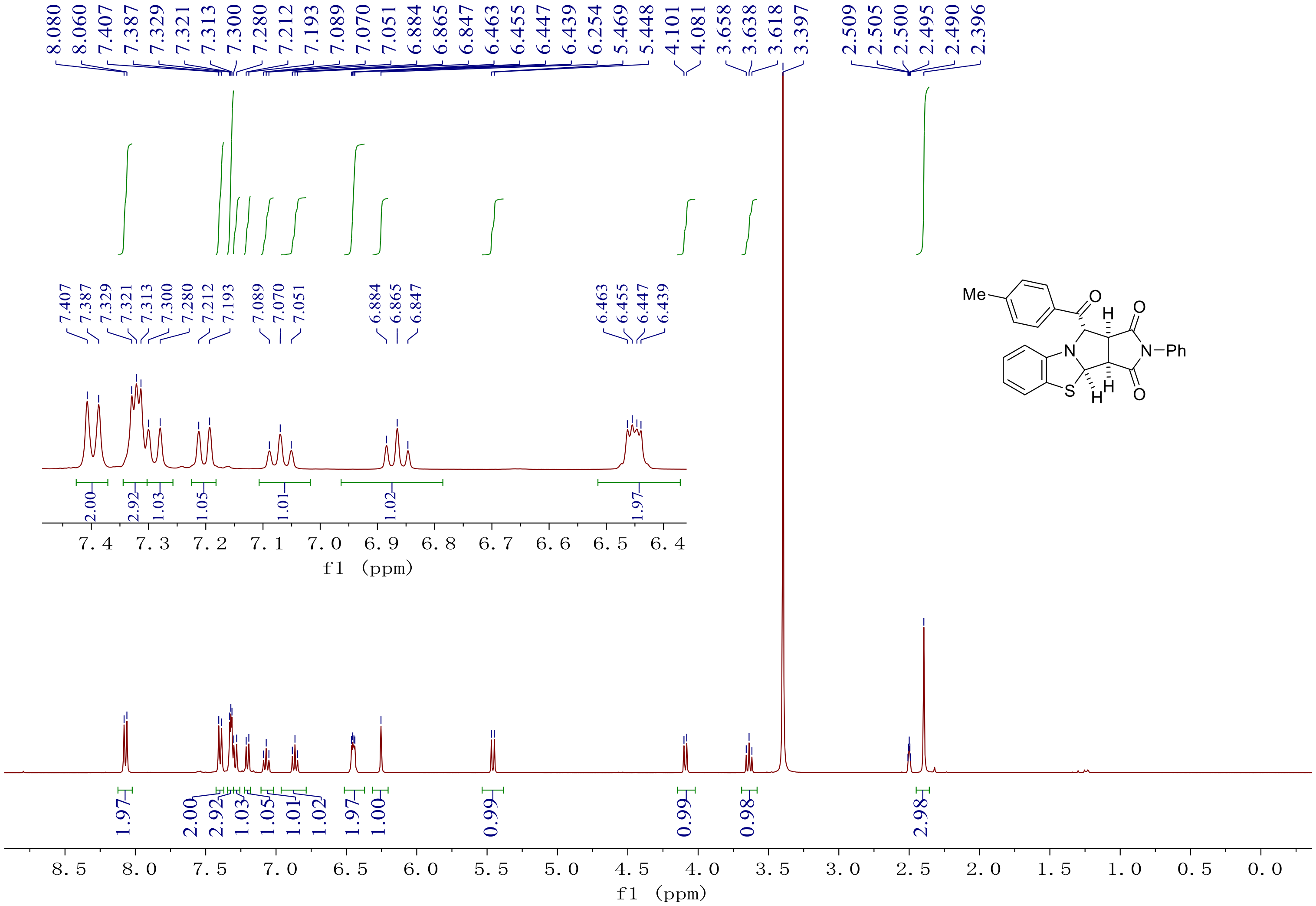

^{13}C NMR (101 MHz, $CDCl_3$) of **3ba**

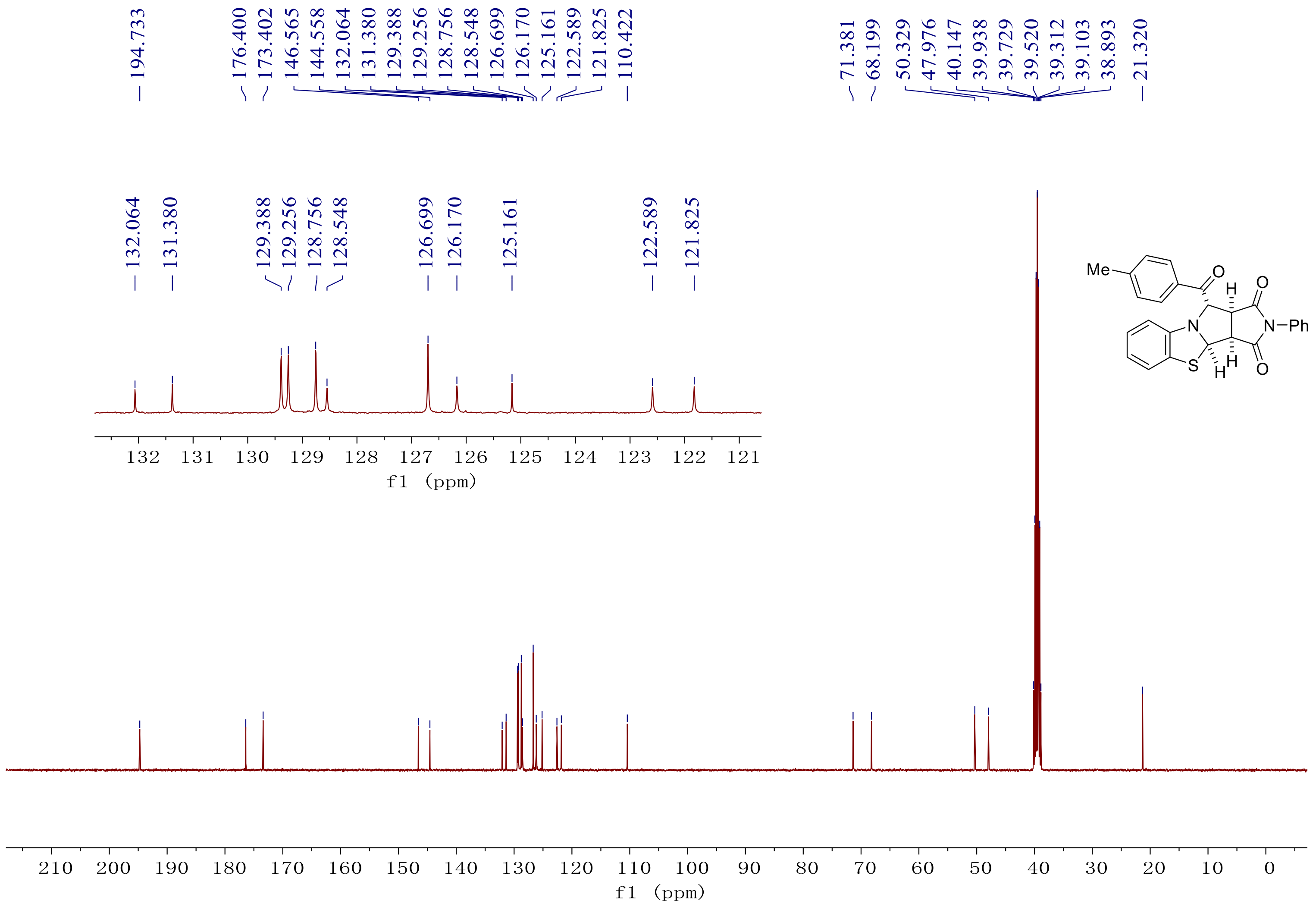

^{1}H NMR (400 MHz, $CDCl_3$) of **3ca**

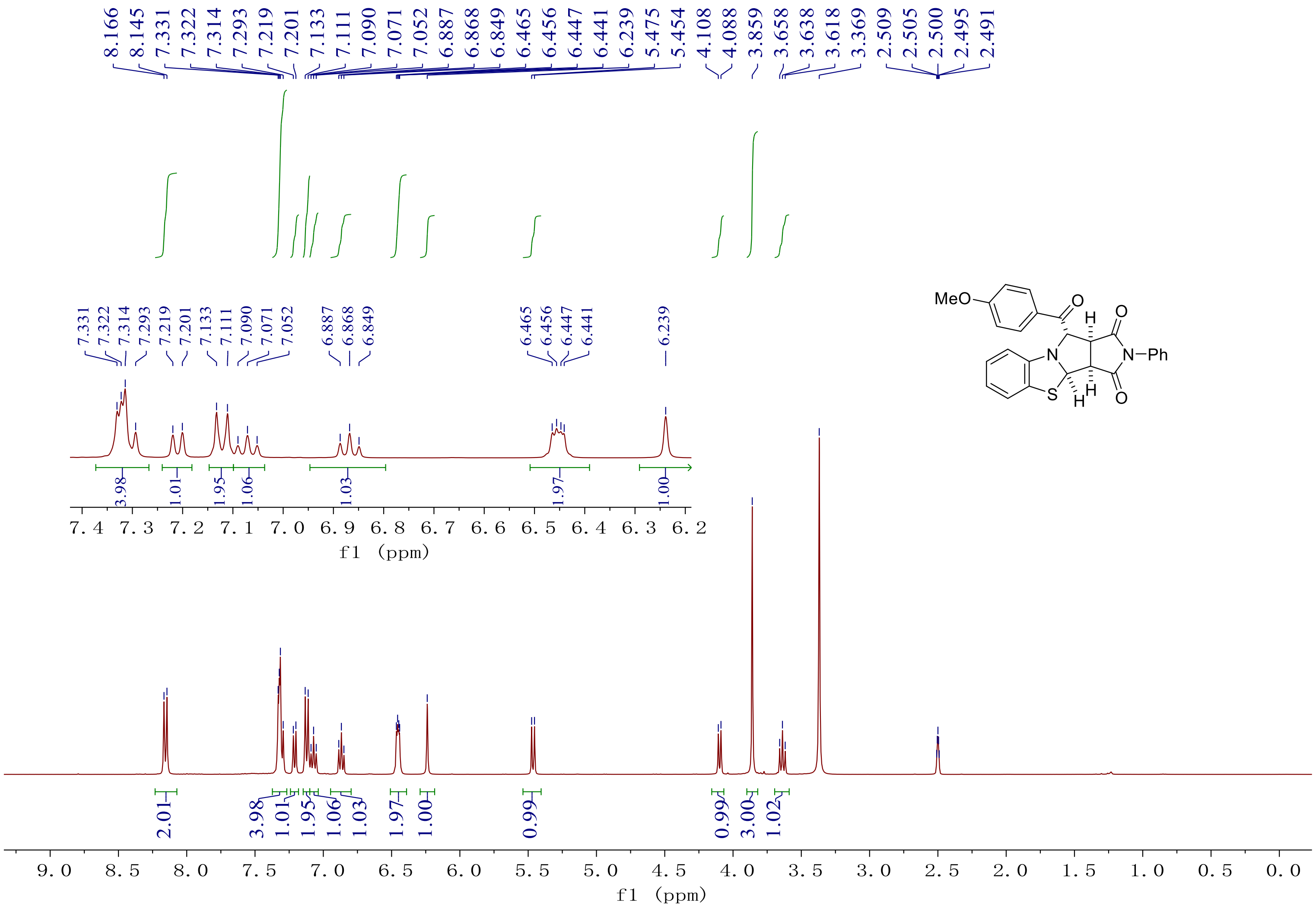

^{13}C NMR (101 MHz, $CDCl_3$) of **3ca**

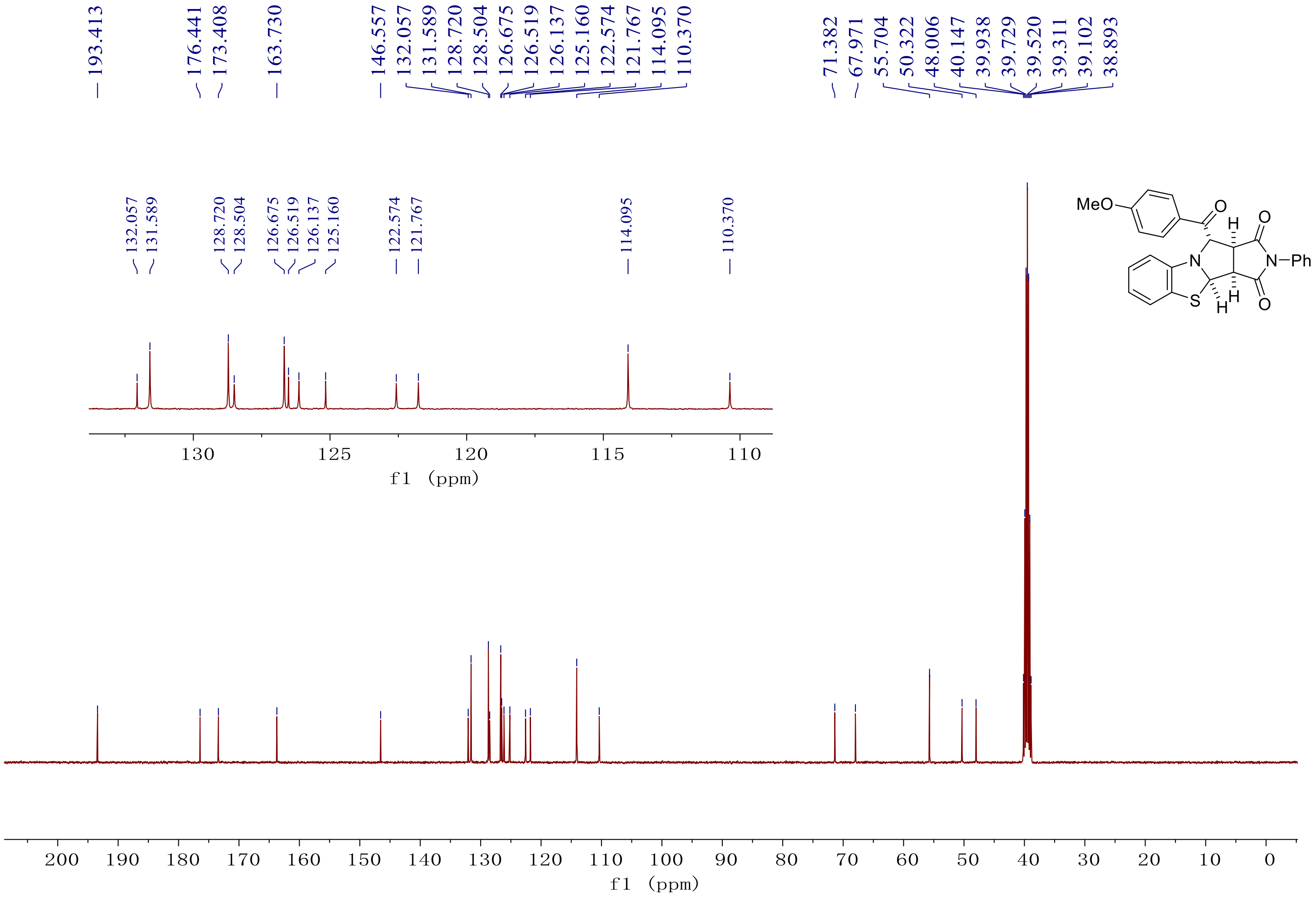

^{1}H NMR (400 MHz, $CDCl_3$) of **3da**

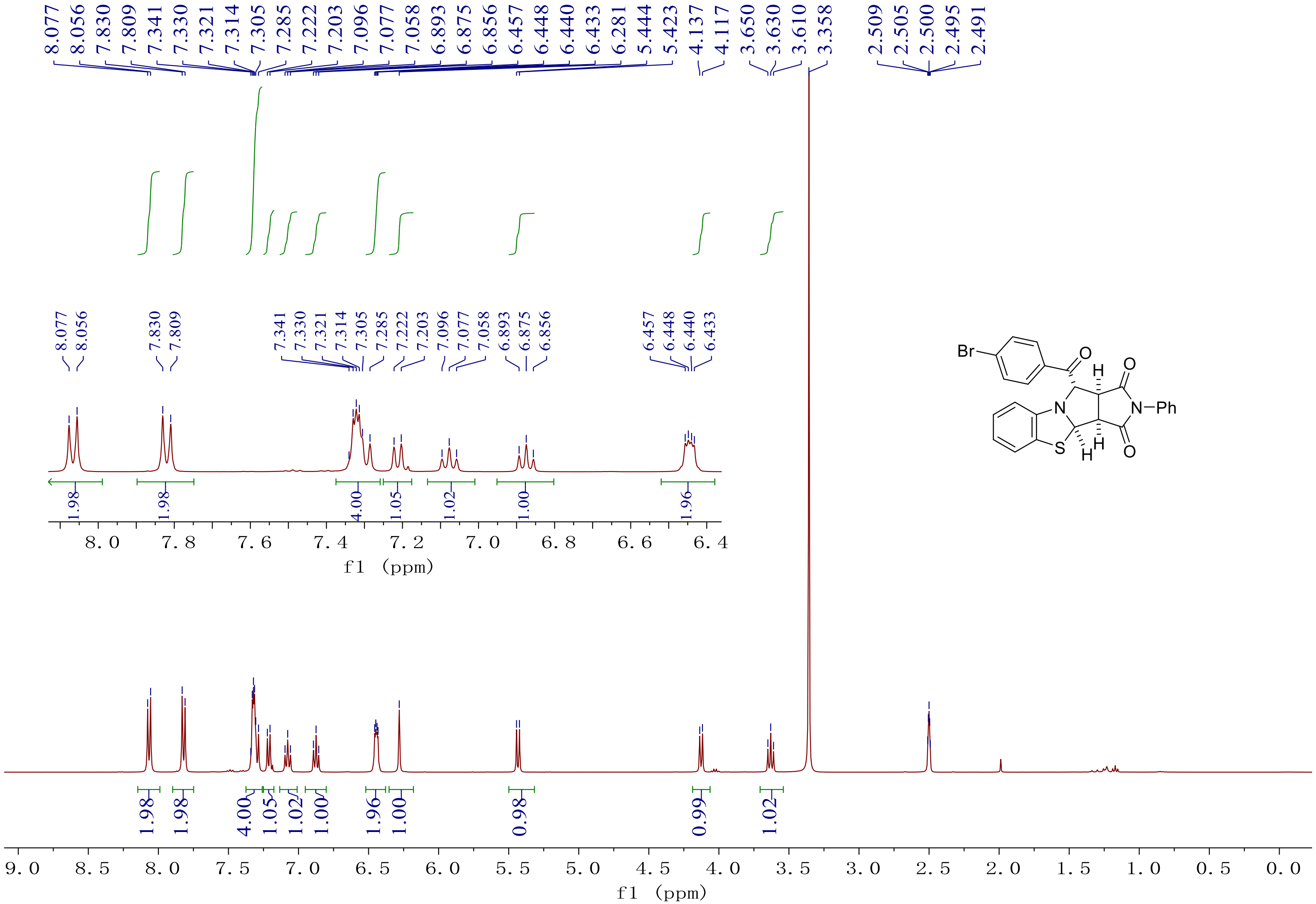

^{13}C NMR (101 MHz, CDCl$_3$) of **3da**

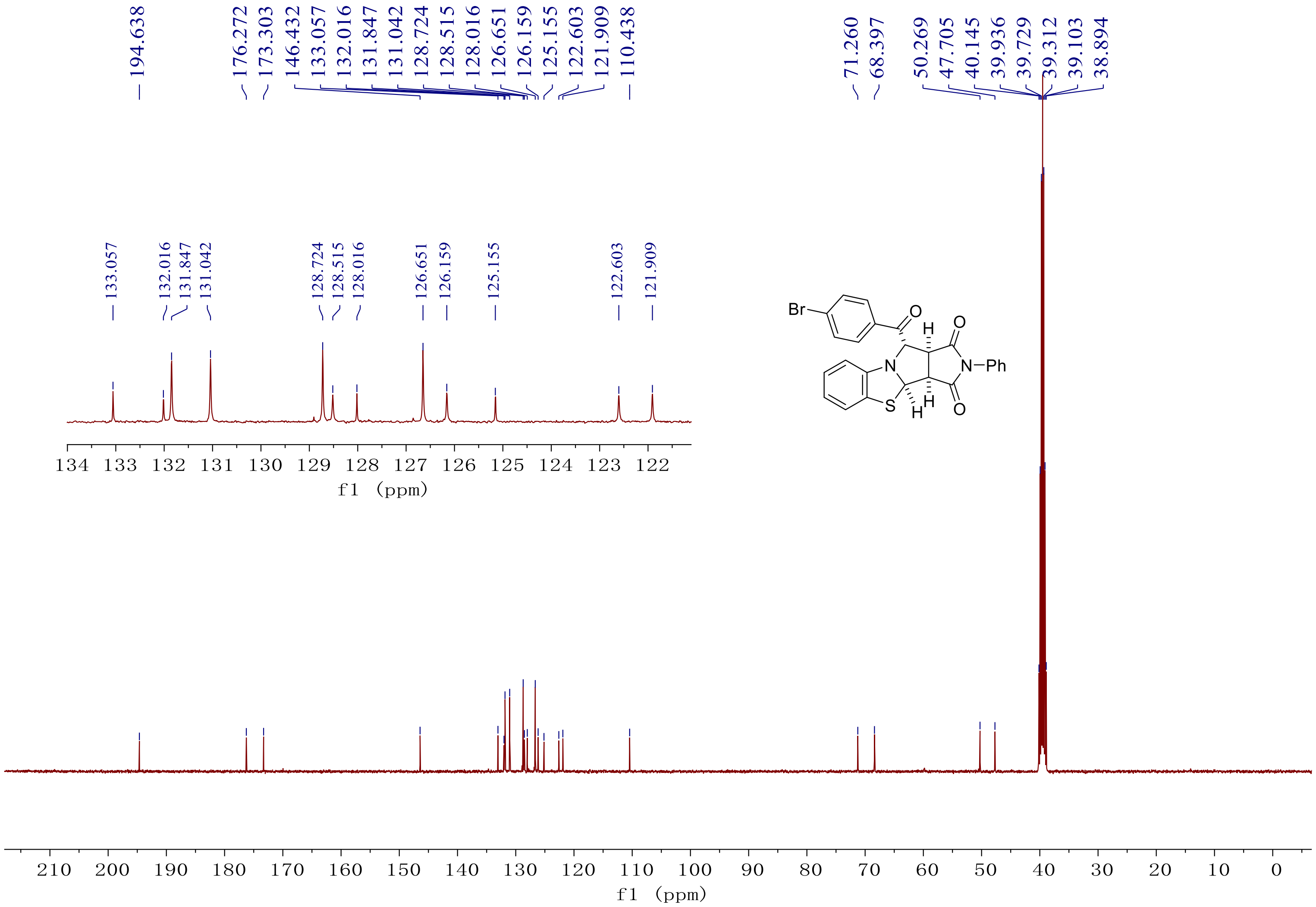

^{1}H NMR (400 MHz, $CDCl_3$) of **3ea**

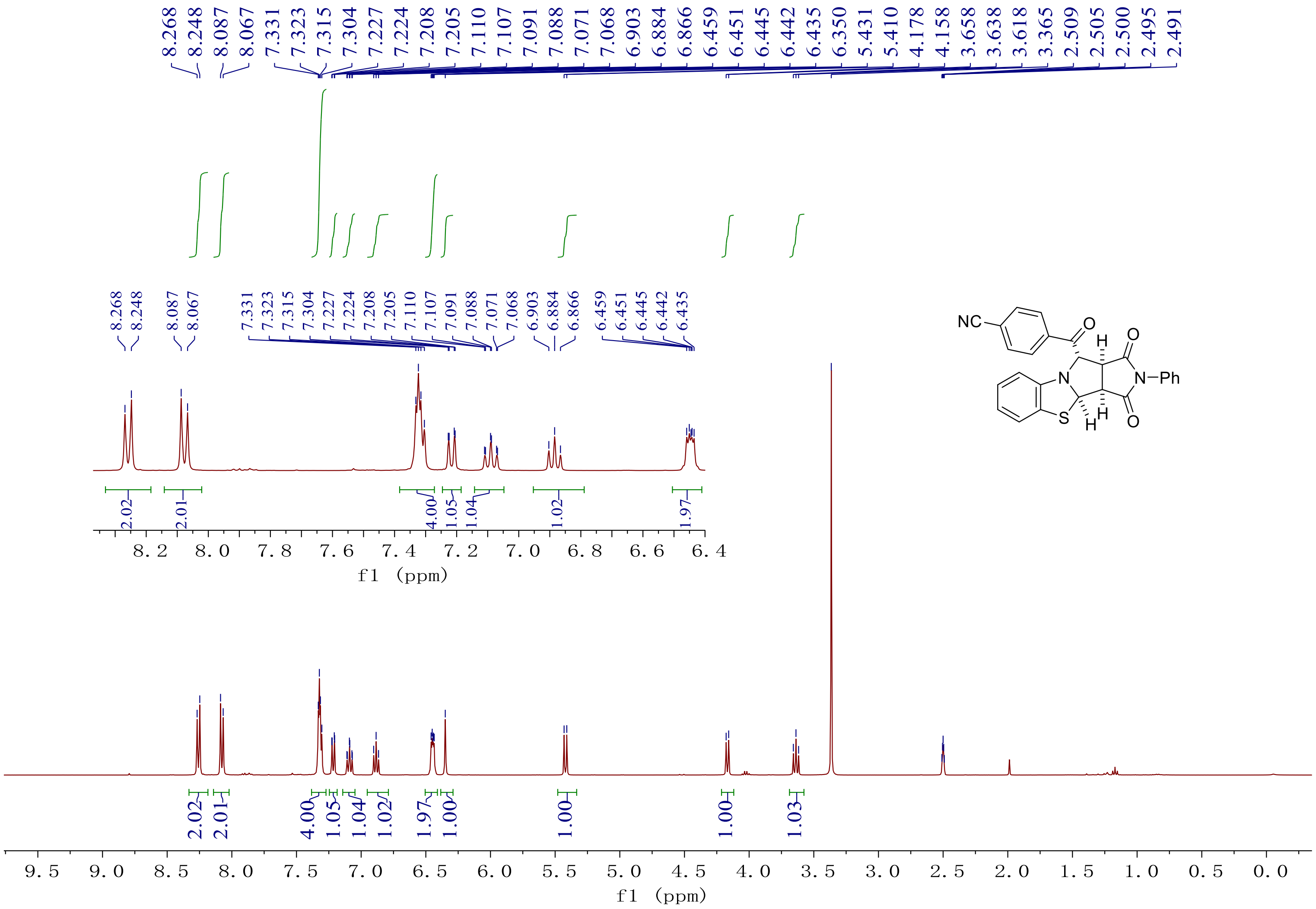

^{13}C NMR (101 MHz, CDCl$_3$) of **3ea**

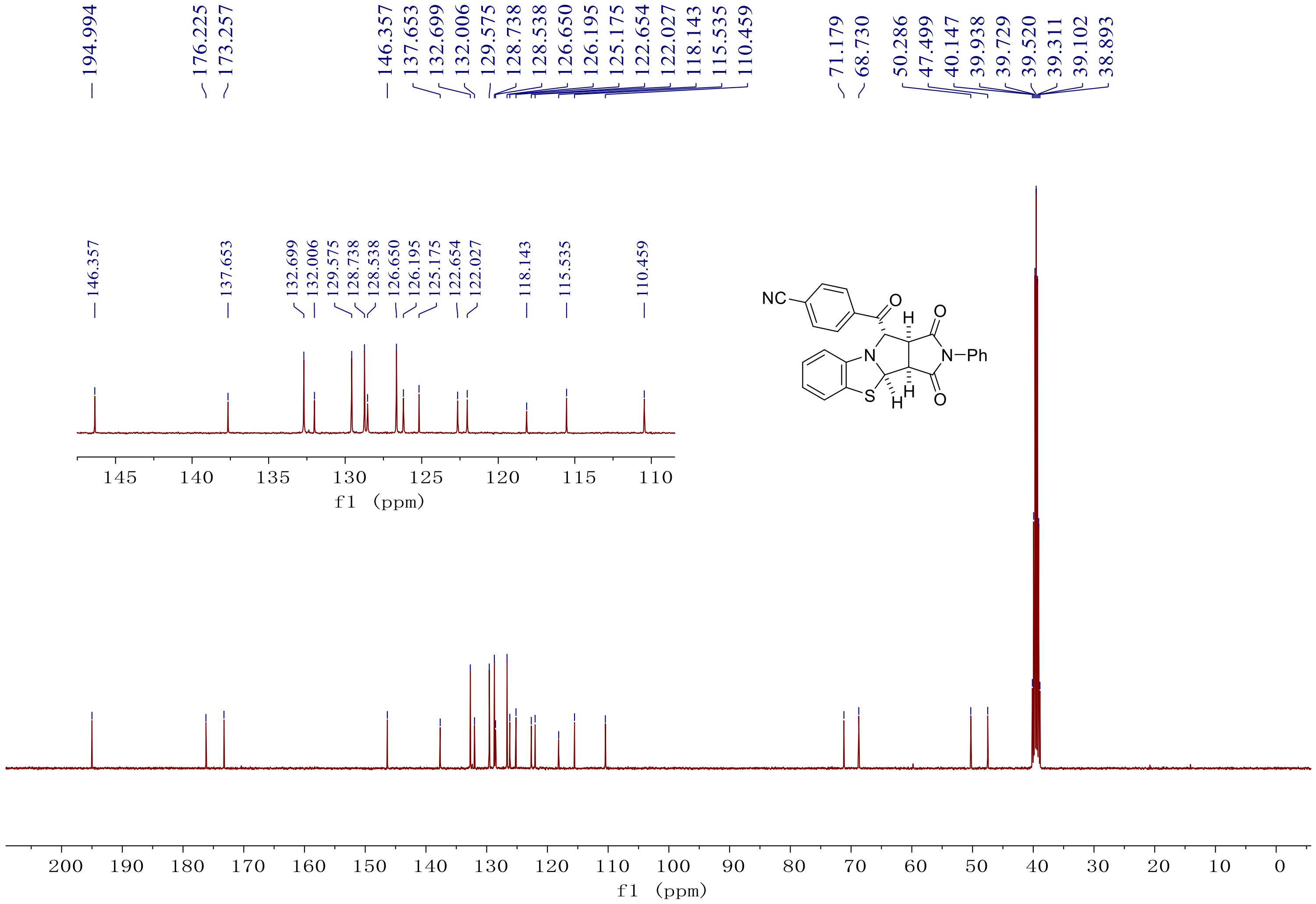

^{1}H NMR (400 MHz, CDCl3) of **3fa**

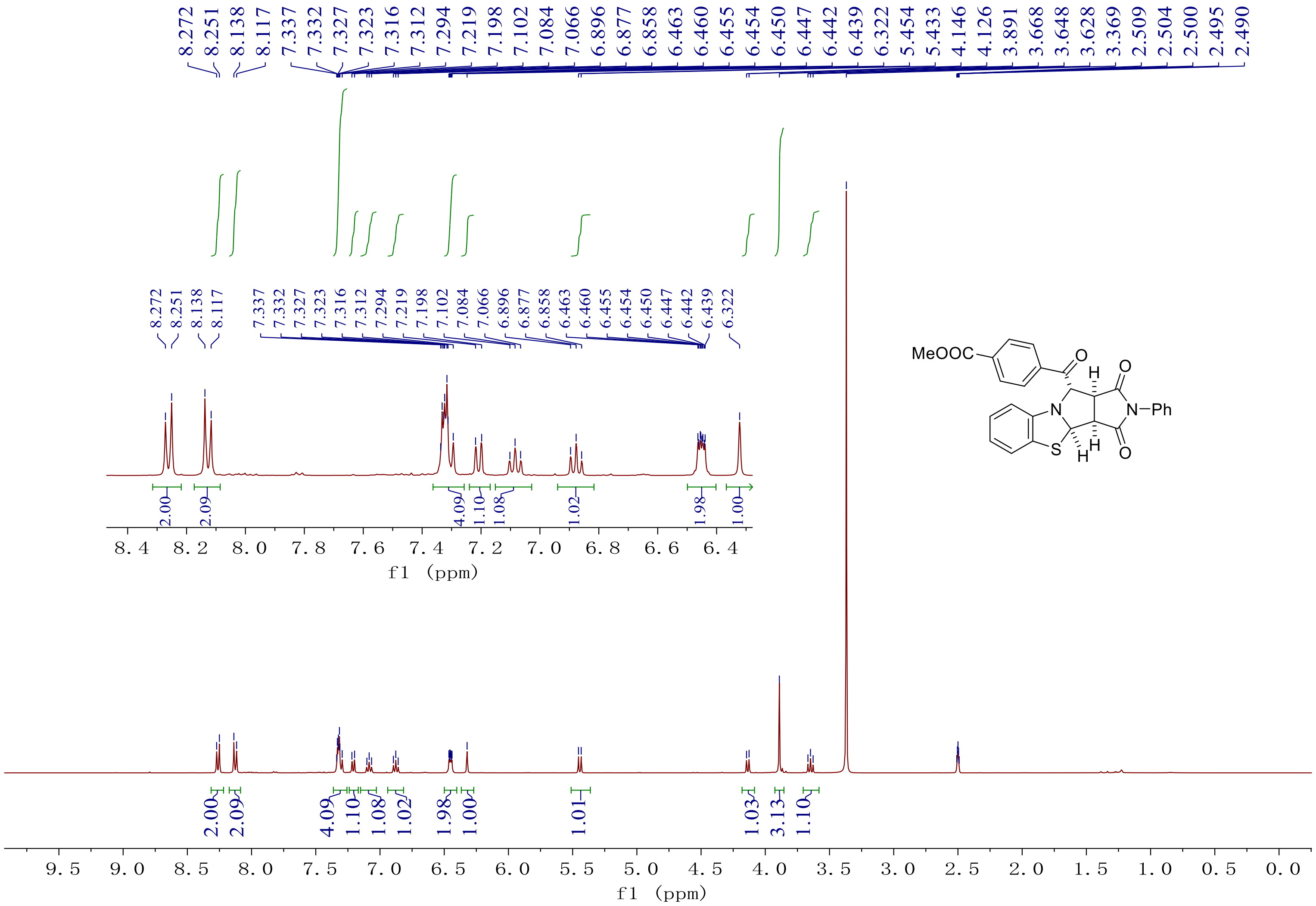

^{13}C NMR (101 MHz, $CDCl_3$) of **3fa**

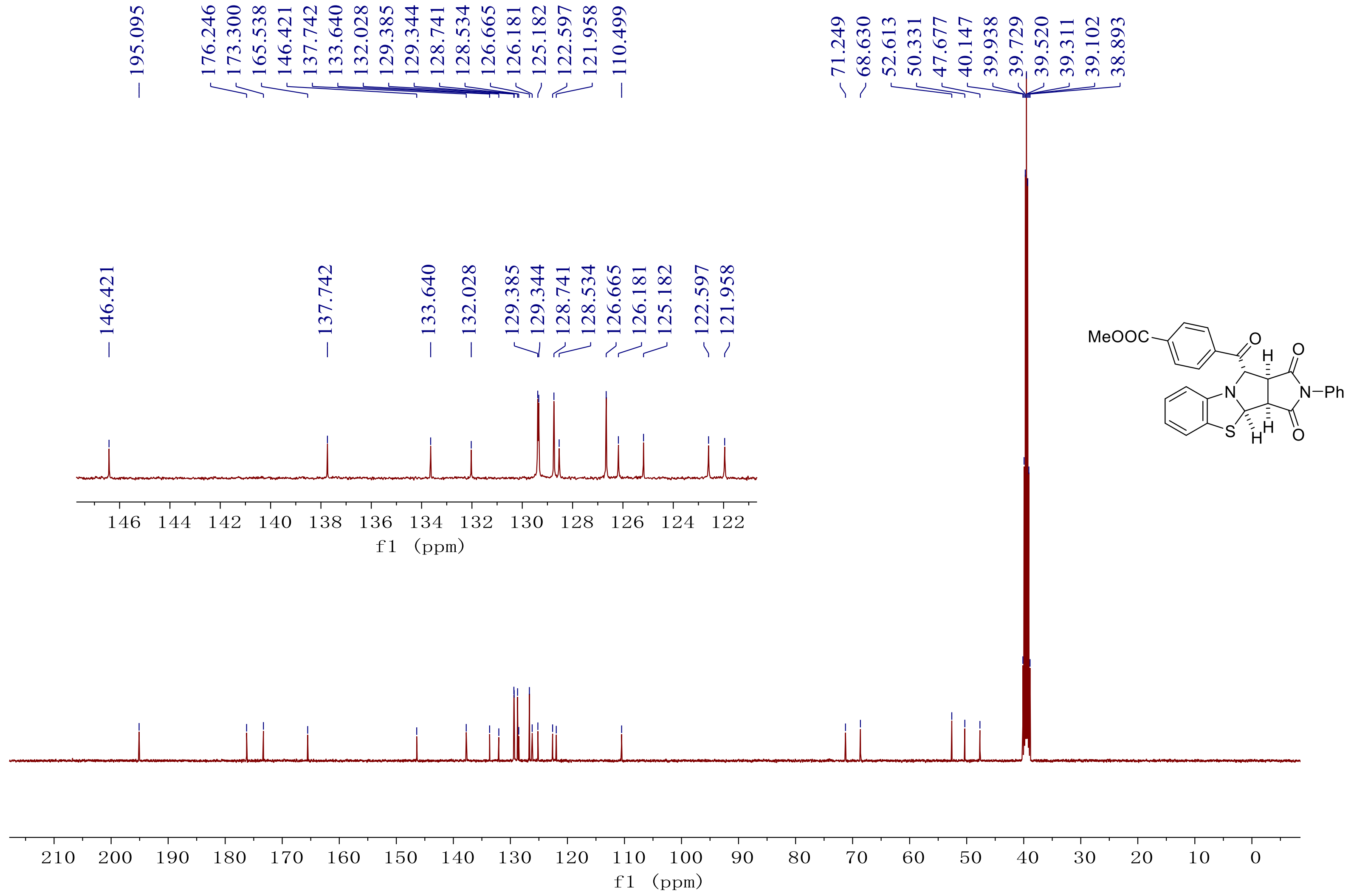

^{1}H NMR (400 MHz, $CDCl_3$) of **3ga**

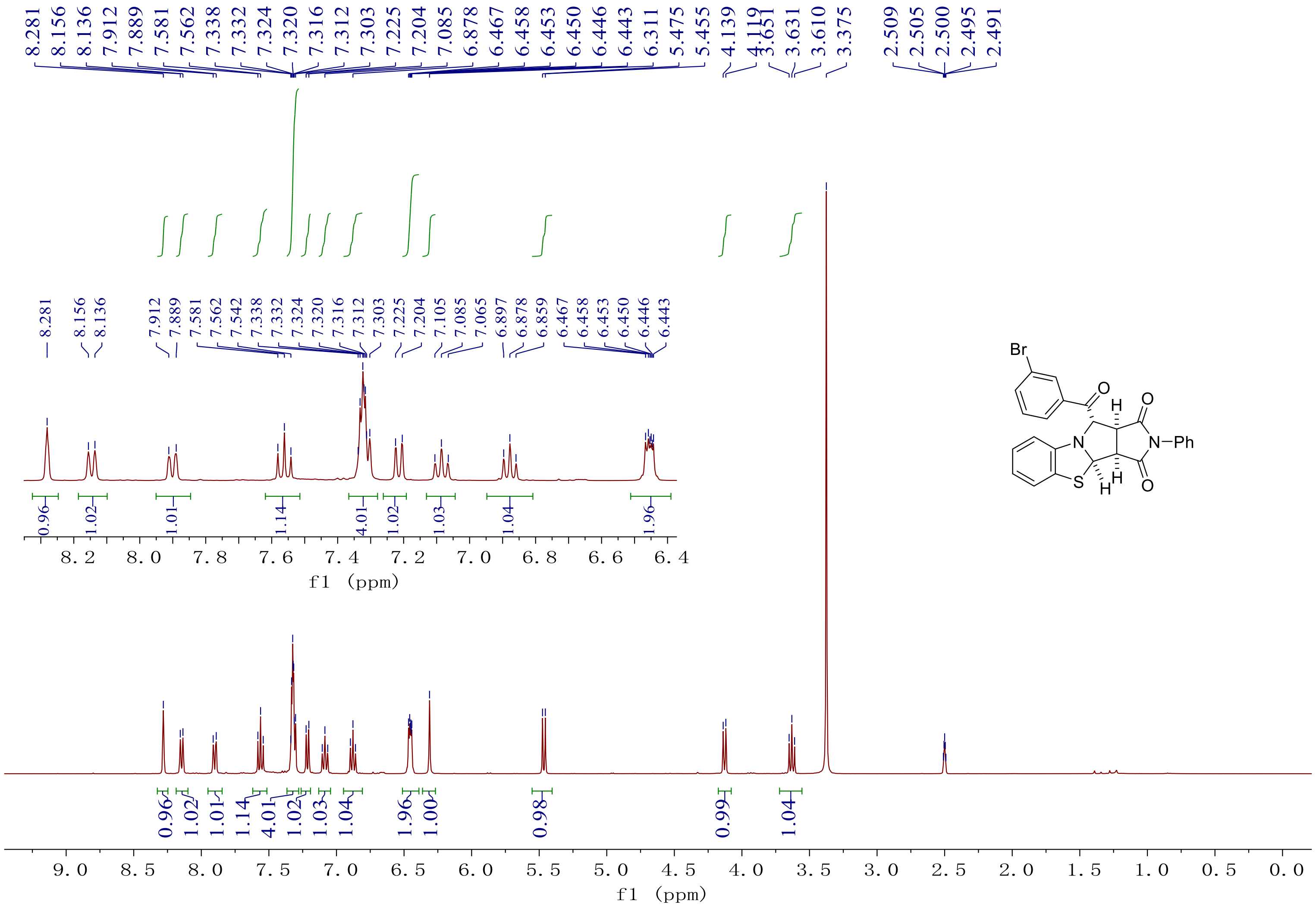

^{13}C NMR (101 MHz, $CDCl_3$) of **3ga**

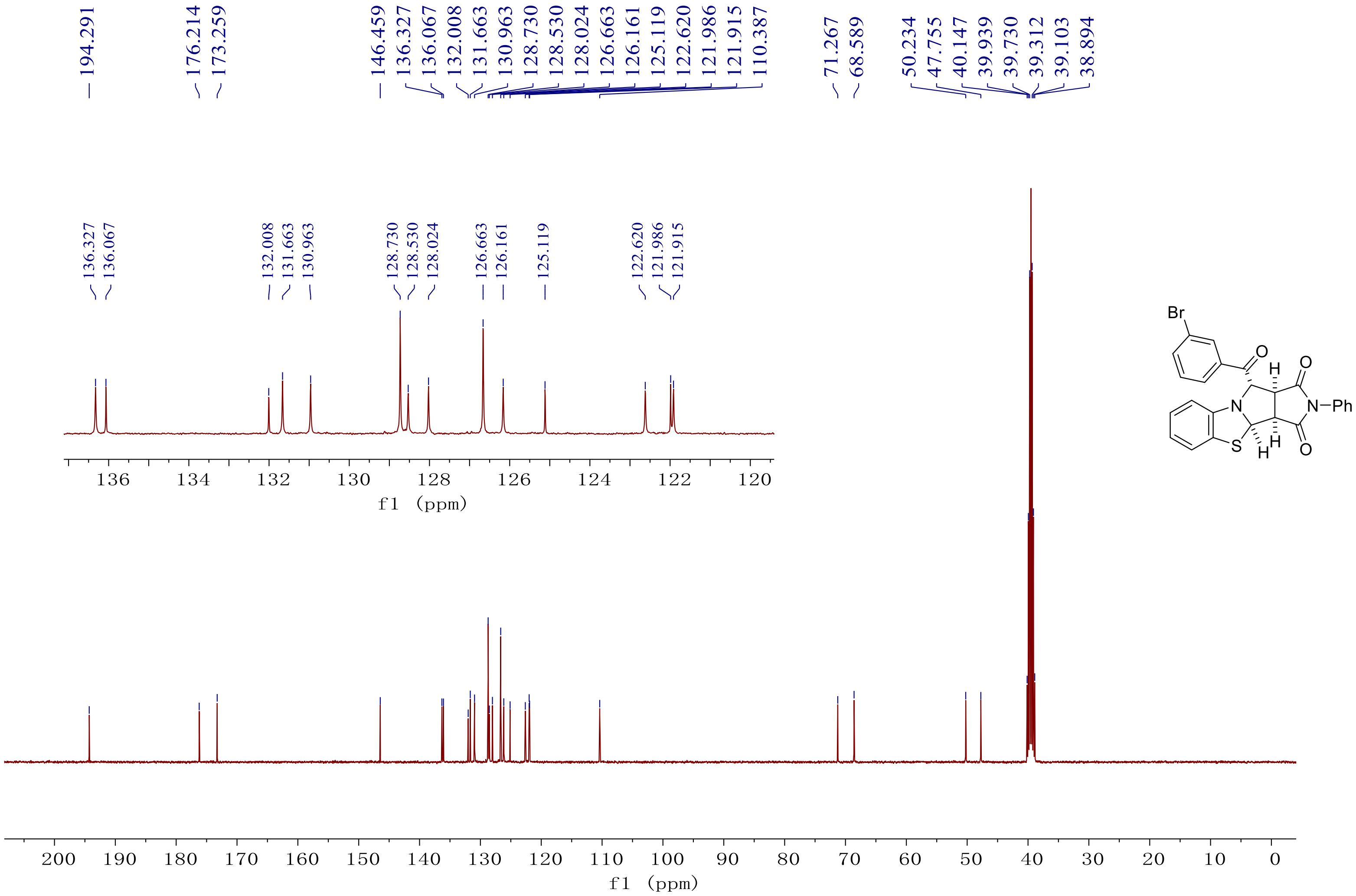

^{1}H NMR (400 MHz, $CDCl_3$) of **3ha**

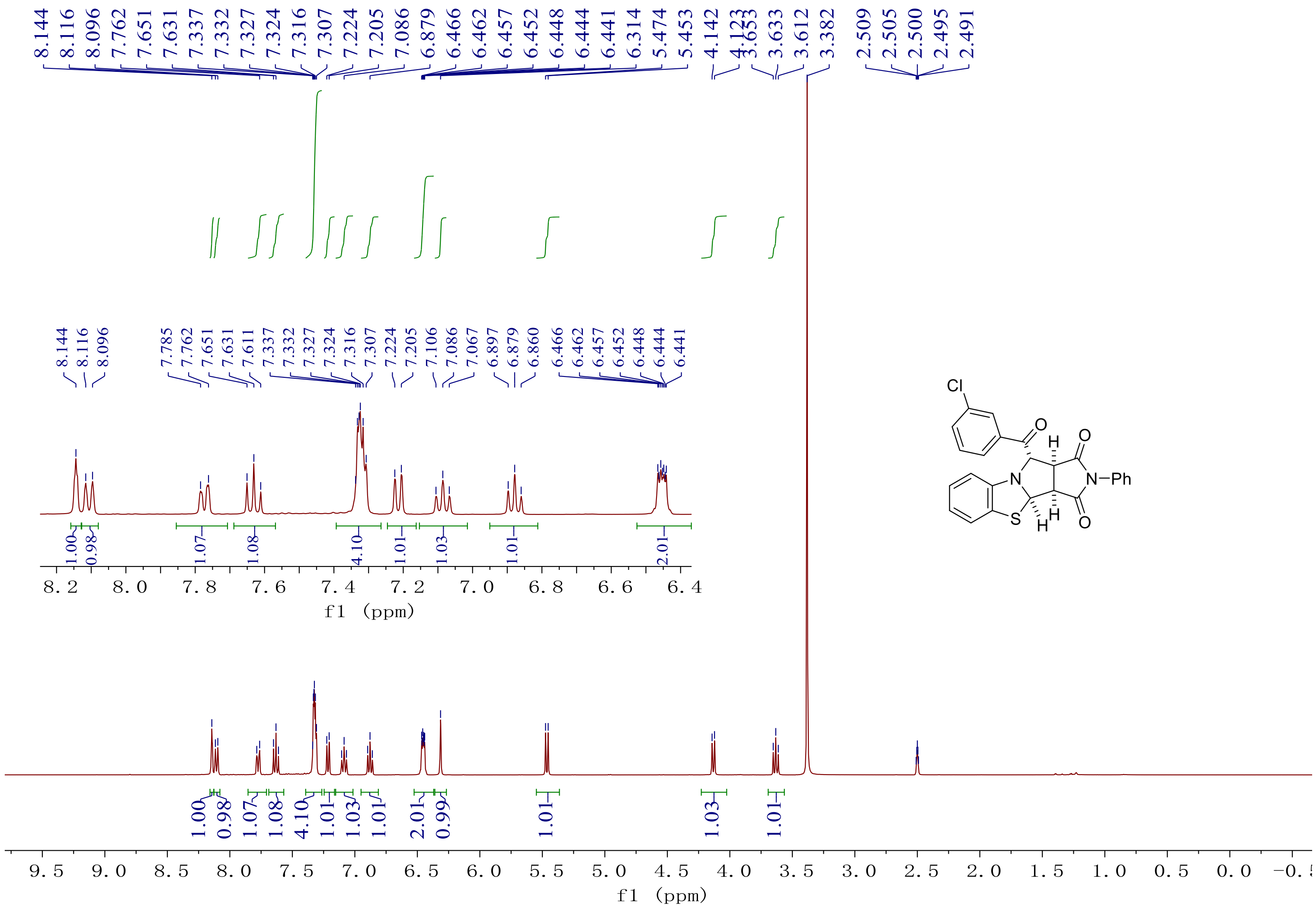

^{13}C NMR (101 MHz, $CDCl_3$) of **3ha**

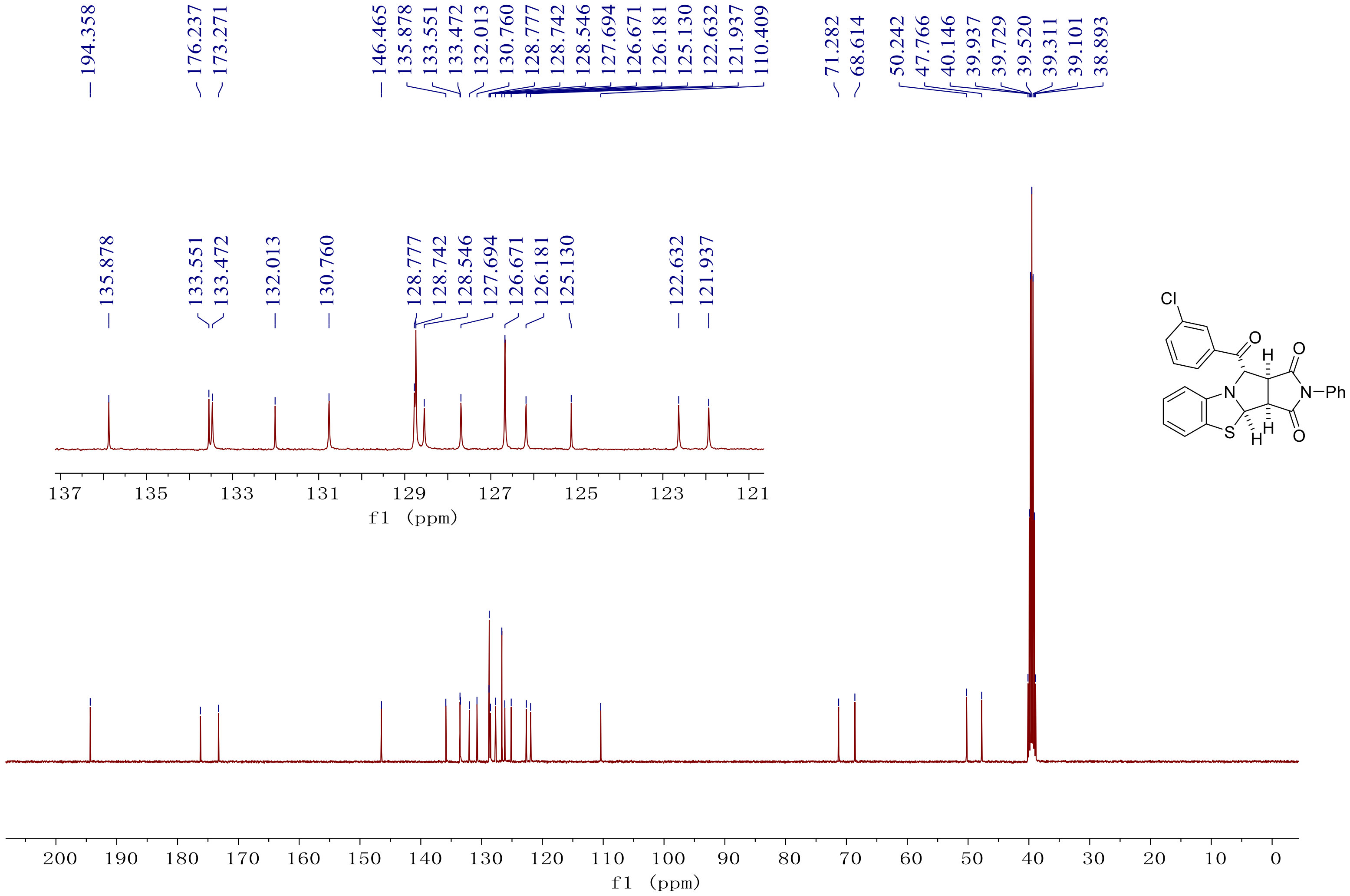

^{1}H NMR (400 MHz, $CDCl_3$) of **3ia**

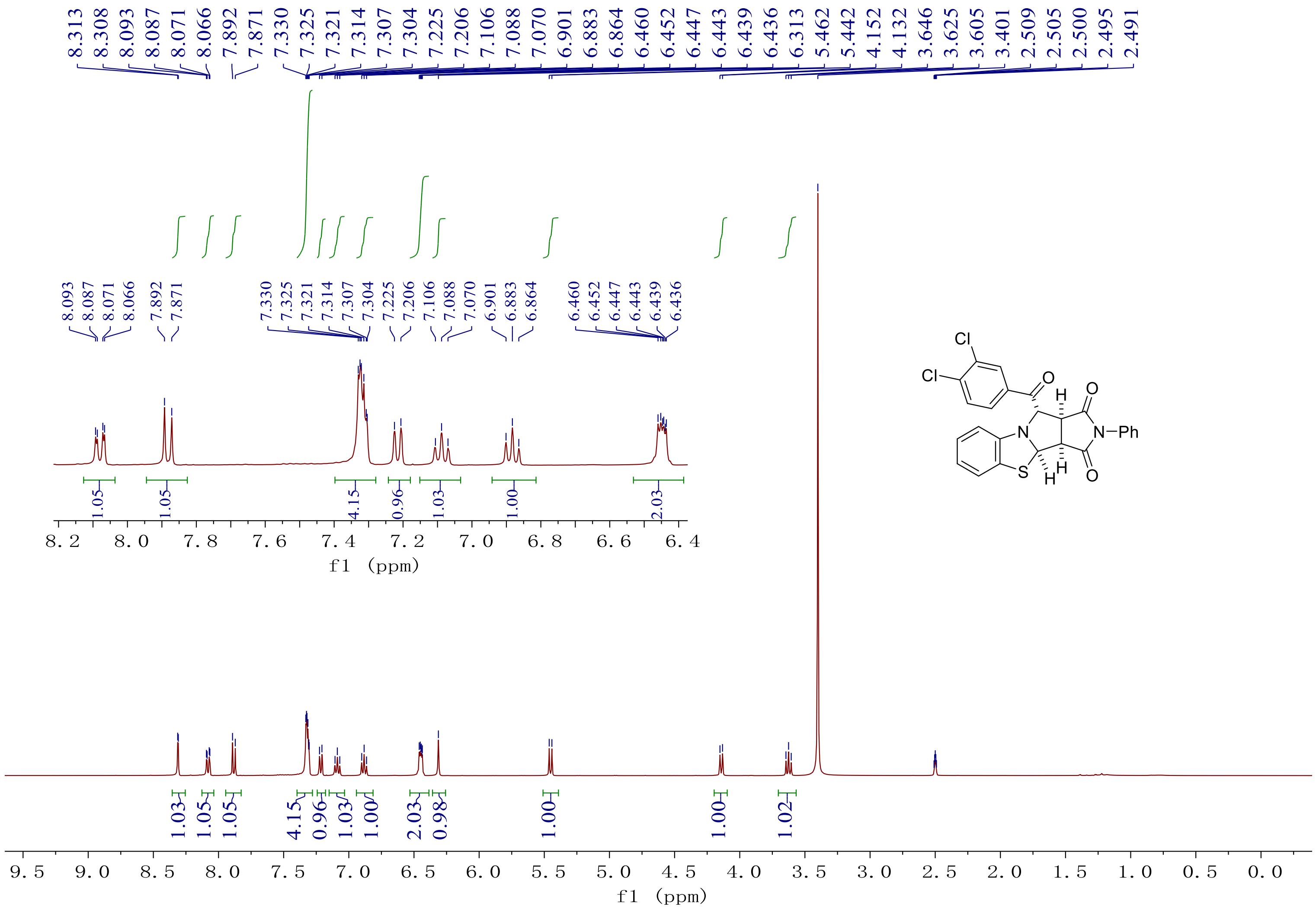

^{13}C NMR (101 MHz, $CDCl_3$) of **3ia**

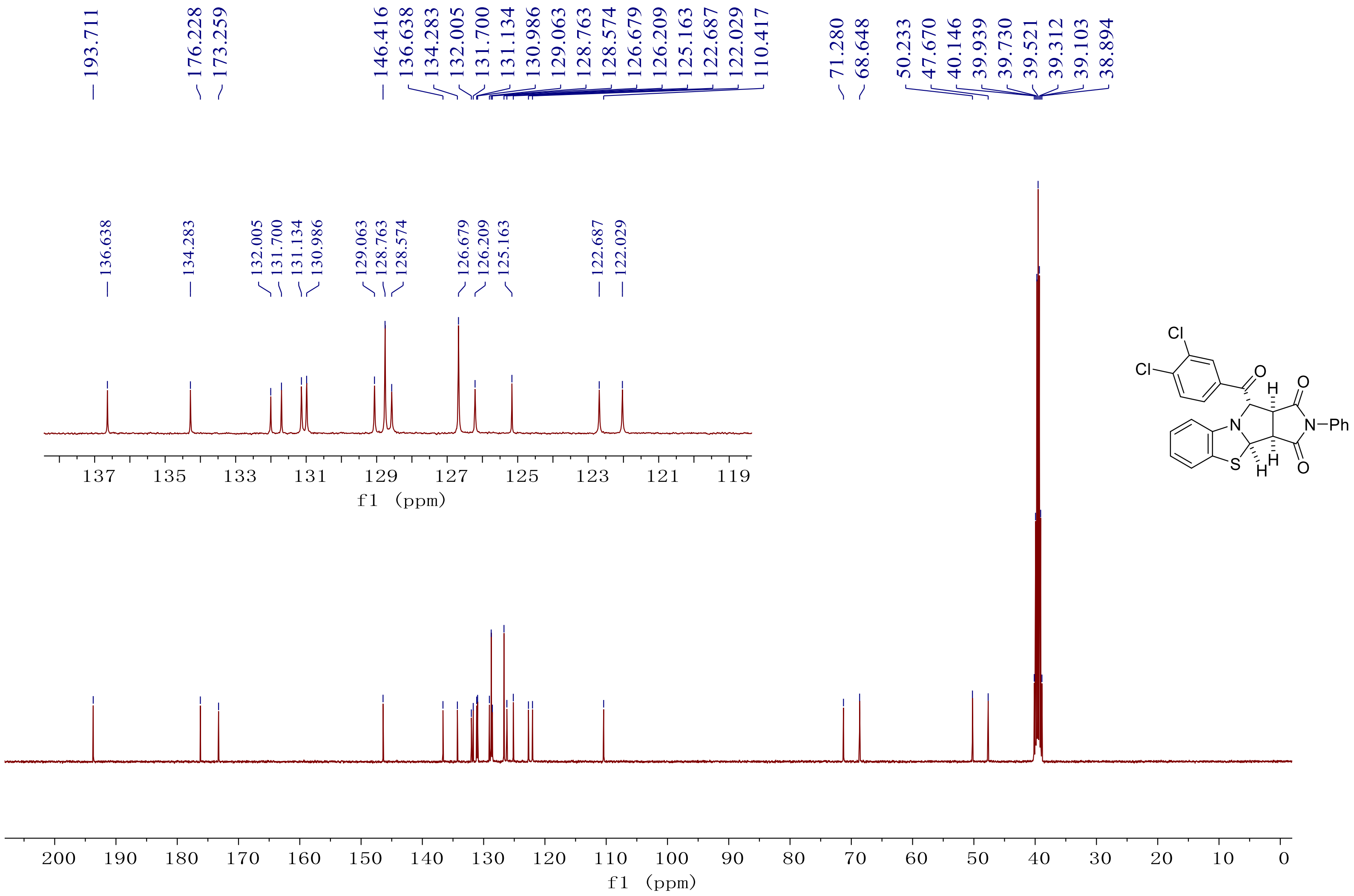

^1H NMR (400 MHz, $CDCl_3$) of **3ja**

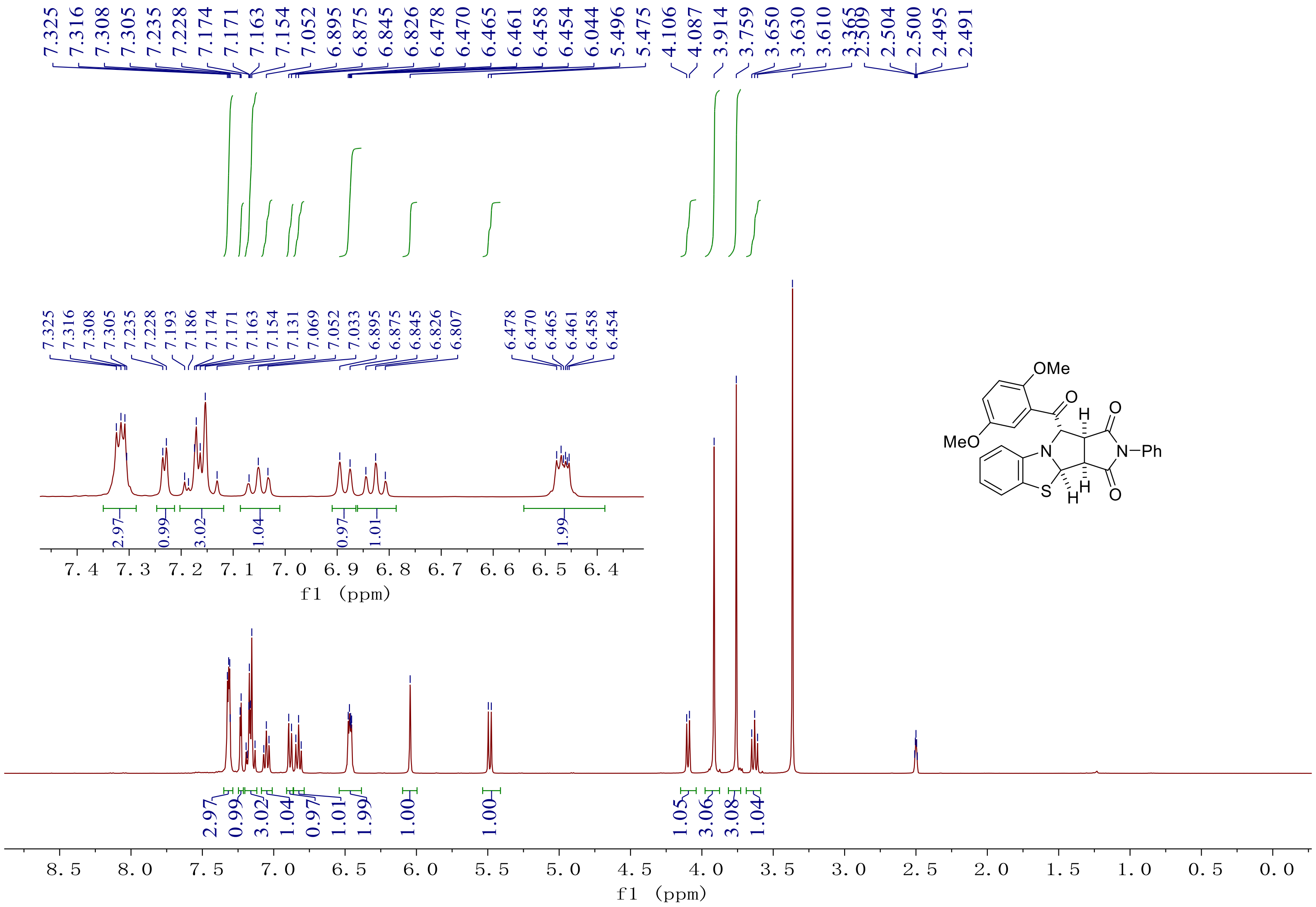

^{13}C NMR (101 MHz, $CDCl_3$) of **3ja**

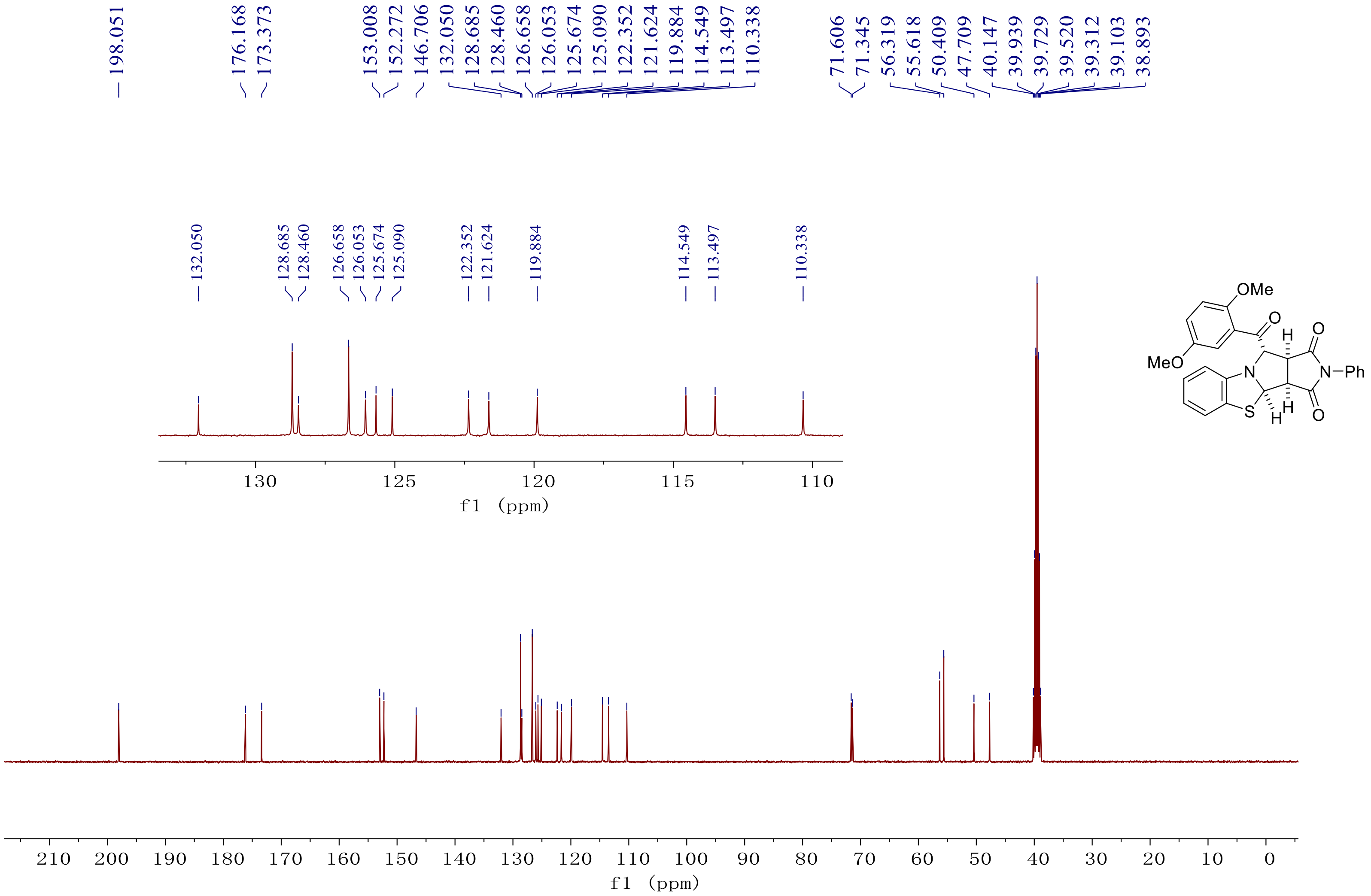

^{1}H NMR (400 MHz, $CDCl_3$) of **3ka**

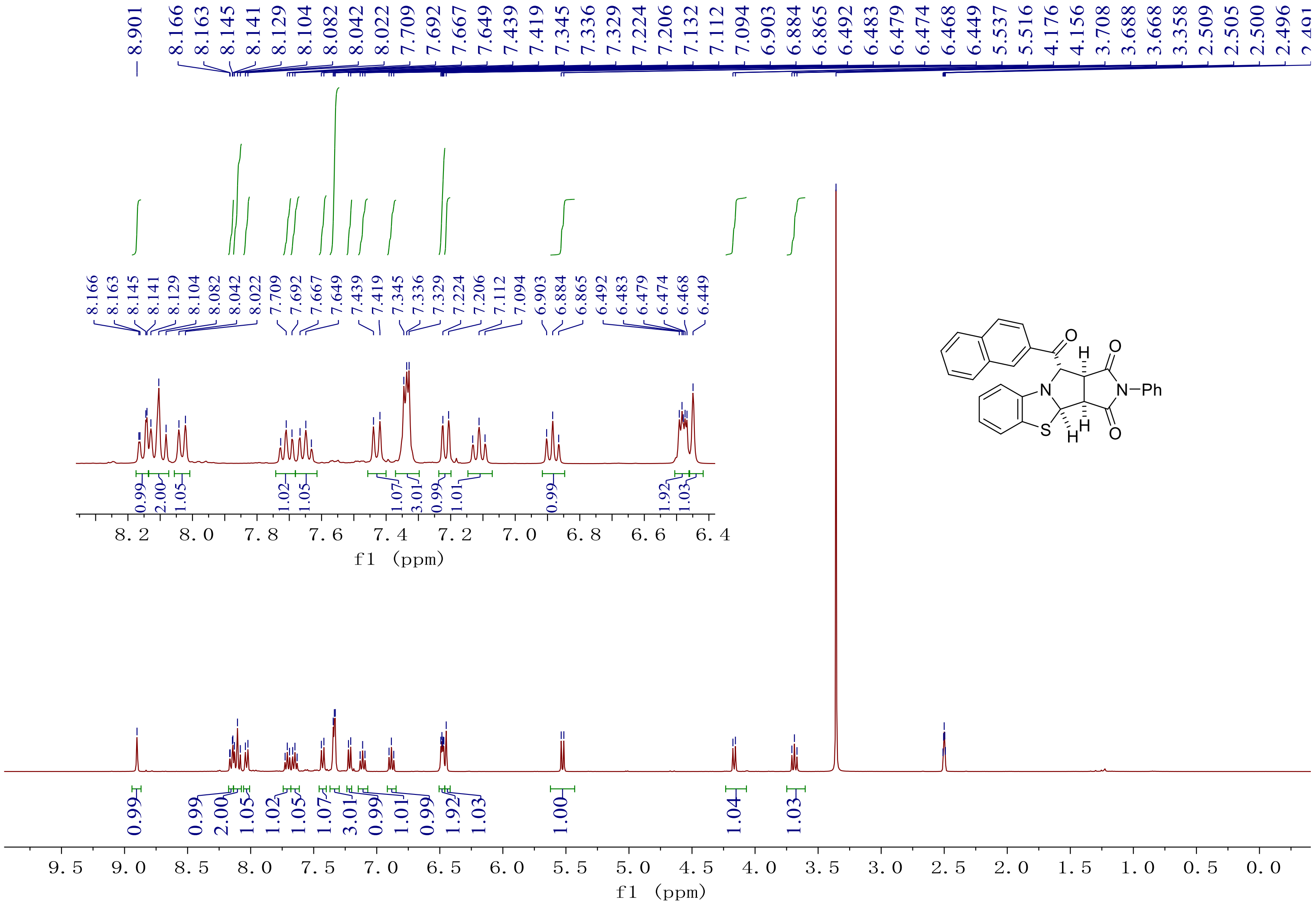

^{13}C NMR (101 MHz, CDCl$_3$) of **3ka**

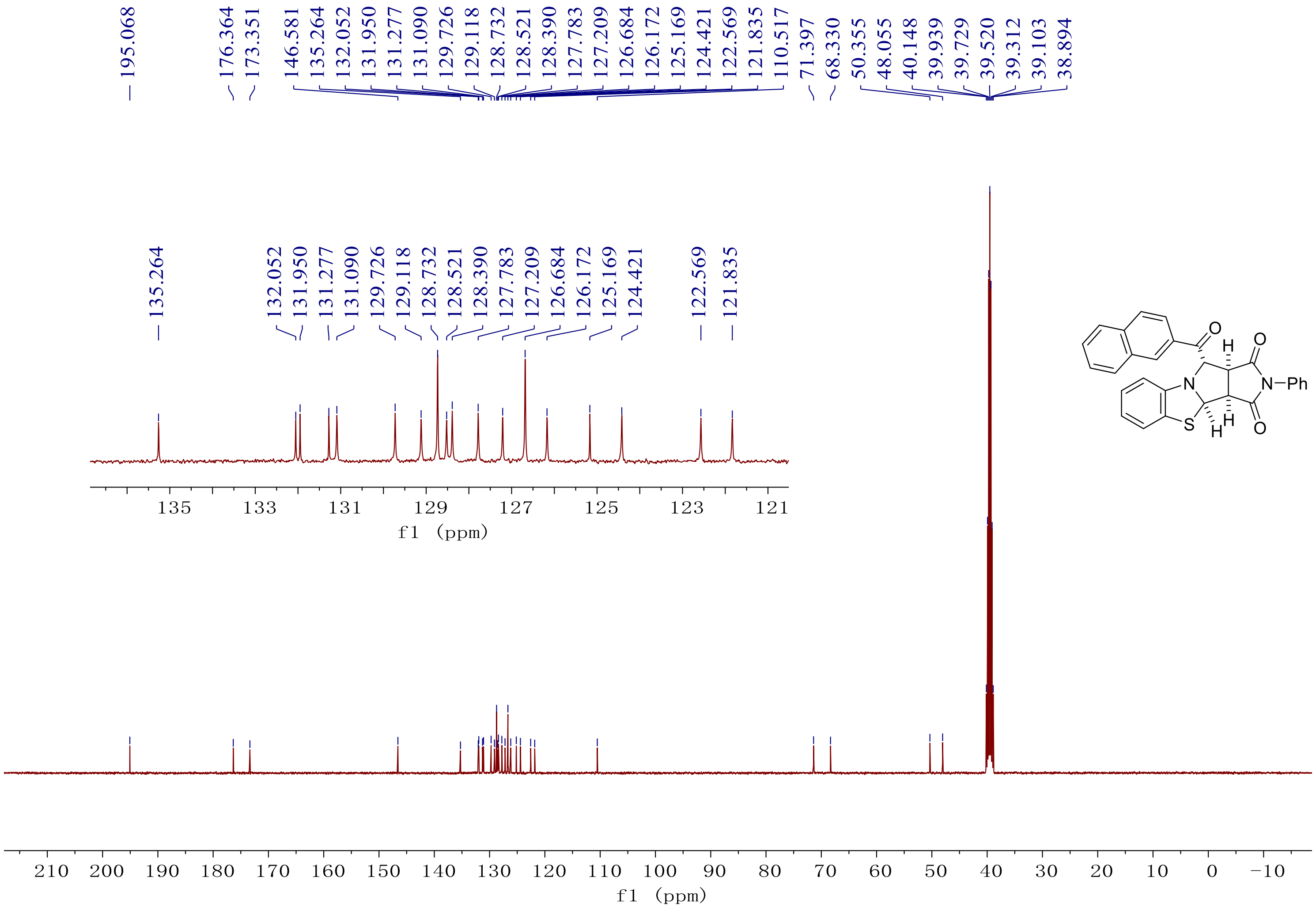

^{1}H NMR (400 MHz, $CDCl_3$) of **3la**

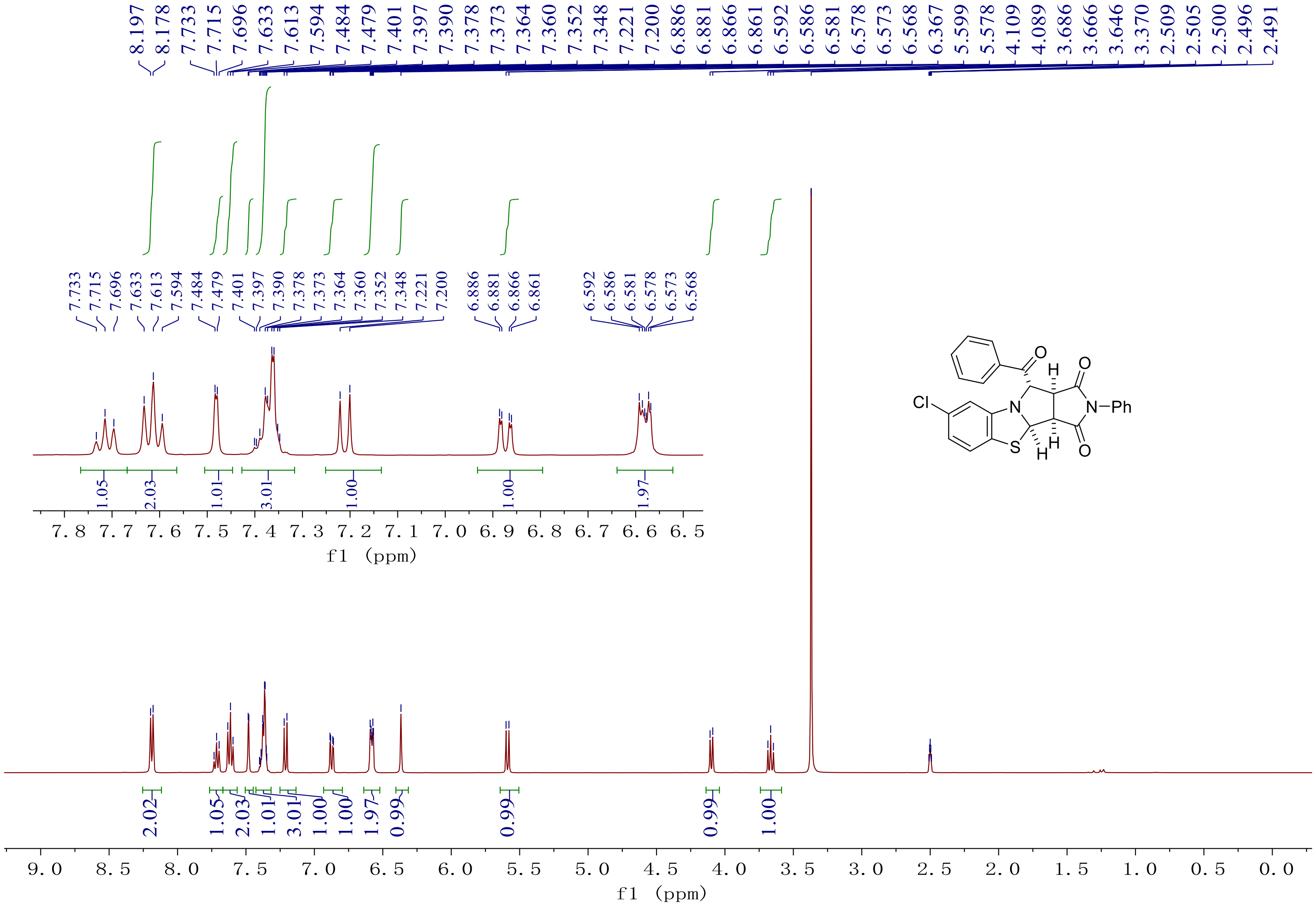

^{13}C NMR (101 MHz, $CDCl_3$) of **3la**

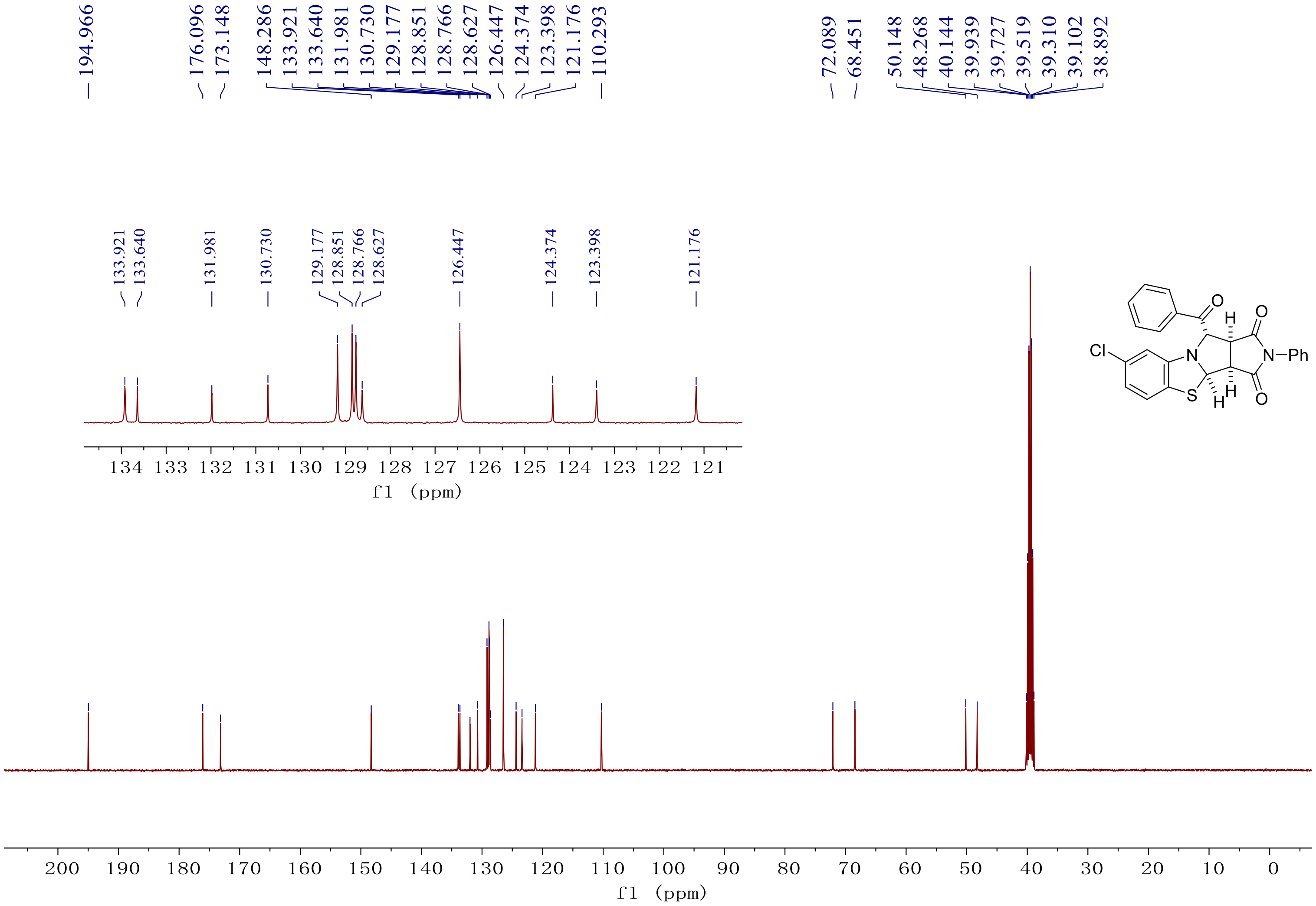

^{1}H NMR (400 MHz, $CDCl_3$) of **3ab**

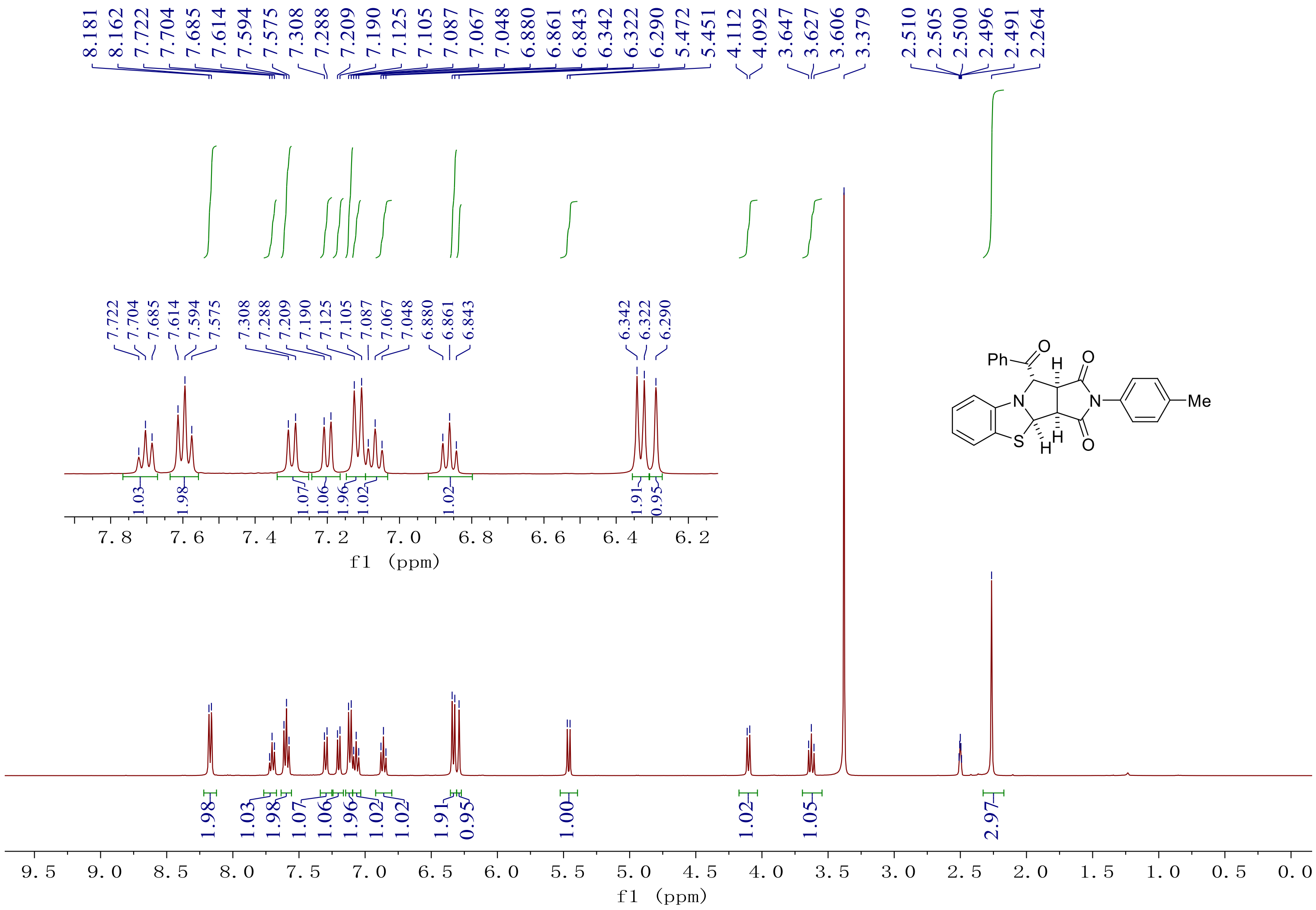

^{13}C NMR (101 MHz, $CDCl_3$) of **3ab**

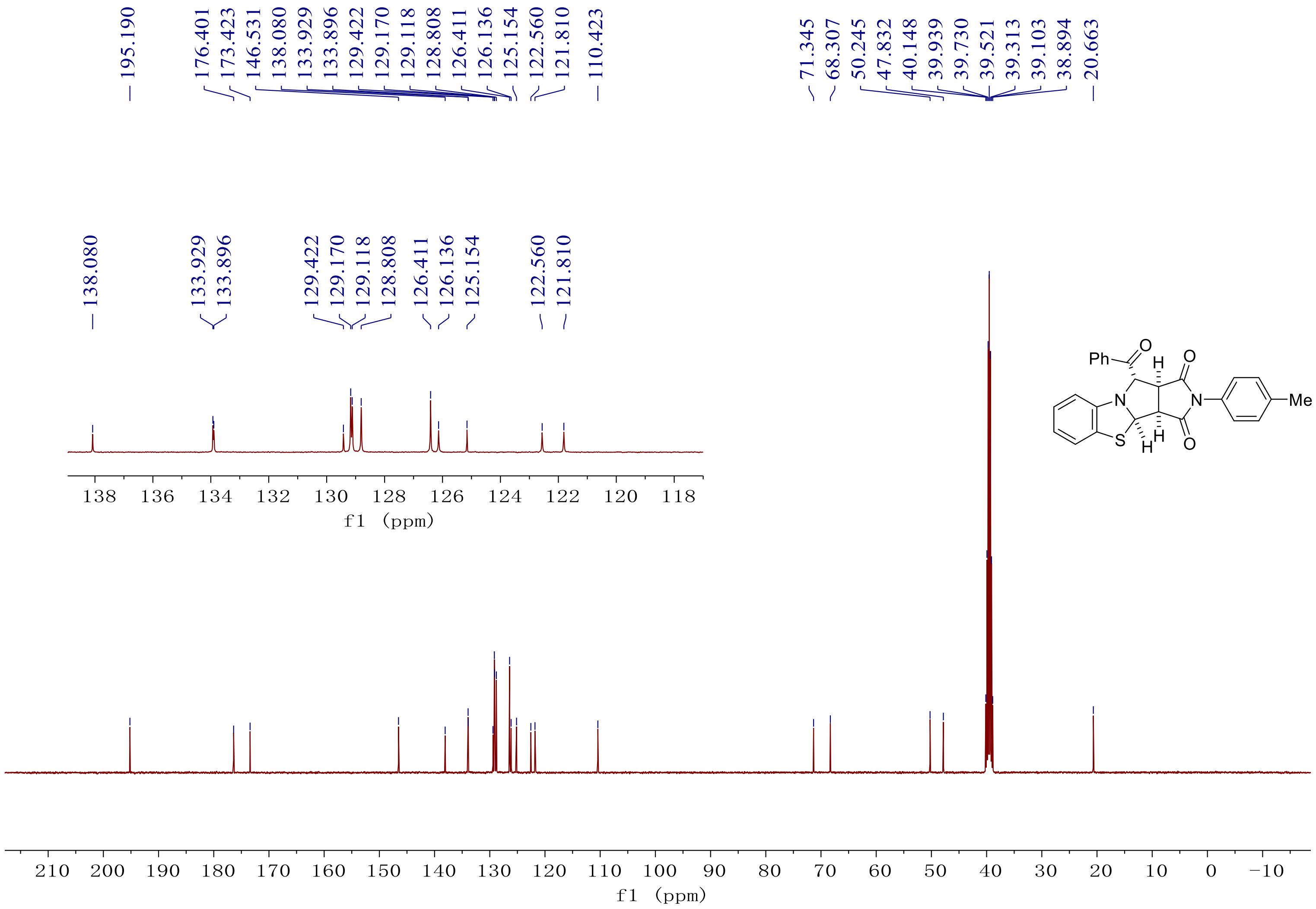

^{1}H NMR (400 MHz, $CDCl_3$) of **3ac**

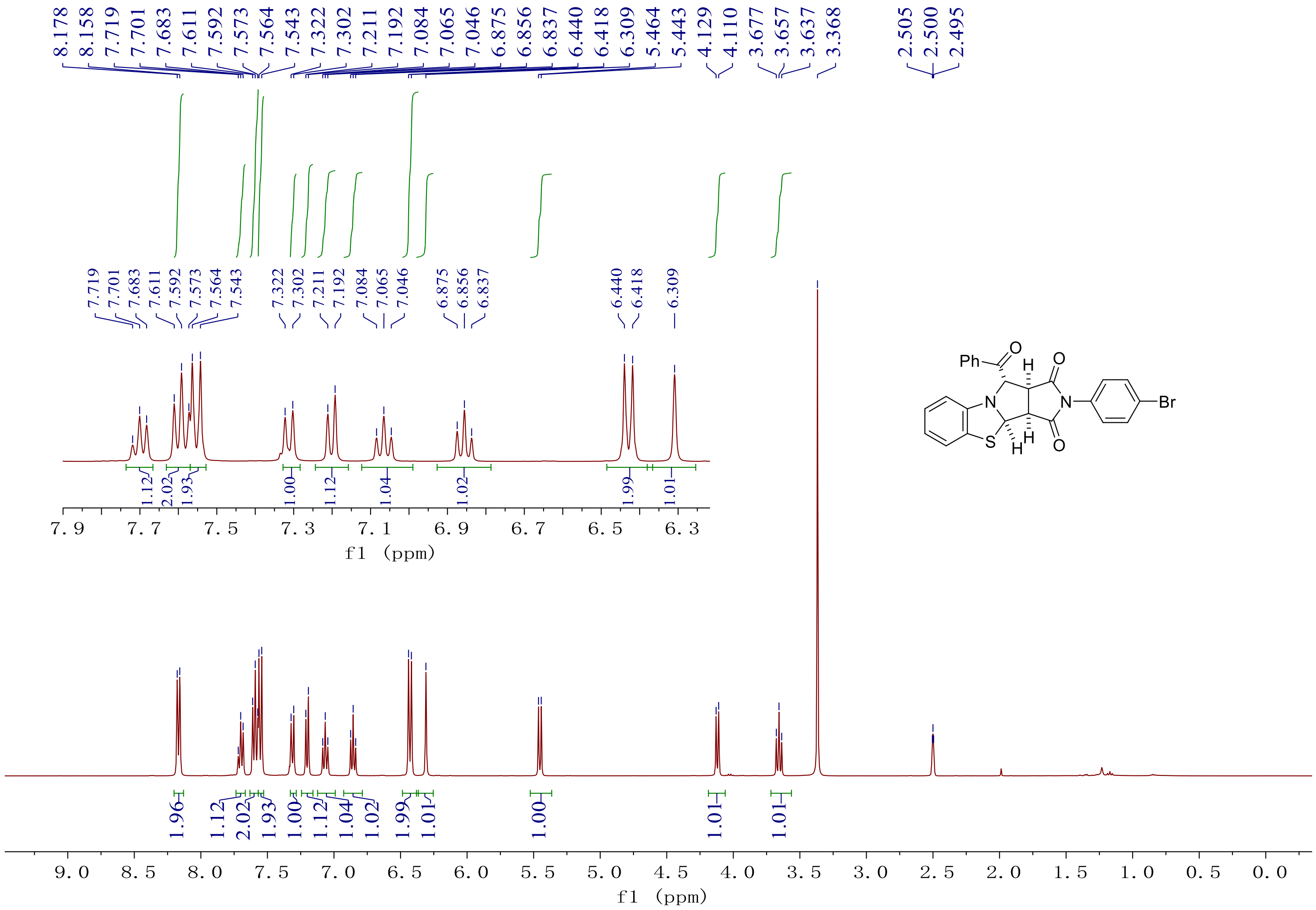

^{13}C NMR (101 MHz, $CDCl_3$) of **3ac**

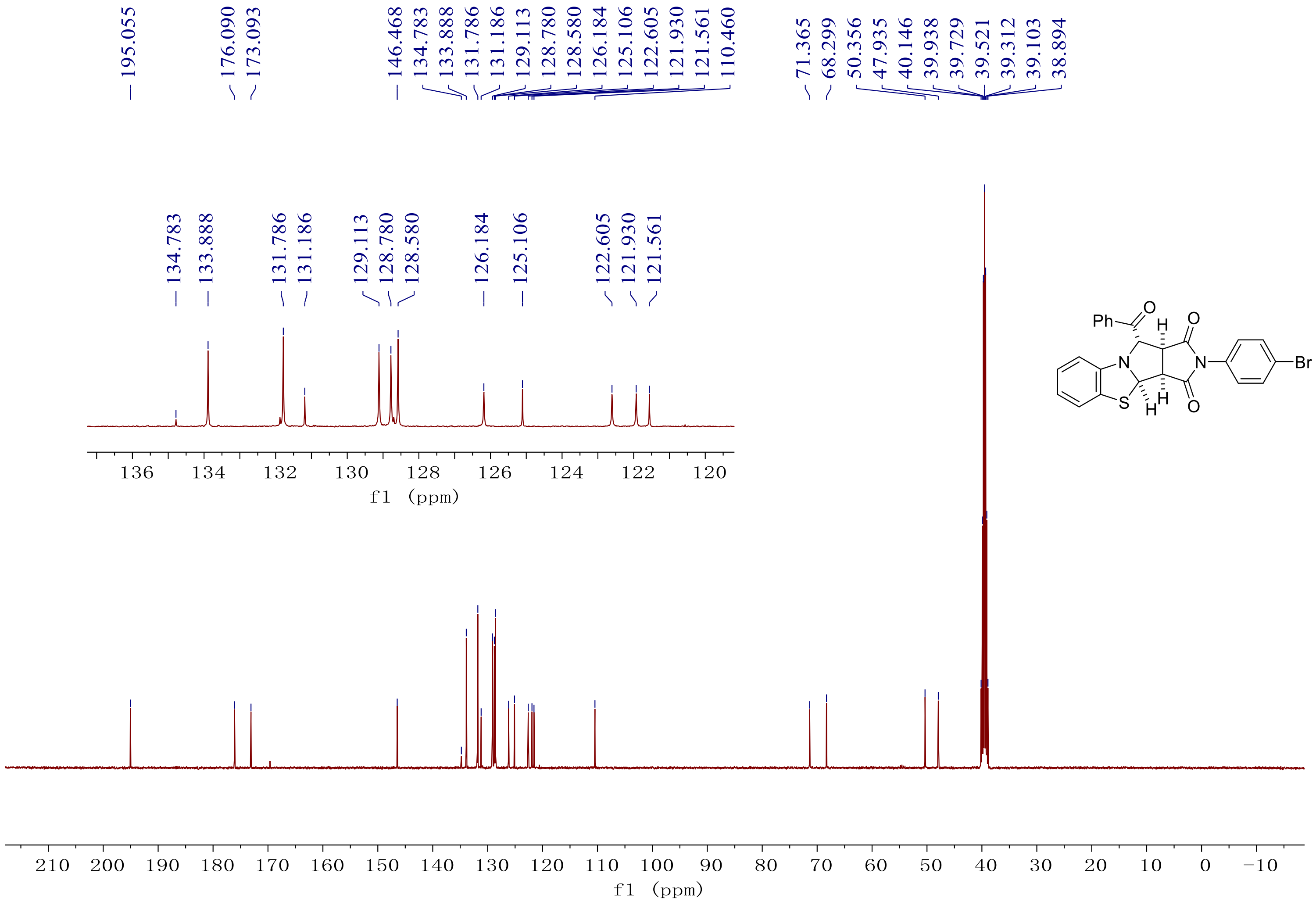

^{1}H NMR (400 MHz, $CDCl_3$) of **3ad**

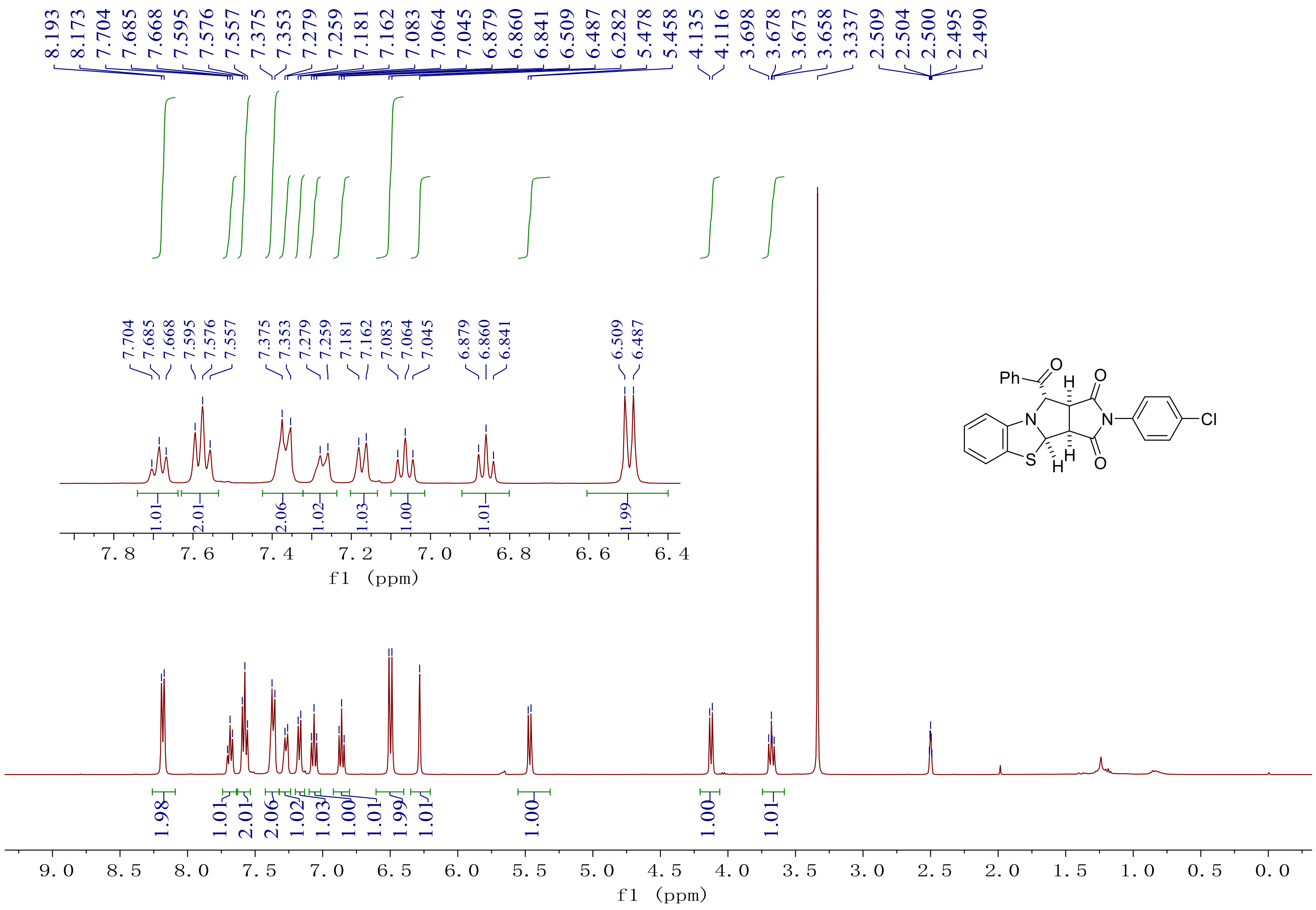

^{13}C NMR (101 MHz, $CDCl_3$) of **3ad**

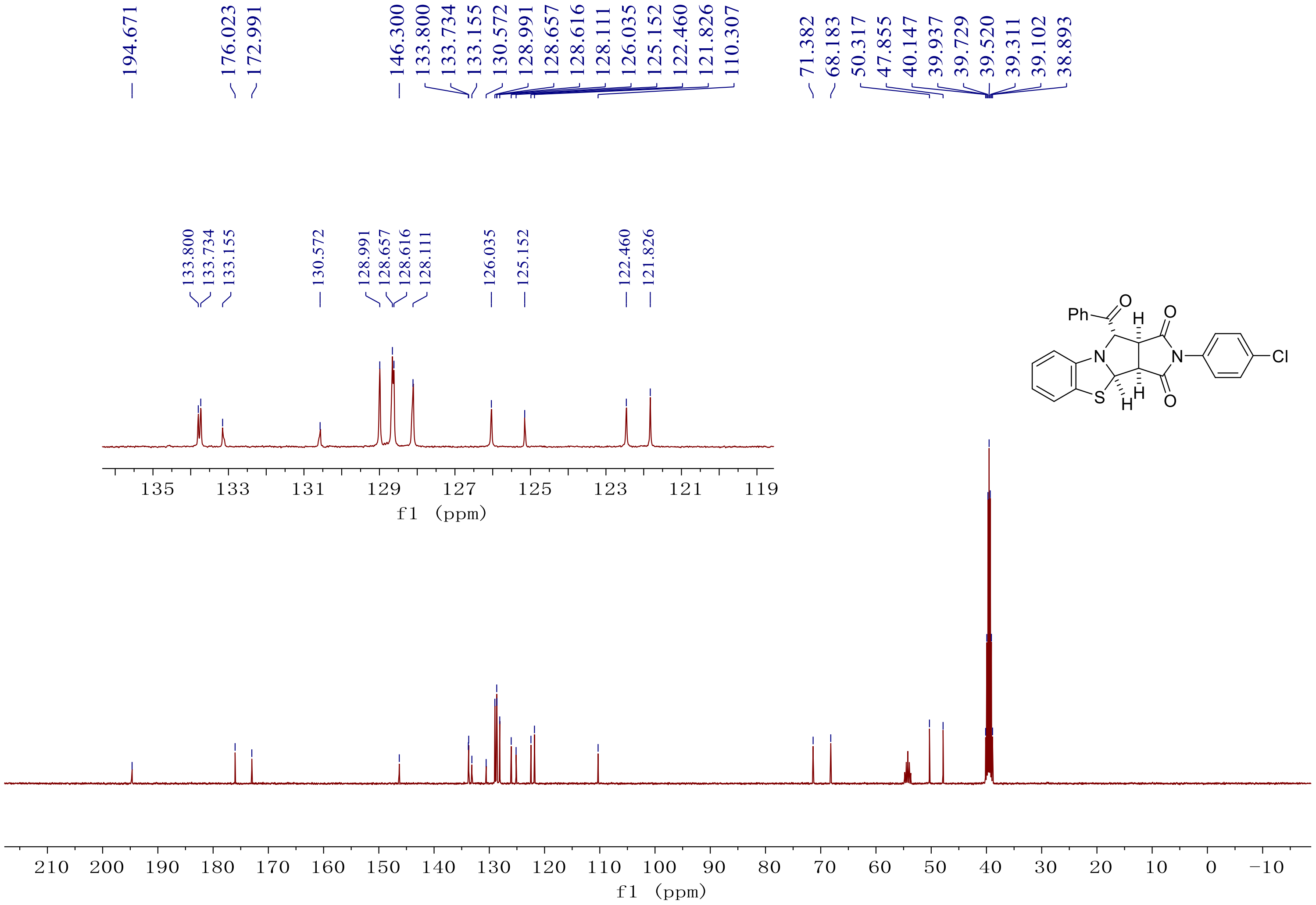

^{1}H NMR (400 MHz, $CDCl_3$) of **3ae**

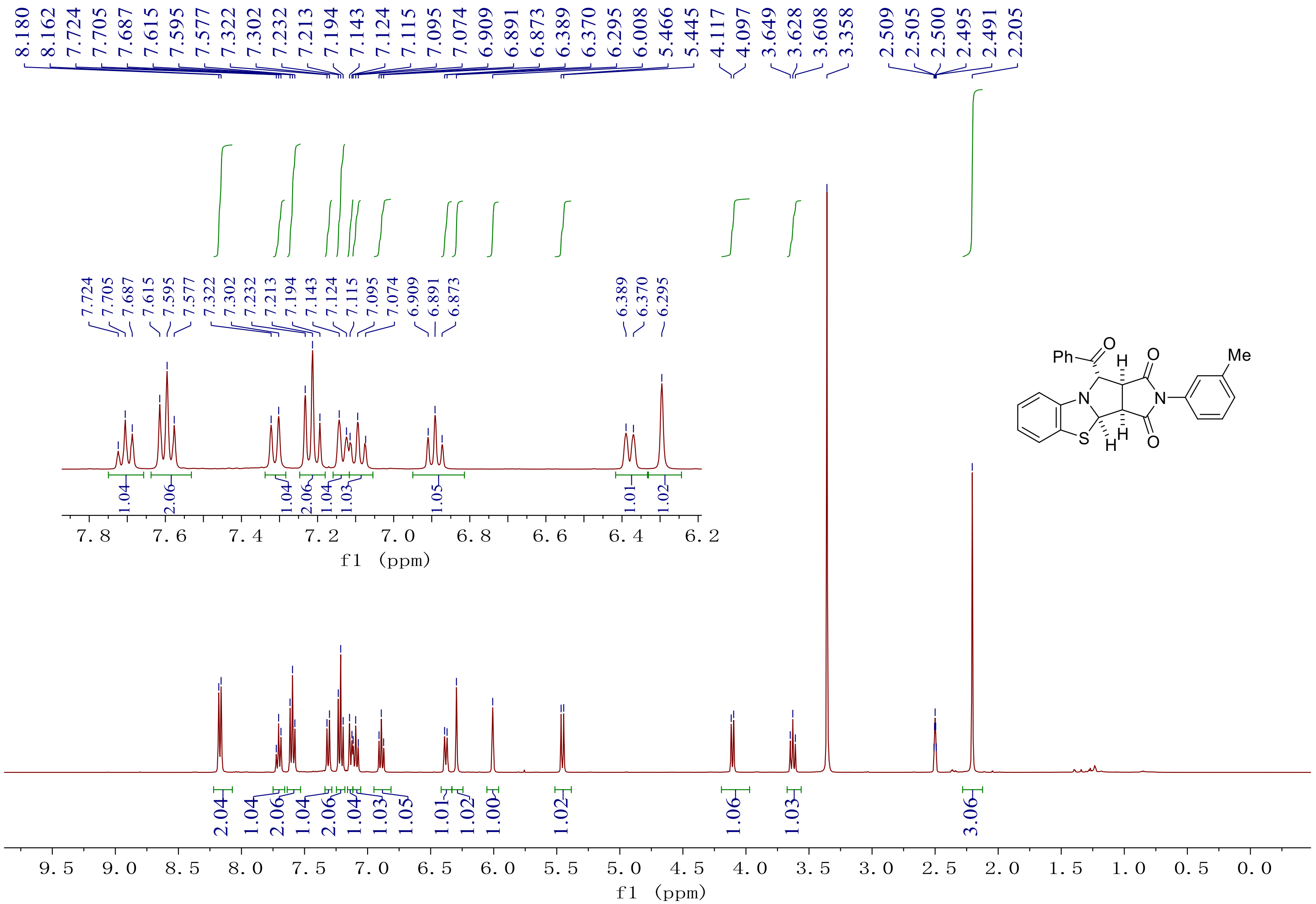

^{13}C NMR (101 MHz, $CDCl_3$) of **3ae**

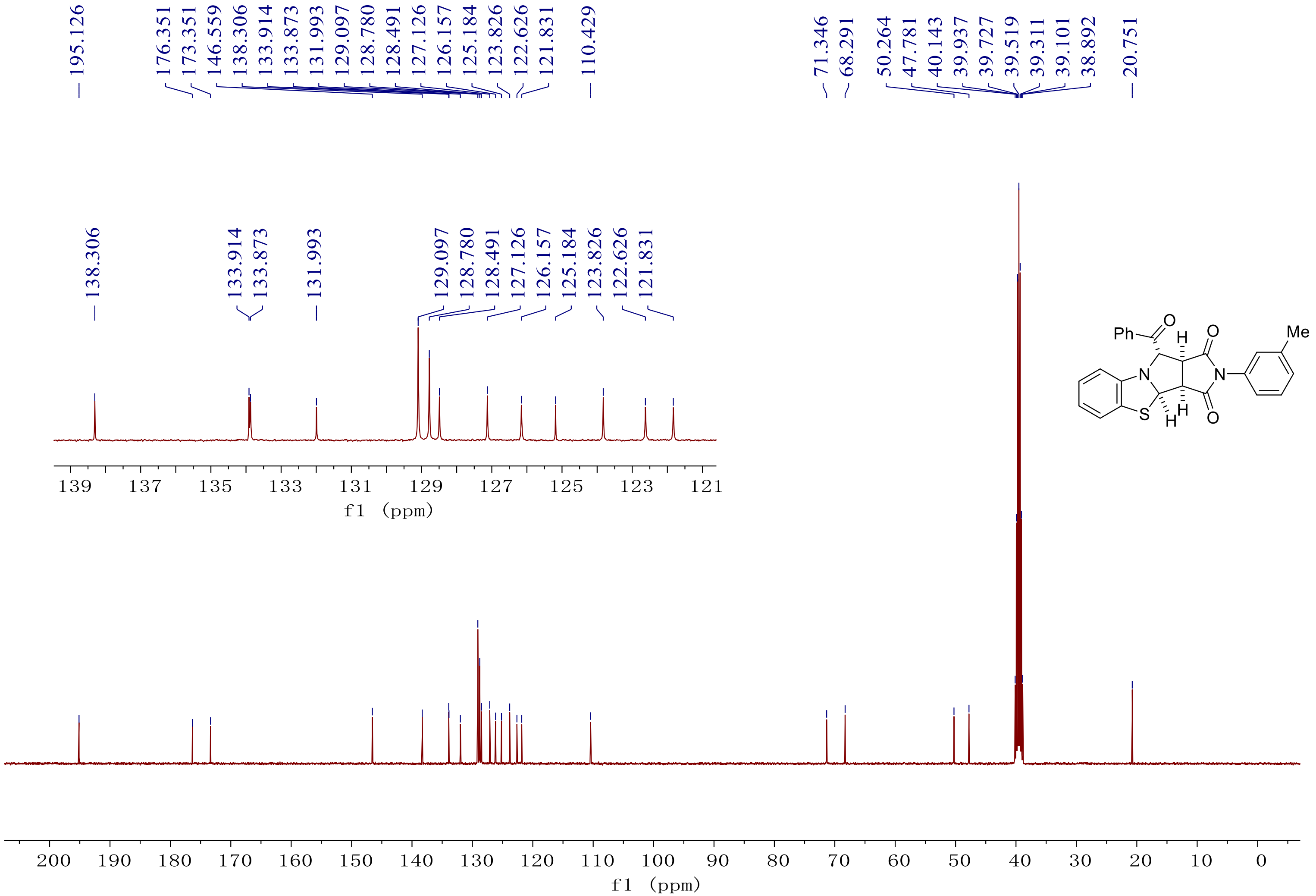

^{1}H NMR (400 MHz, CDCl3) of **3af**

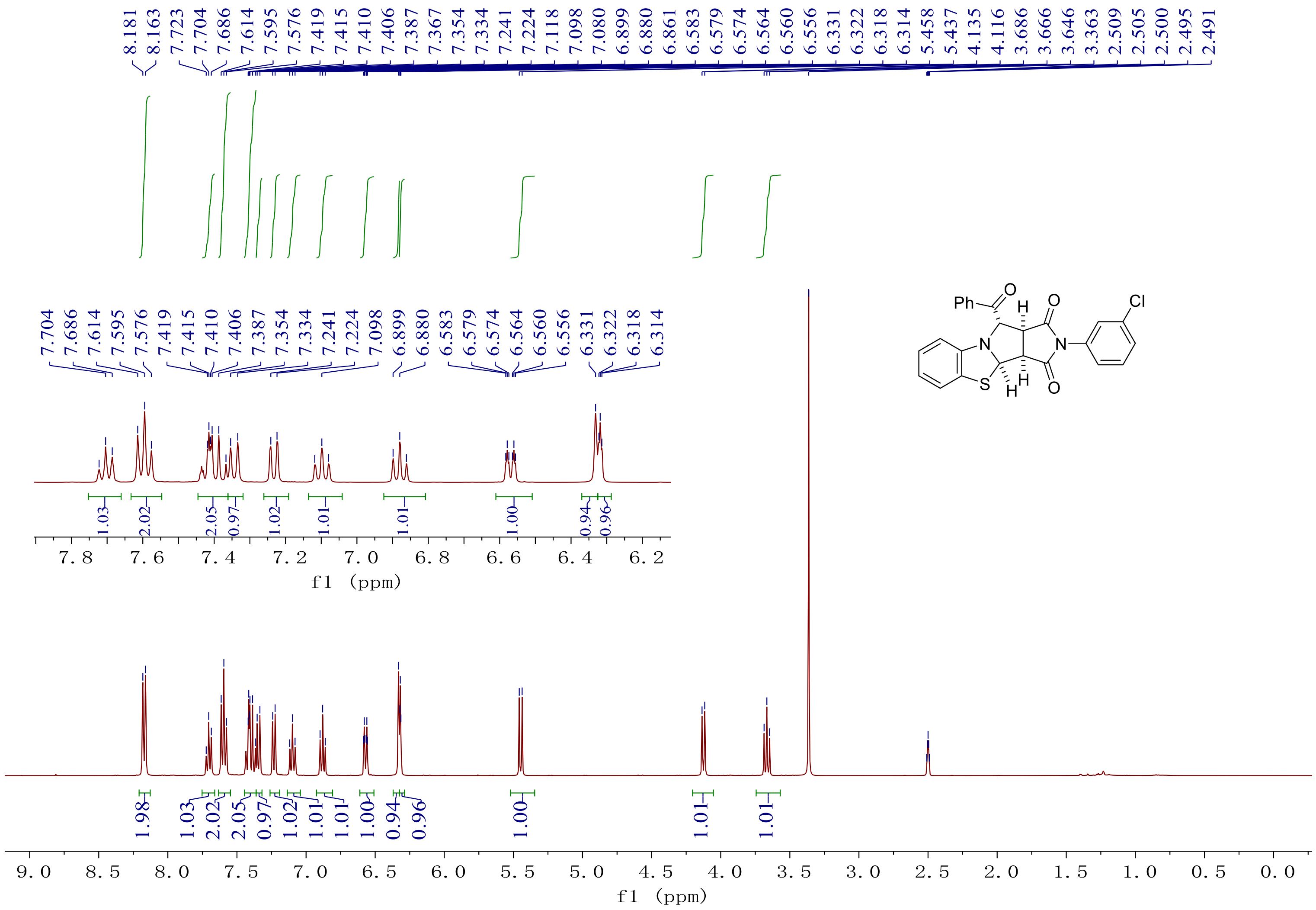

^{13}C NMR (101 MHz, $CDCl_3$) of **3af**

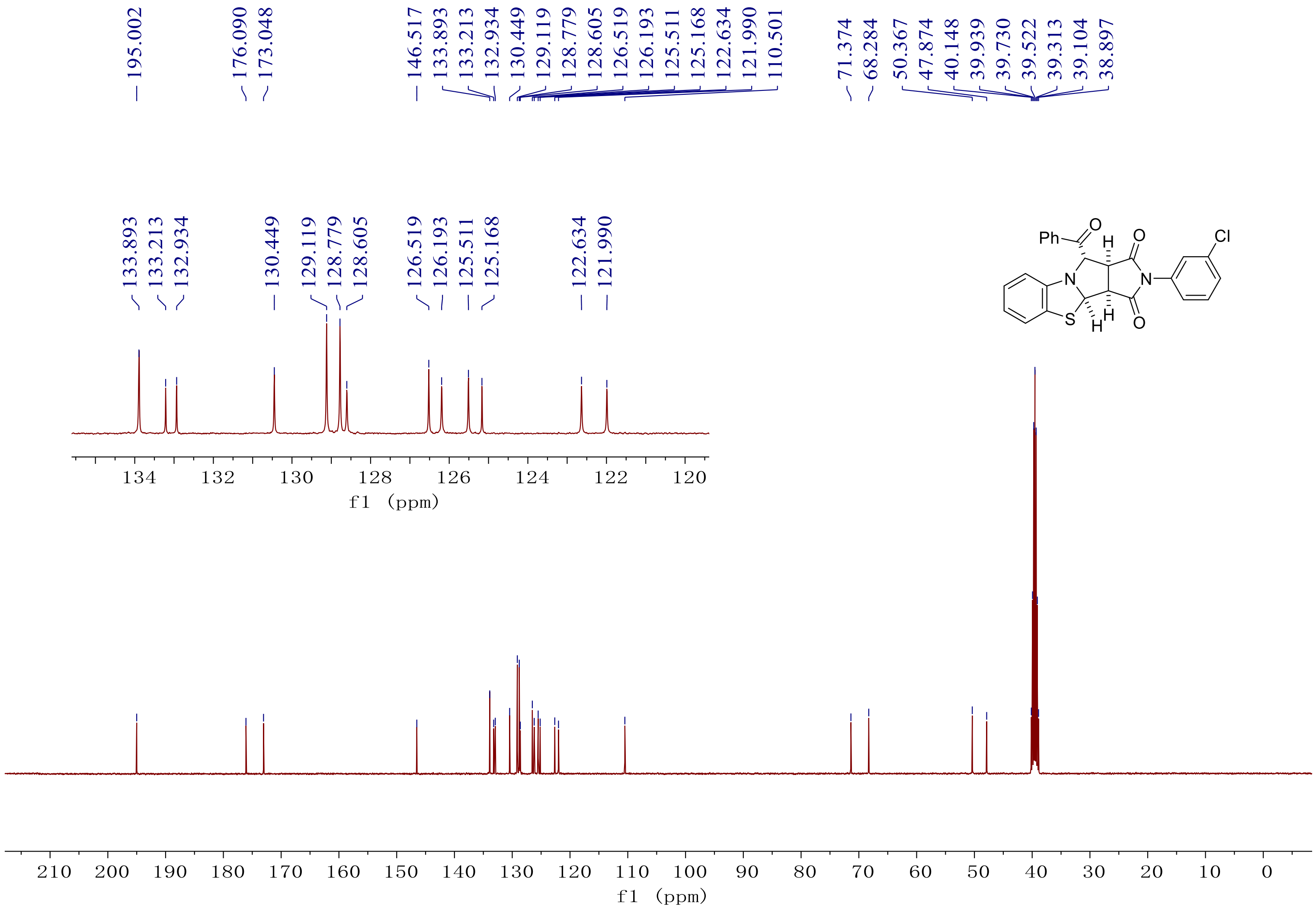

^{1}H NMR (400 MHz, $CDCl_3$) of **3ag**

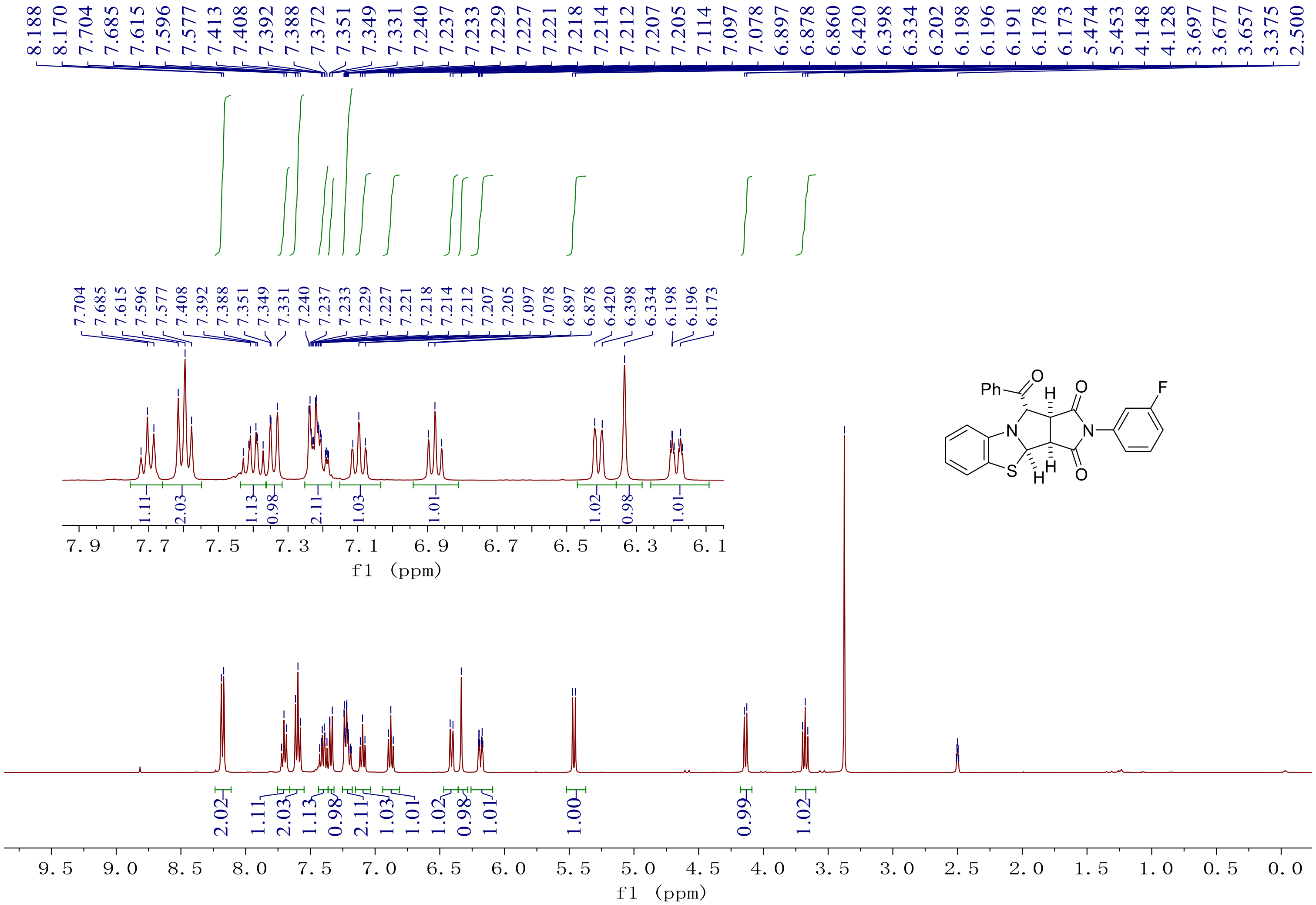

^{13}C NMR (101 MHz, $CDCl_3$) of **3ag**

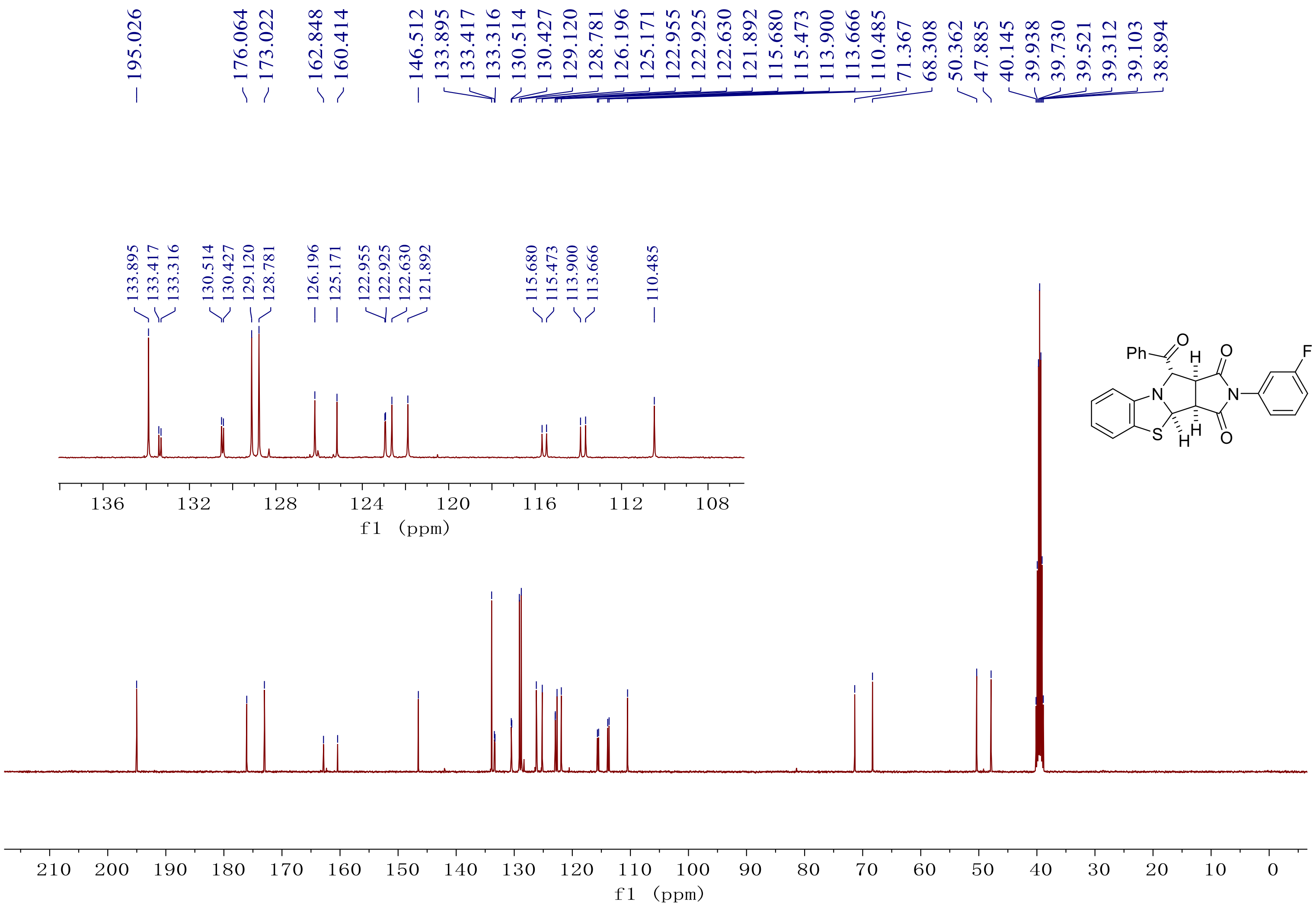

^{1}H NMR (400 MHz, $CDCl_3$) of **3ah**

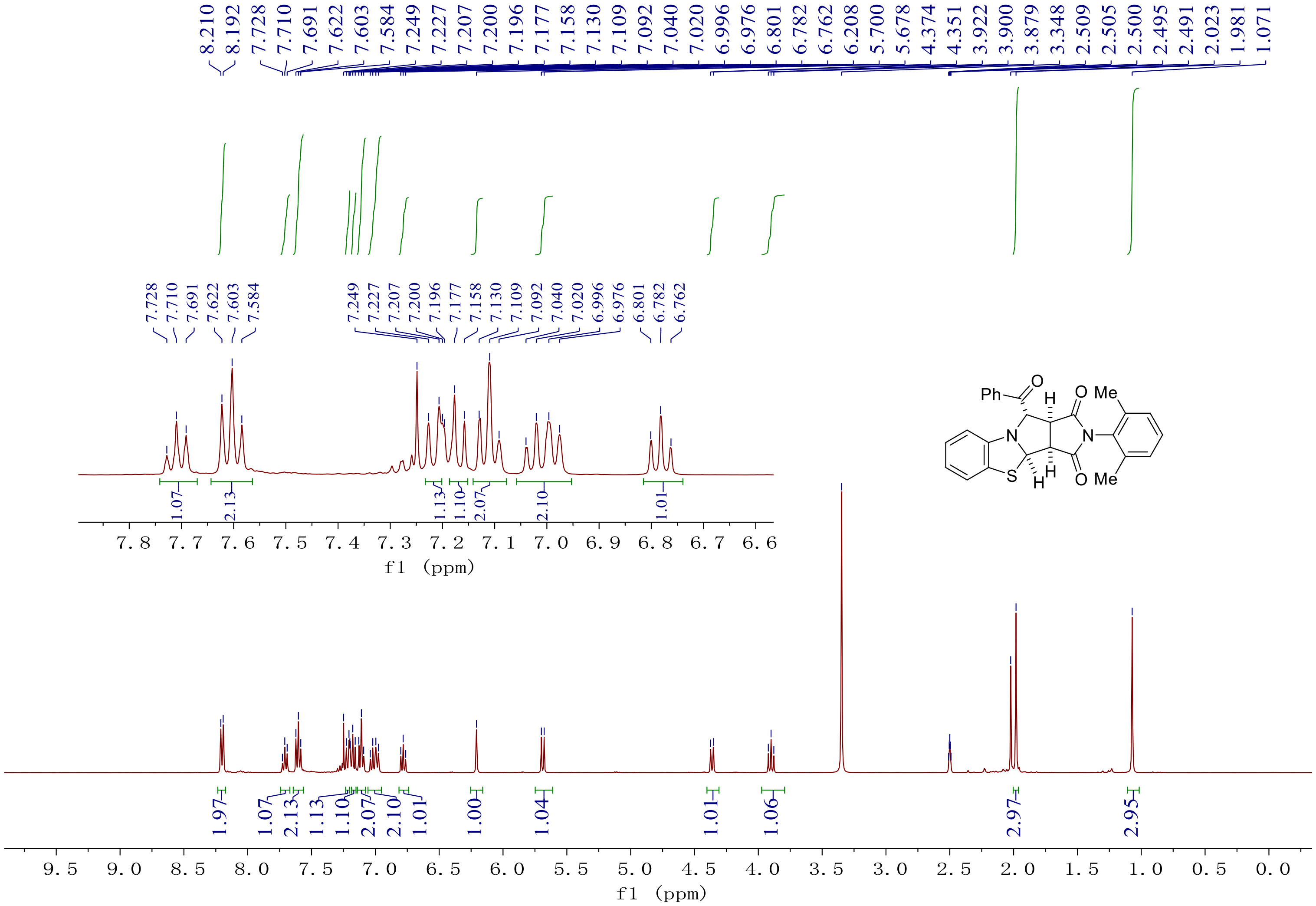

^{13}C NMR (101 MHz, $CDCl_3$) of **3ah**

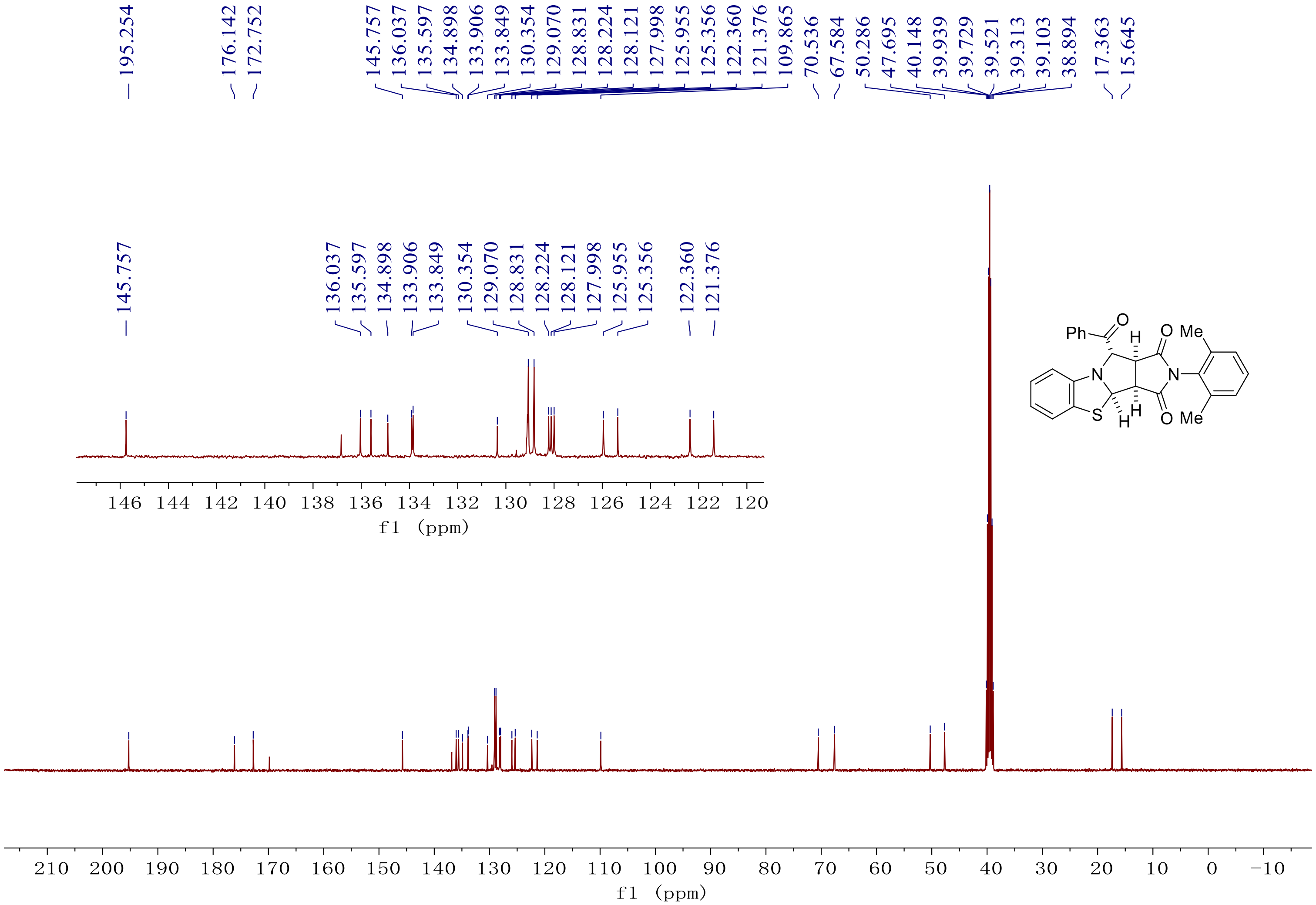

^{1}H NMR (400 MHz, $CDCl_3$) of **3ai**

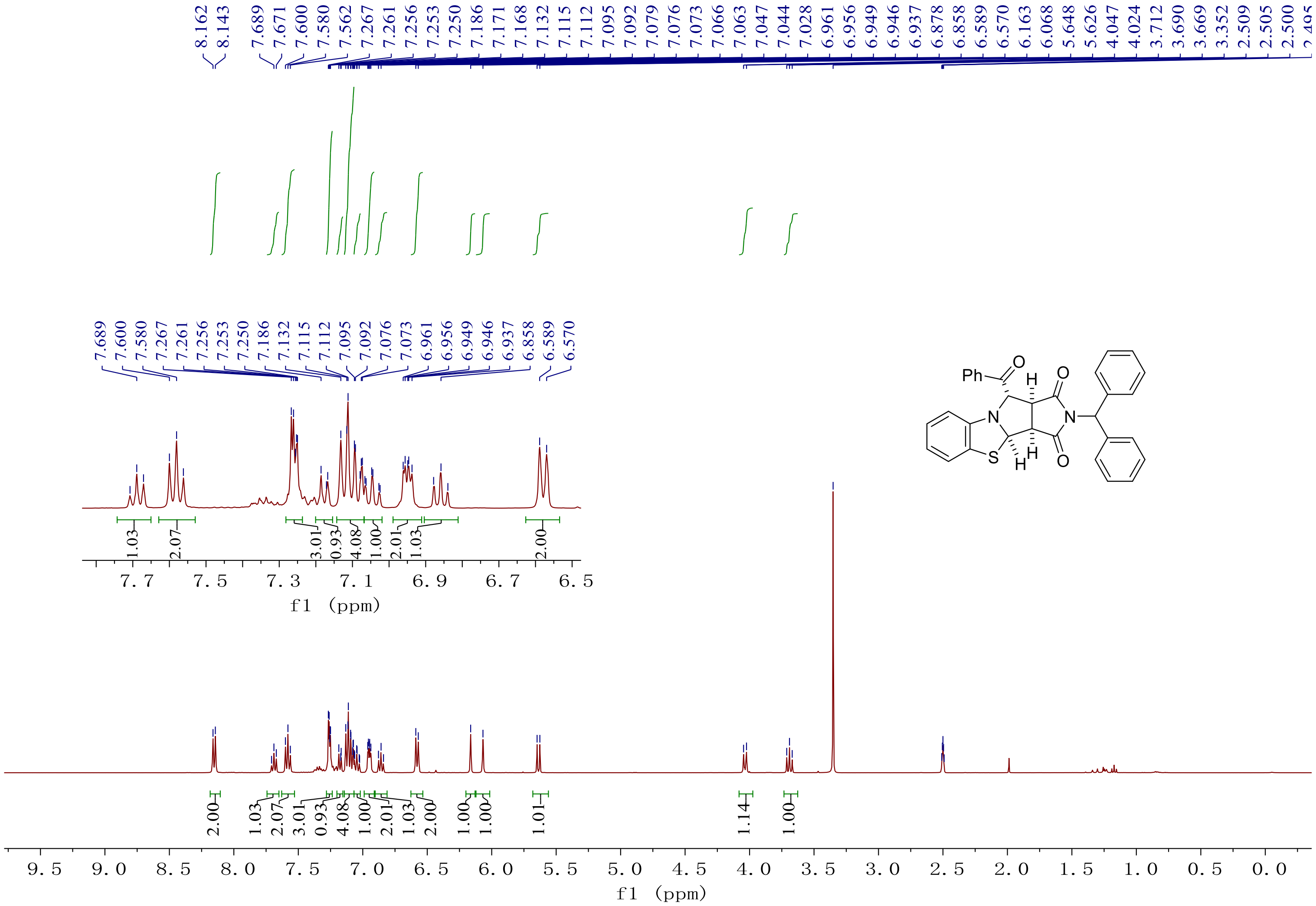

^{13}C NMR (101 MHz, $CDCl_3$) of **3ai**

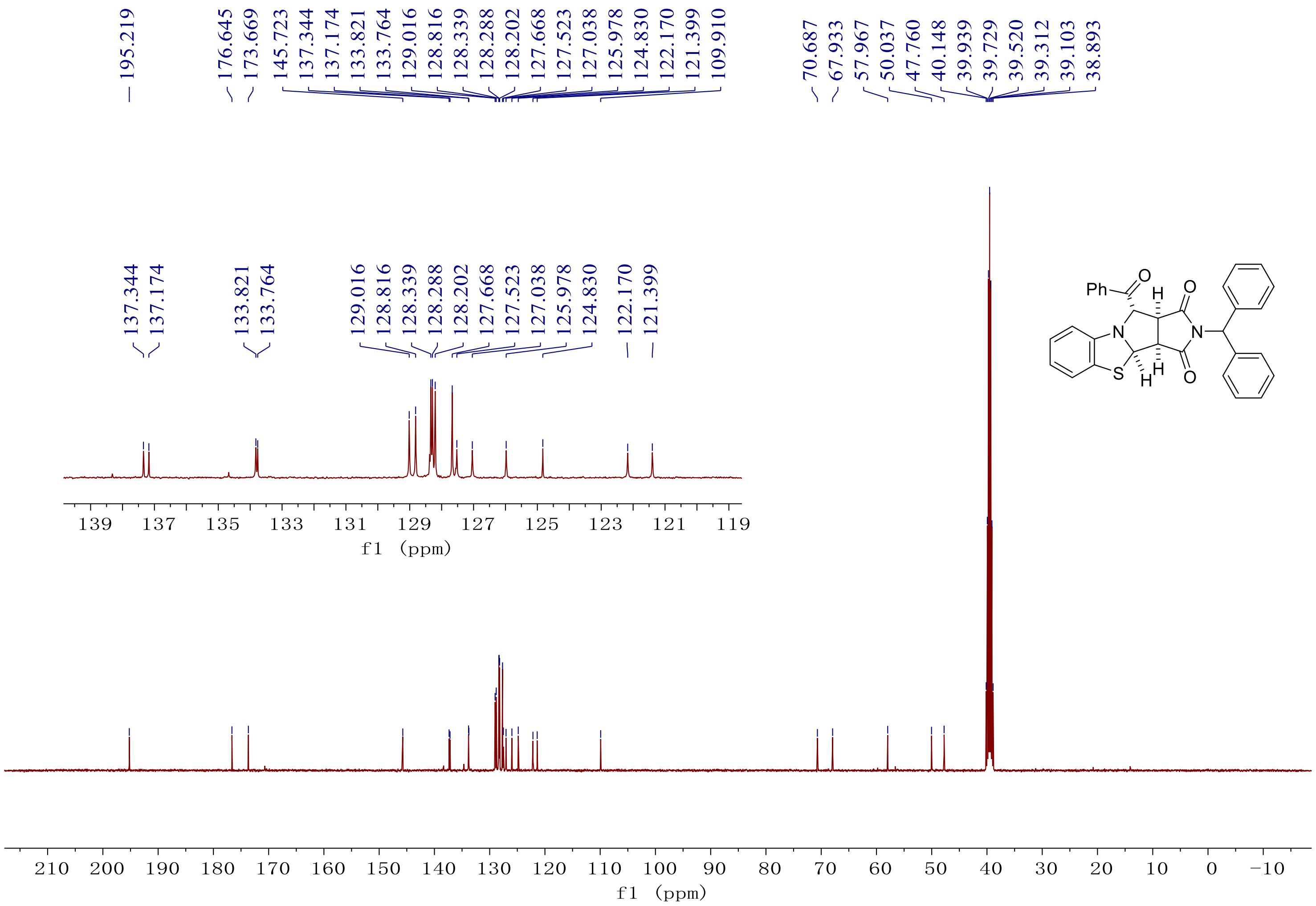

^{1}H NMR (400 MHz, $CDCl_3$) of **3aj**

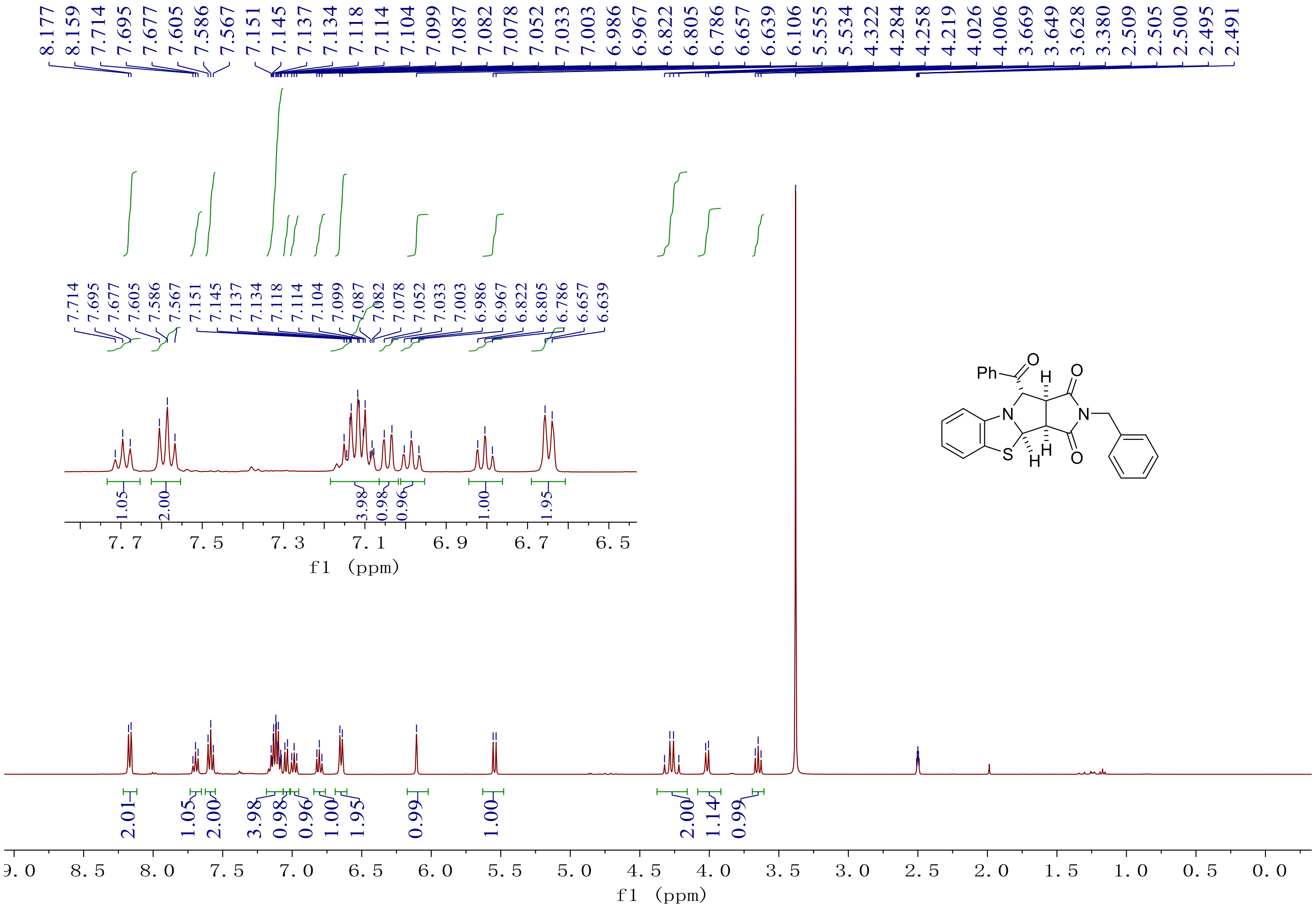

^{13}C NMR (101 MHz, $CDCl_3$) of **3aj**

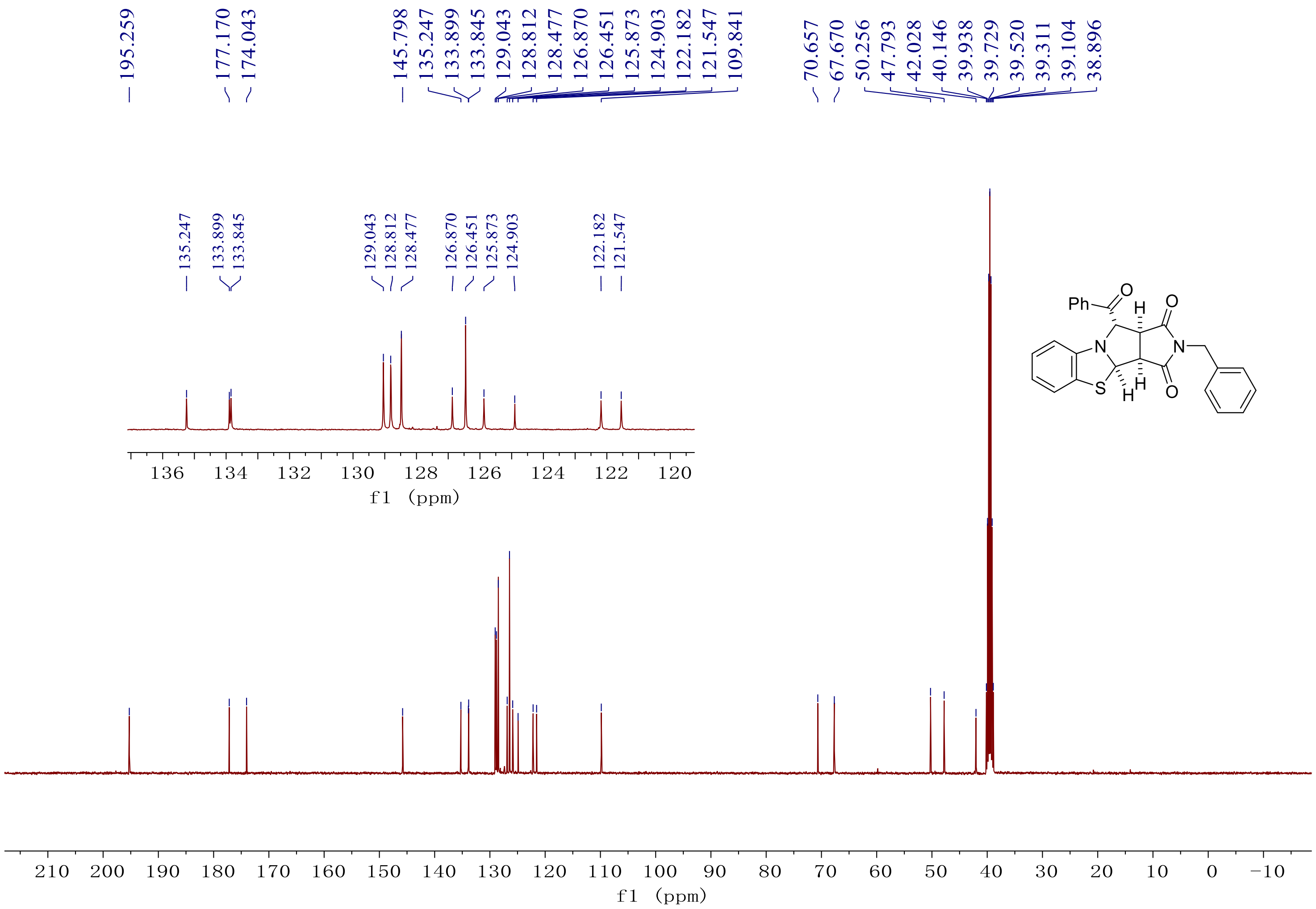

^{1}H NMR (400 MHz, $CDCl_3$) of **5aa**

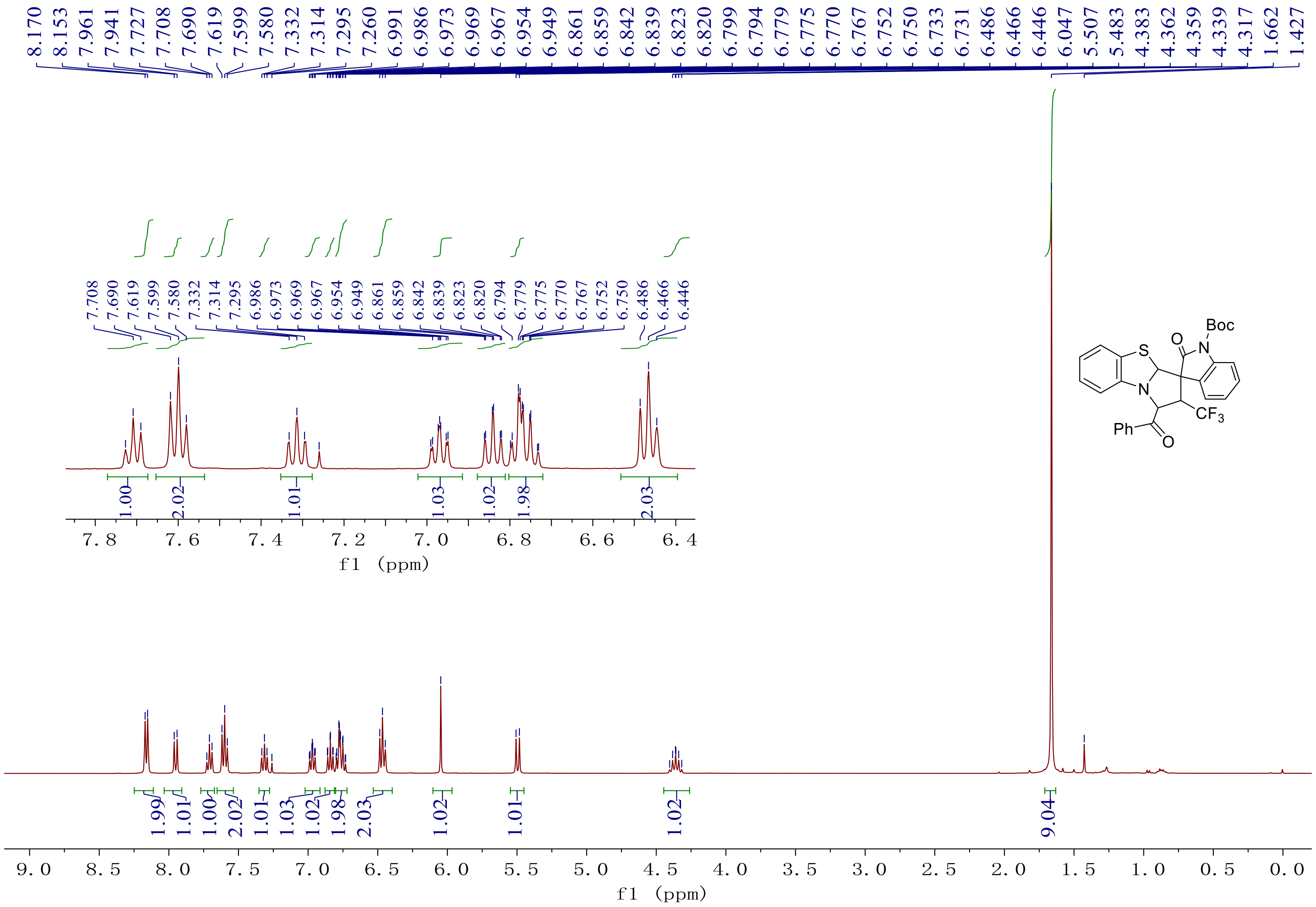

^{13}C NMR (101 MHz, $CDCl_3$) of **5aa**

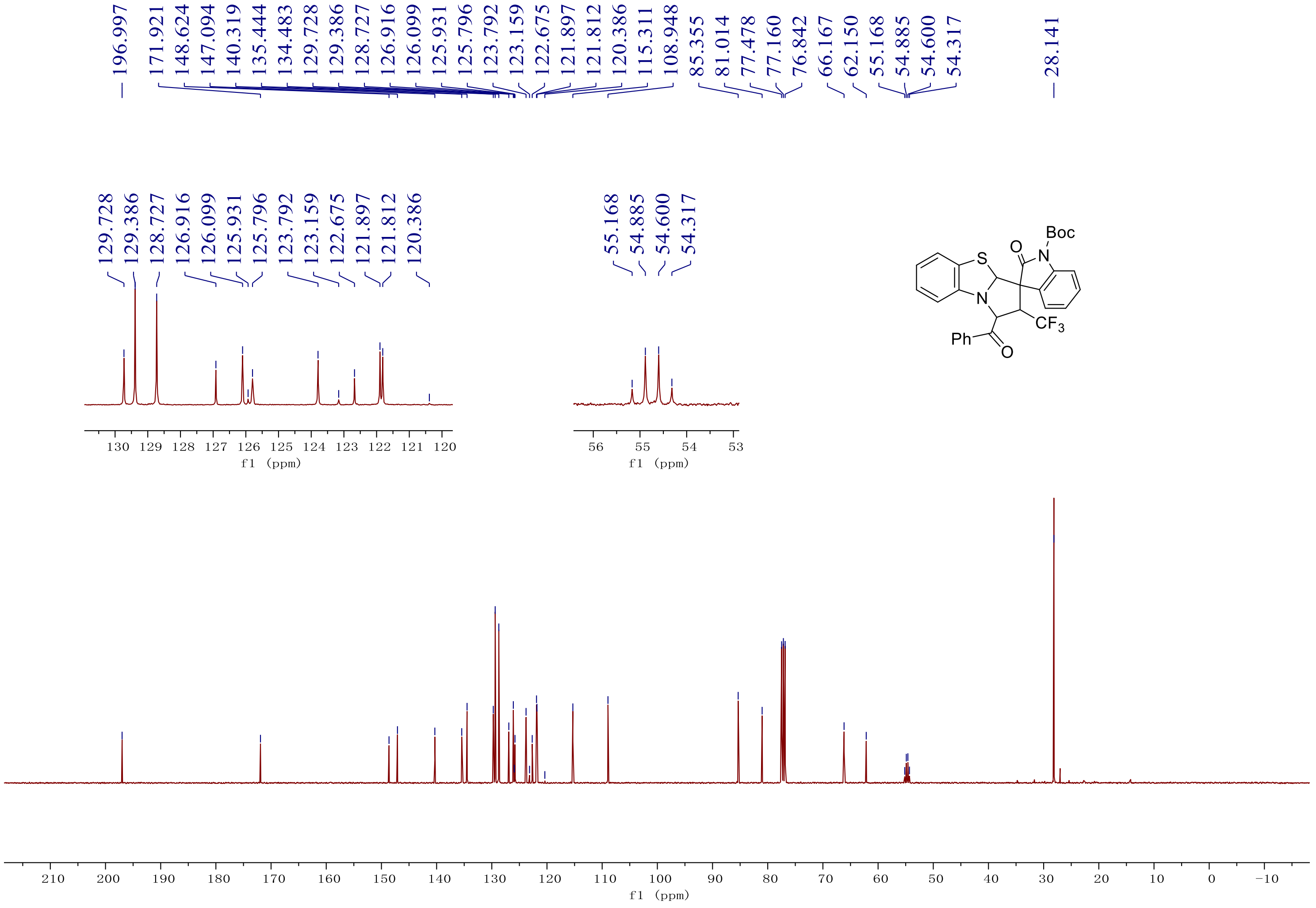

^{1}H NMR (300 MHz, $CDCl_3$) of **5ab**

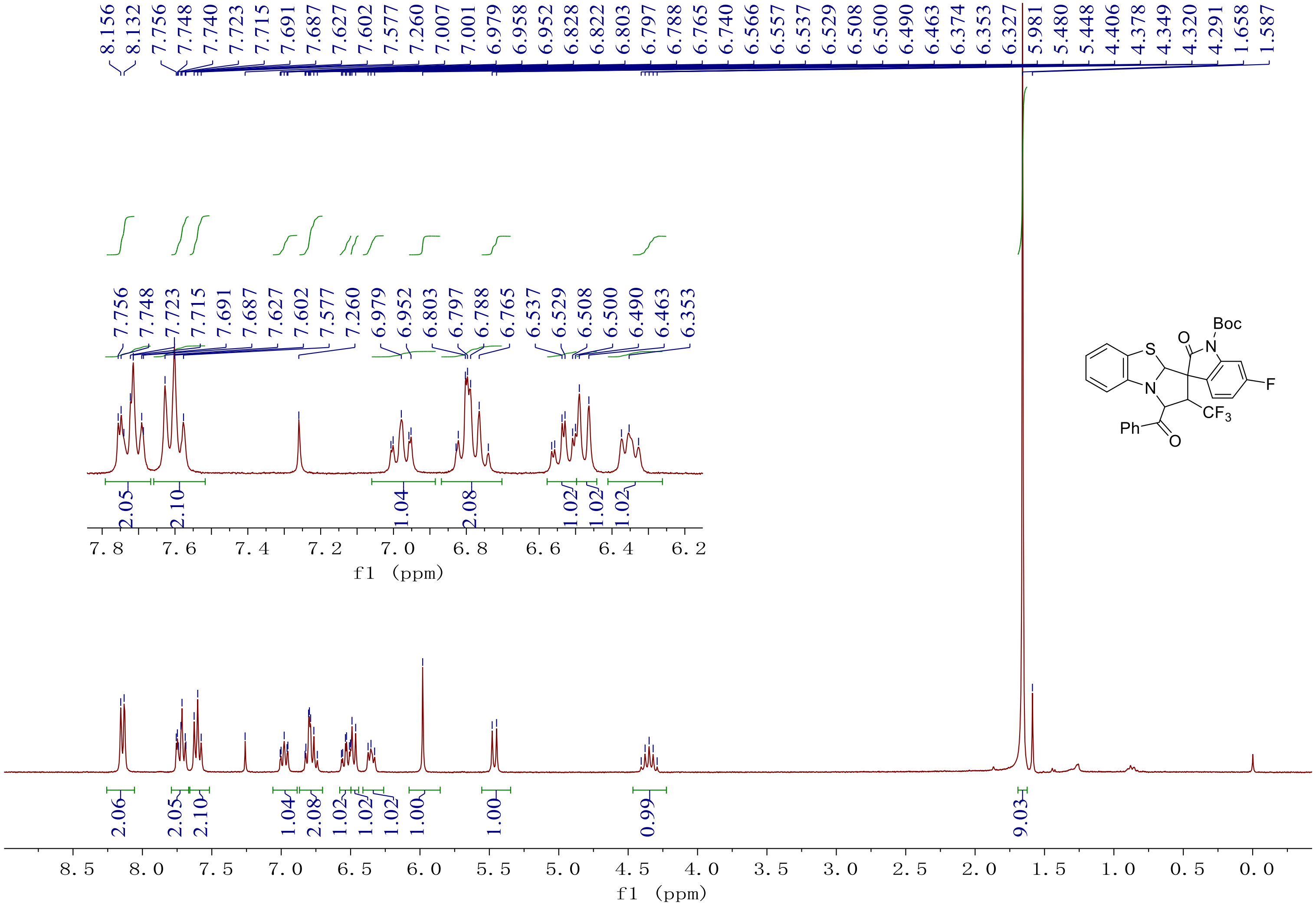

^{13}C NMR (75 MHz, $CDCl_3$) of **5ab**

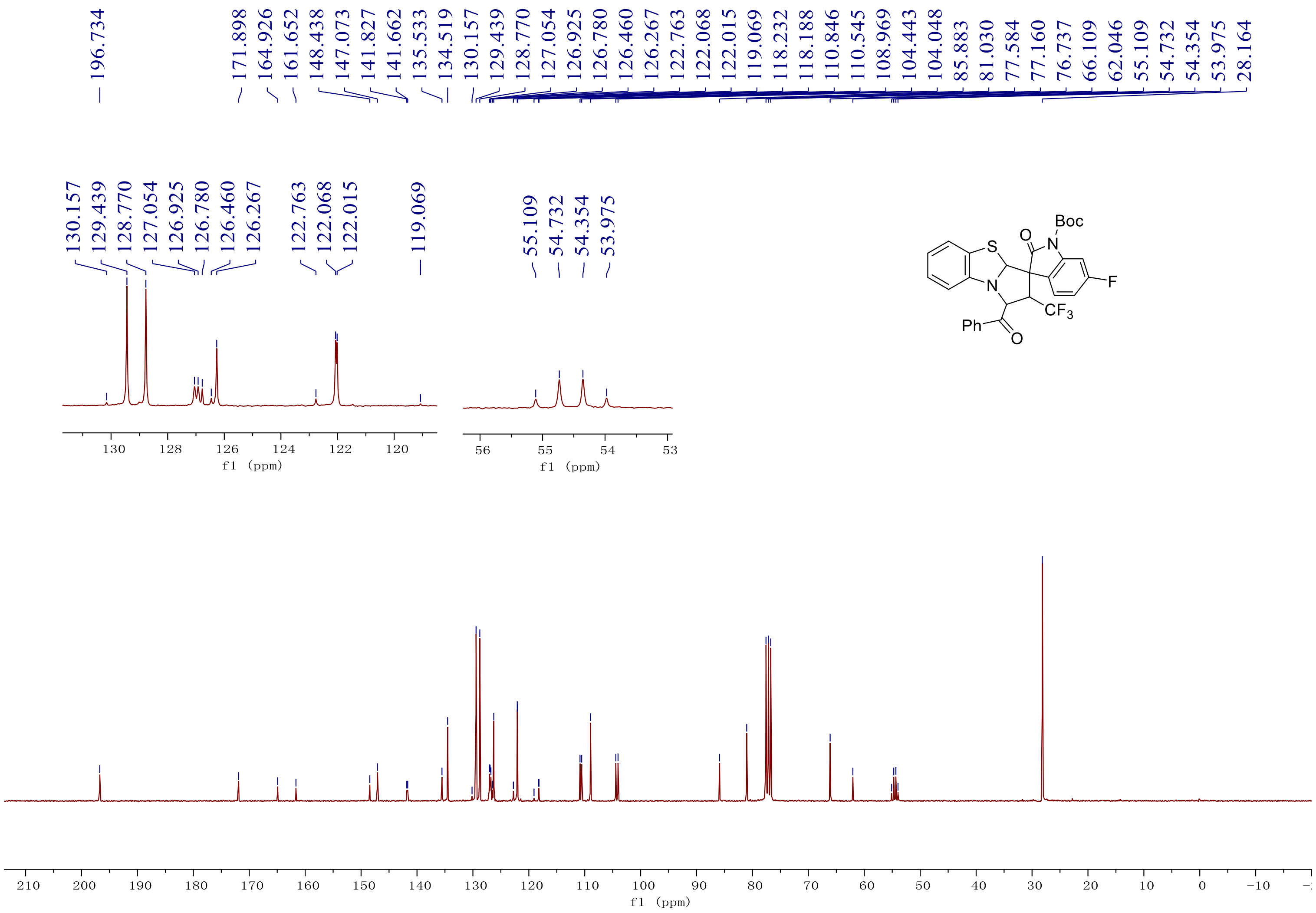

^{1}H NMR (300 MHz, $CDCl_3$) of **5ac**

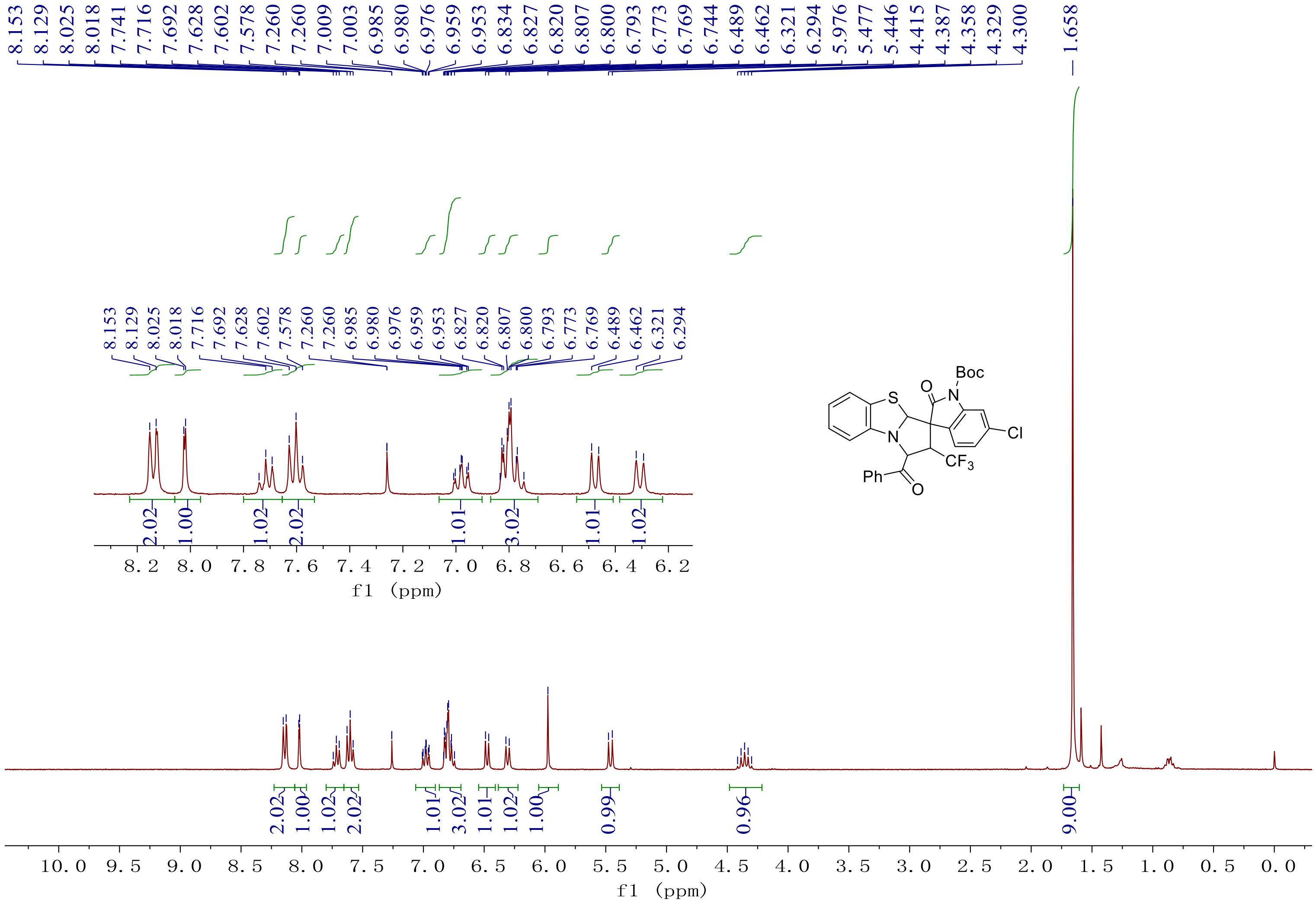

^{13}C NMR (75 MHz, $CDCl_3$) of **5ac**

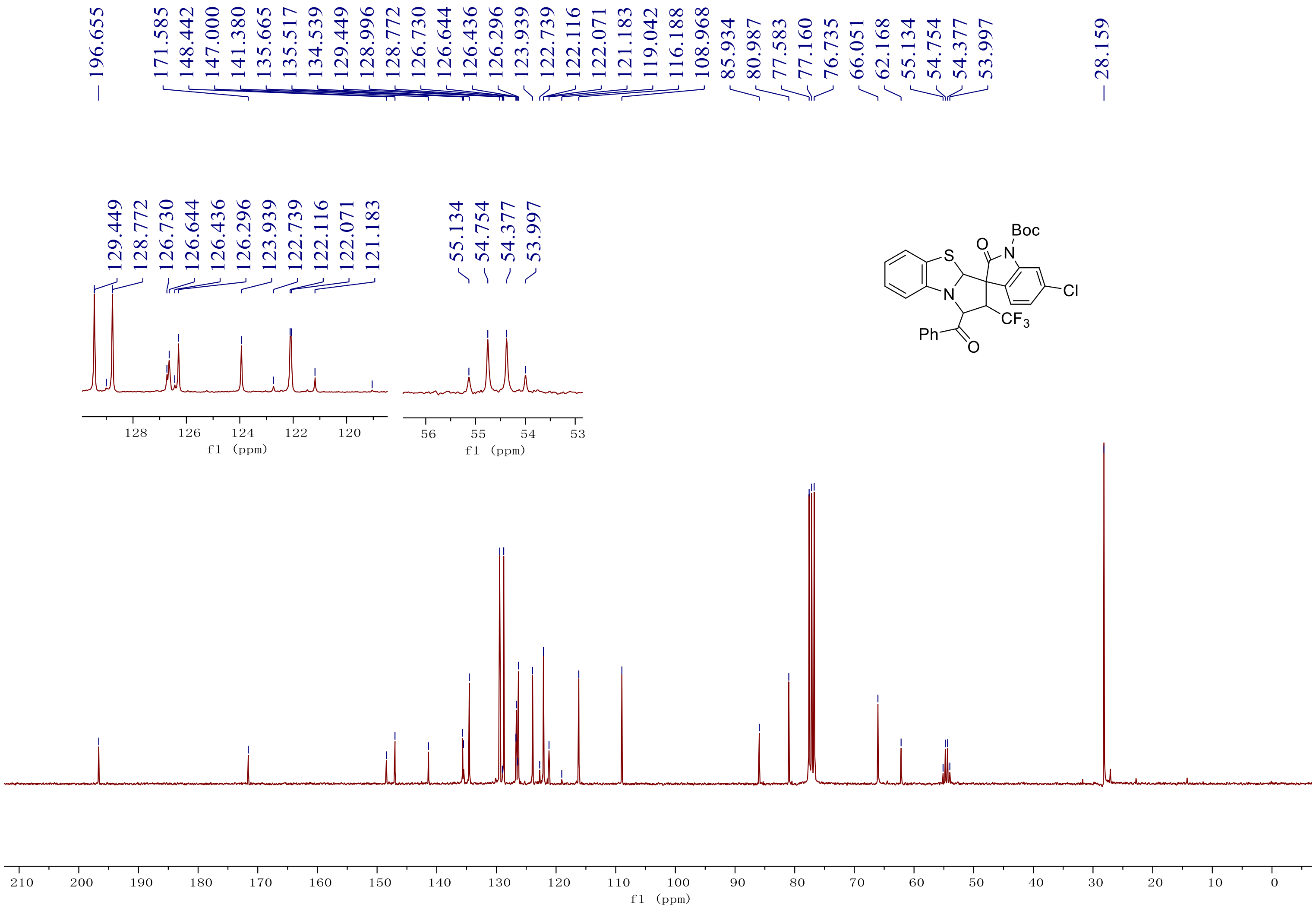

^{1}H NMR (300 MHz, $CDCl_3$) of **5ad**

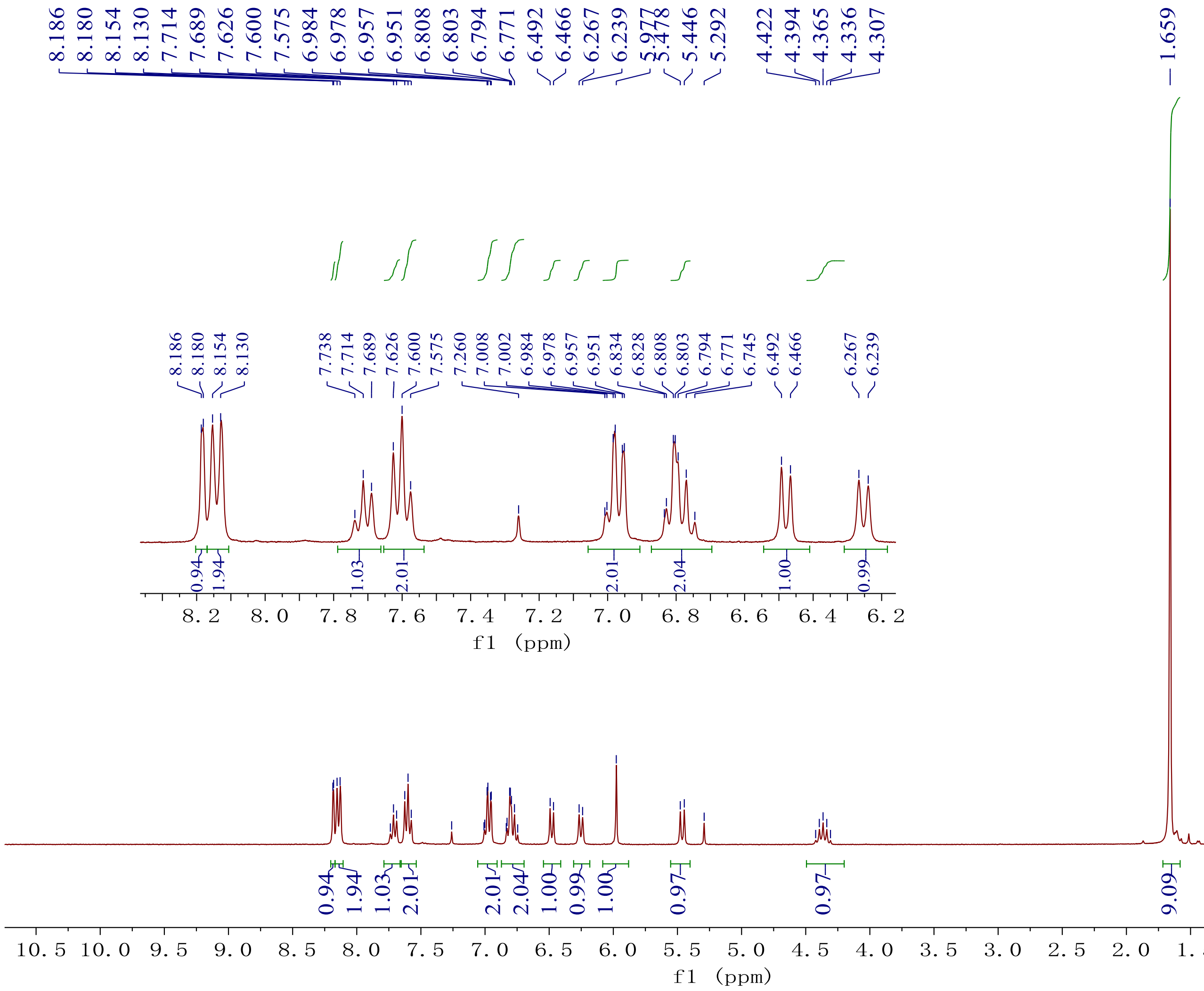

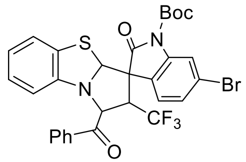

^{13}C NMR (75 MHz, $CDCl_3$) of **5ad**

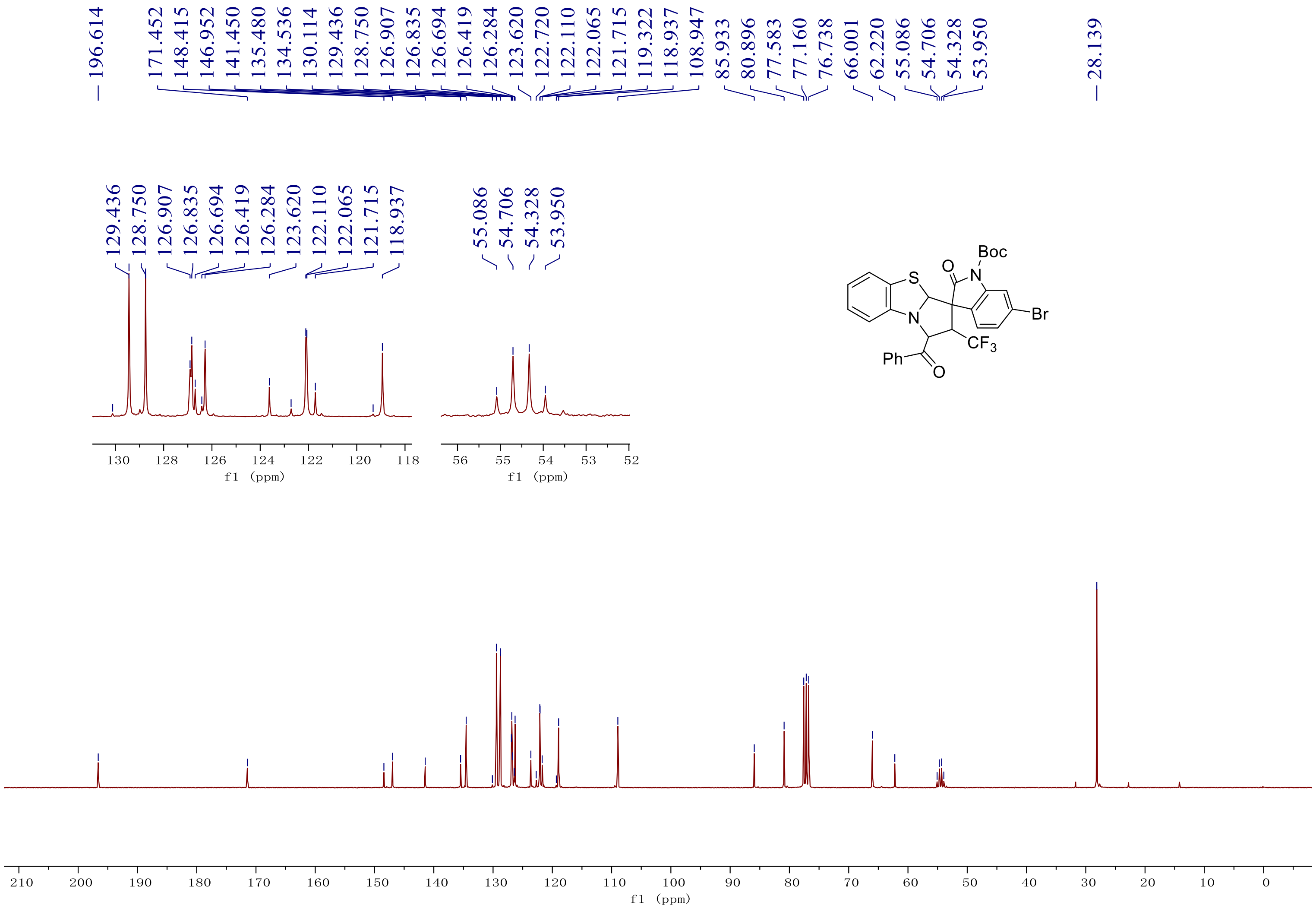

^{1}H NMR (300 MHz, $CDCl_3$) of **5ae**

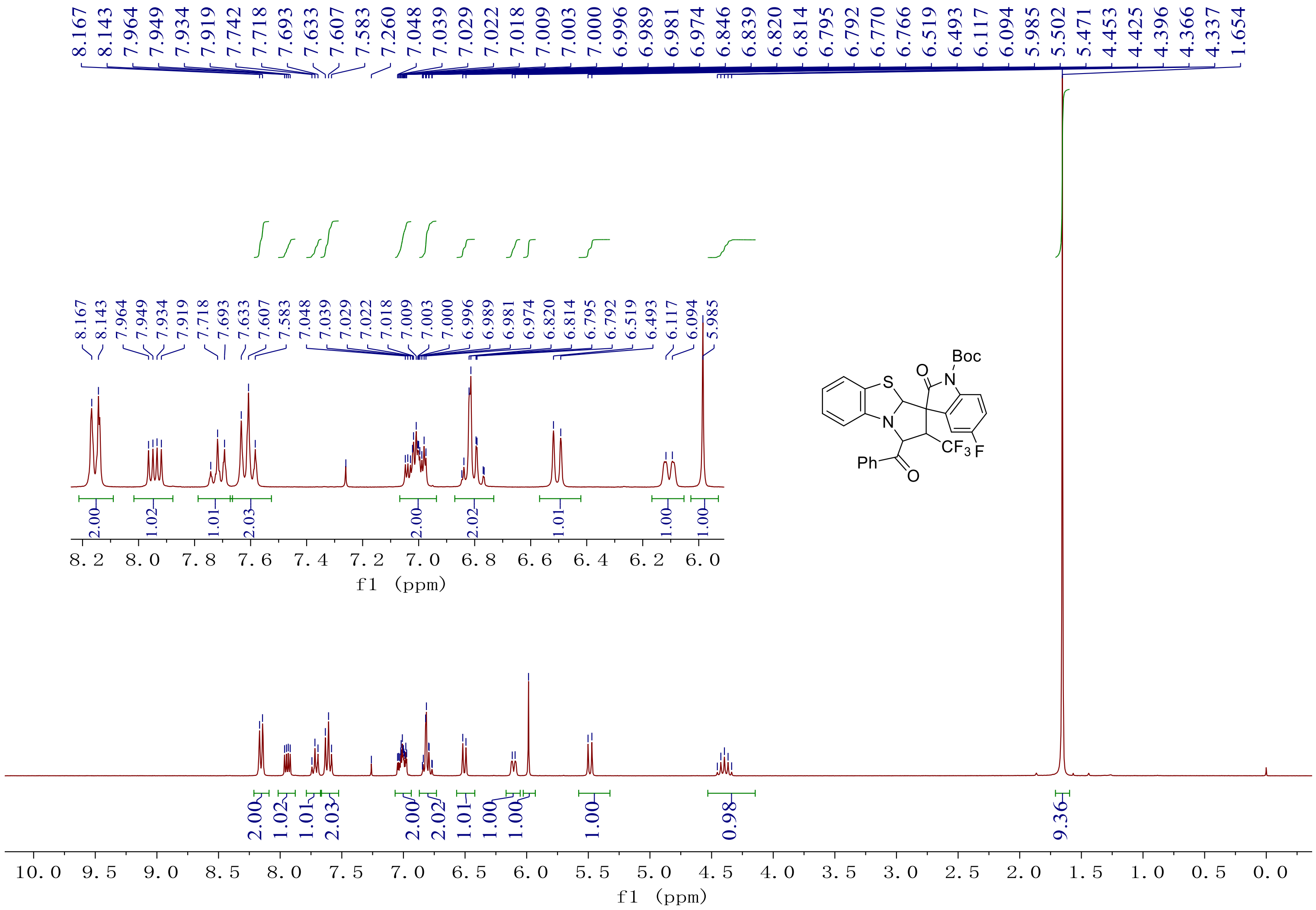

^{13}C NMR (75 MHz, $CDCl_3$) of **5ae**

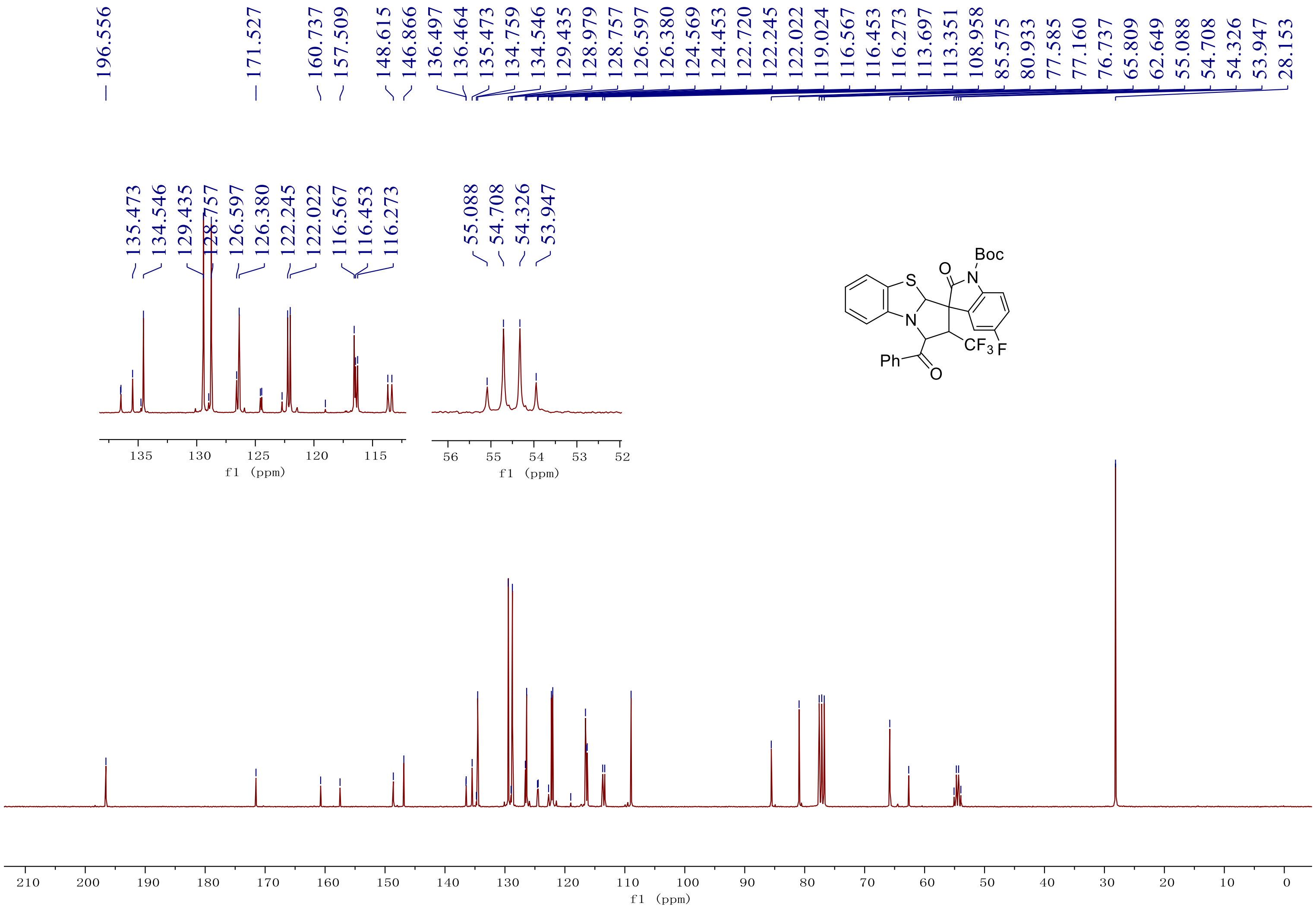

^{1}H NMR (400 MHz, $CDCl_3$) of **5af**

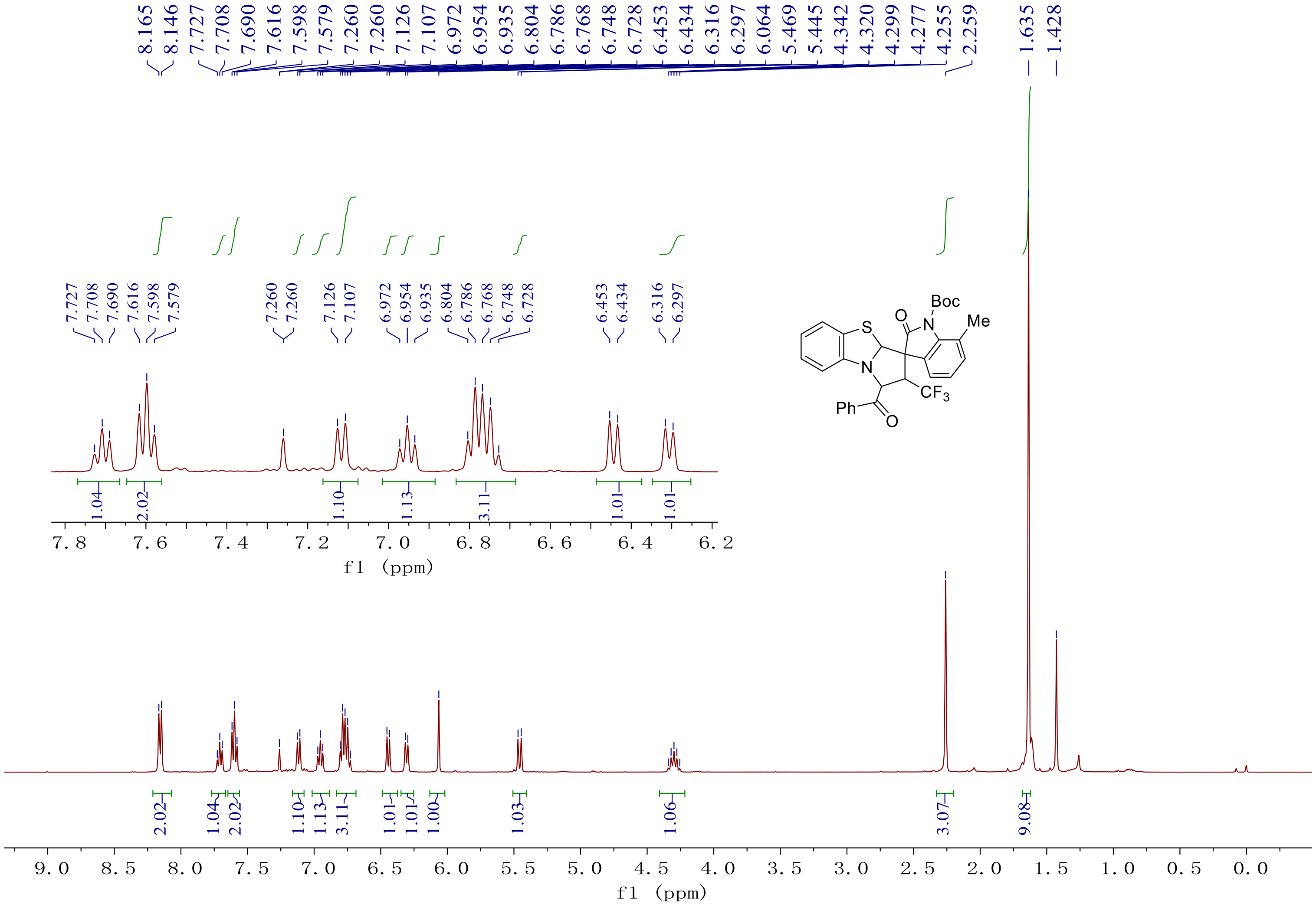

^{13}C NMR (101 MHz, CDCl$_3$) of **5af**

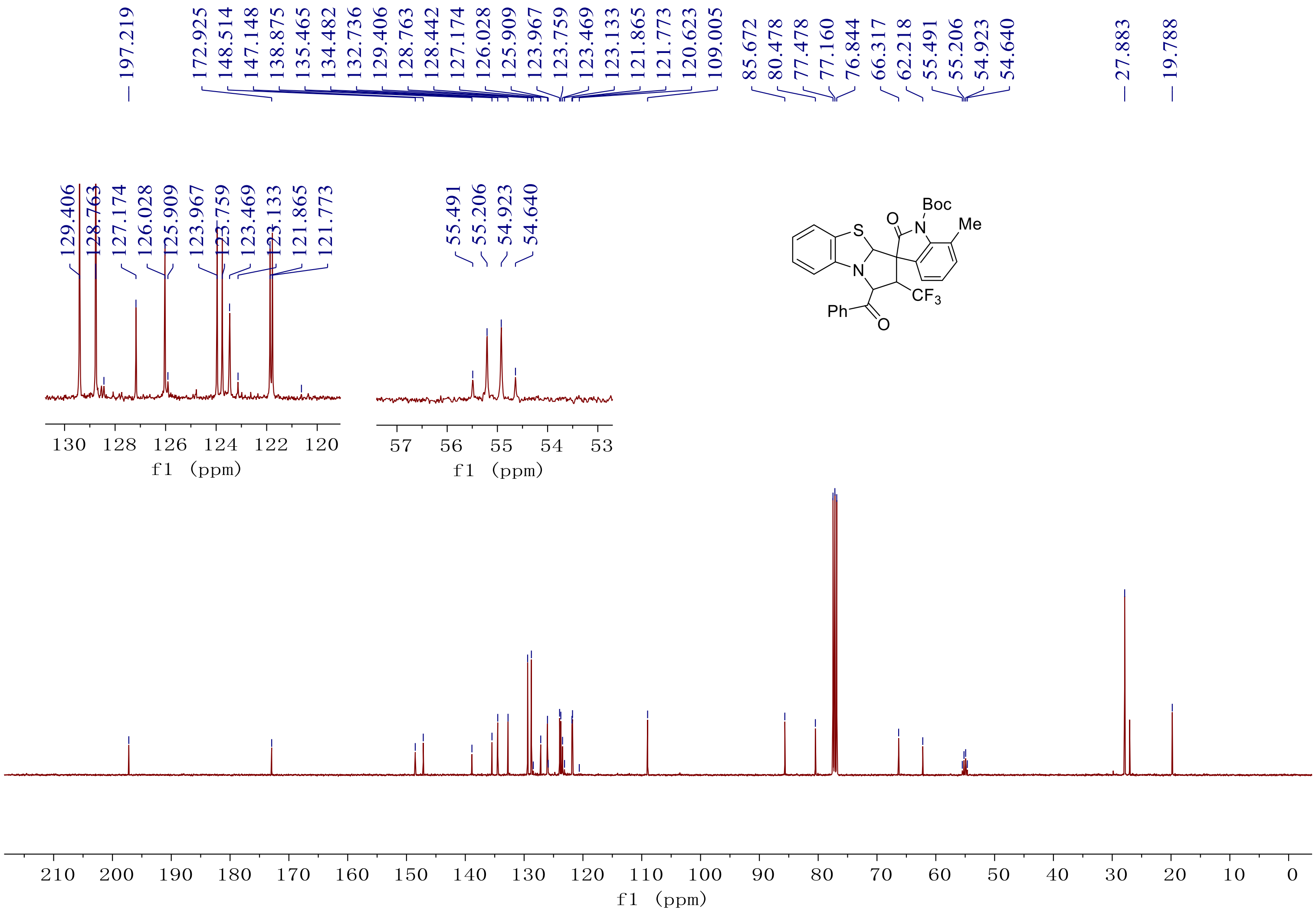

^{1}H NMR (300 MHz, $CDCl_3$) of **5ag**

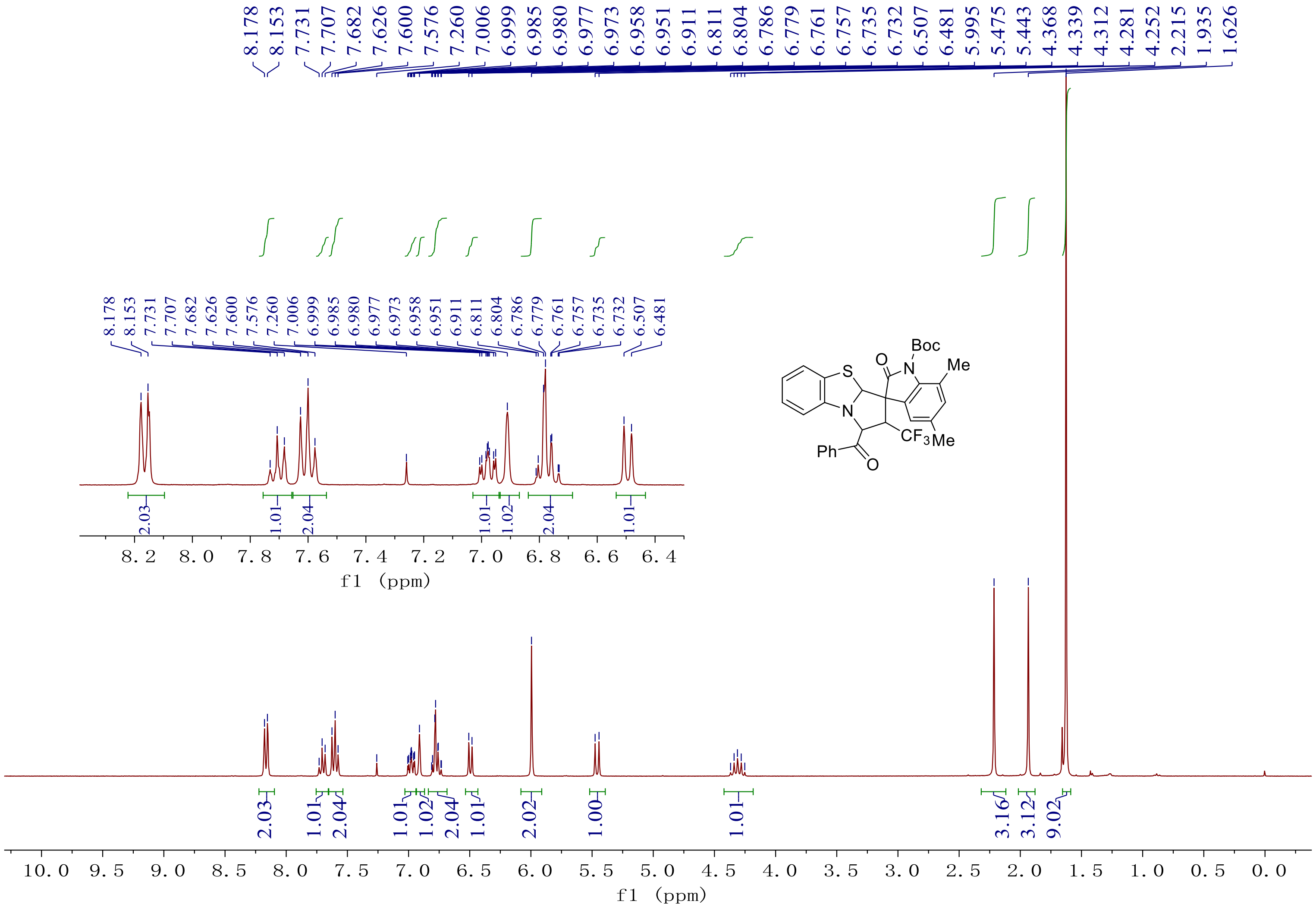

^{13}C NMR (75 MHz, $CDCl_3$) of **5ag**

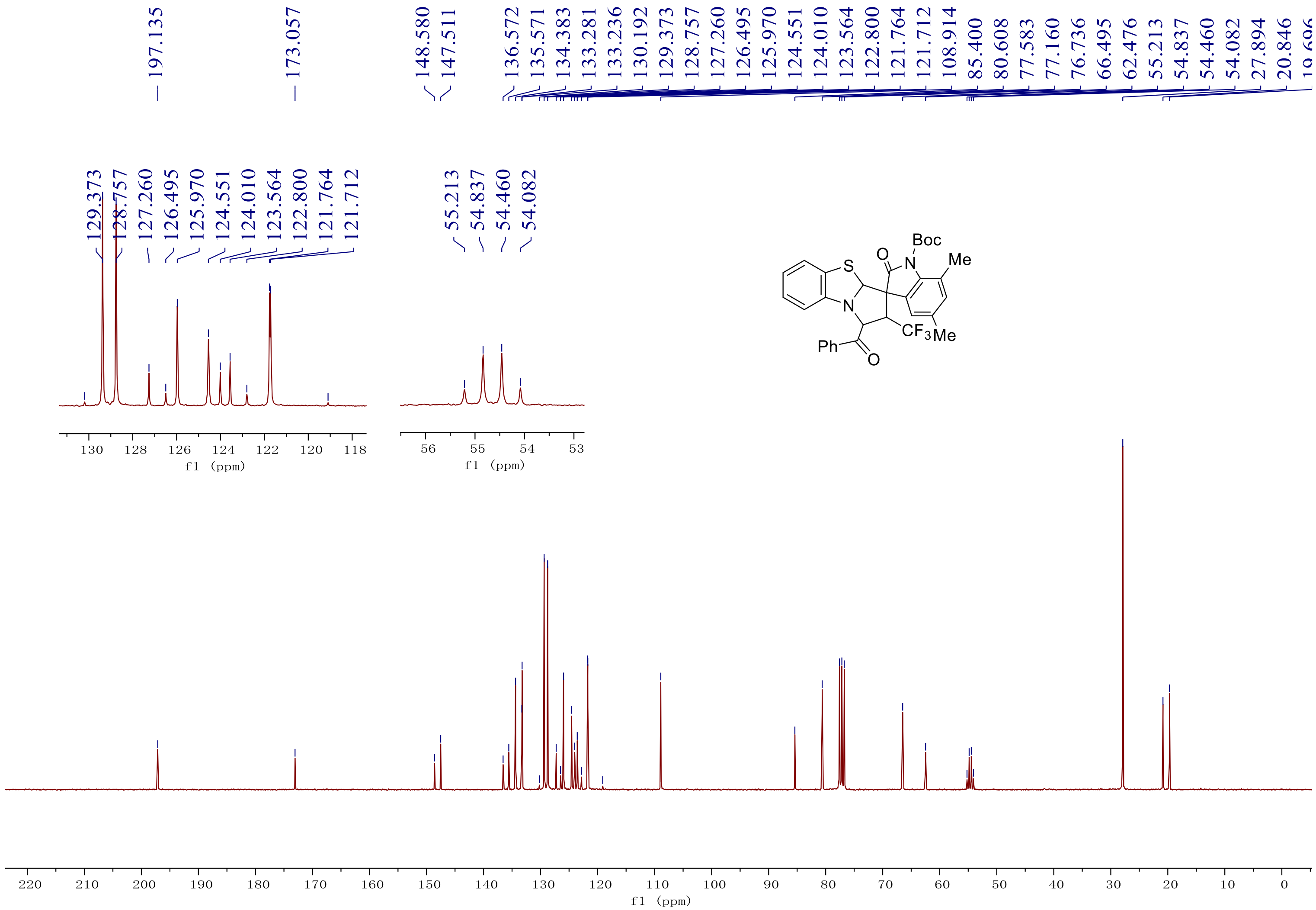

^{1}H NMR (300 MHz, $CDCl_3$) of **5ah**

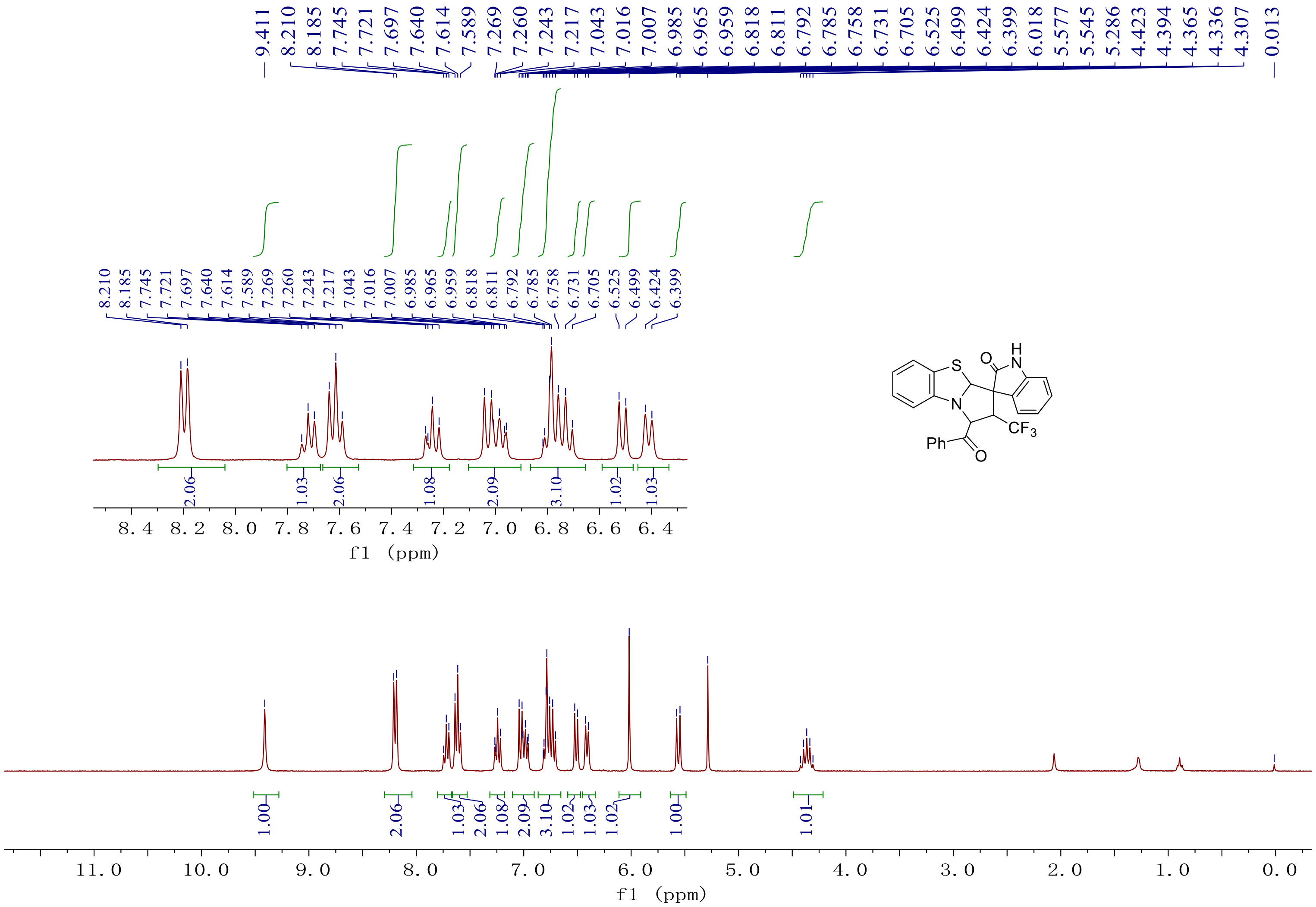

^{13}C NMR (75 MHz, $CDCl_3$) of **5ah**

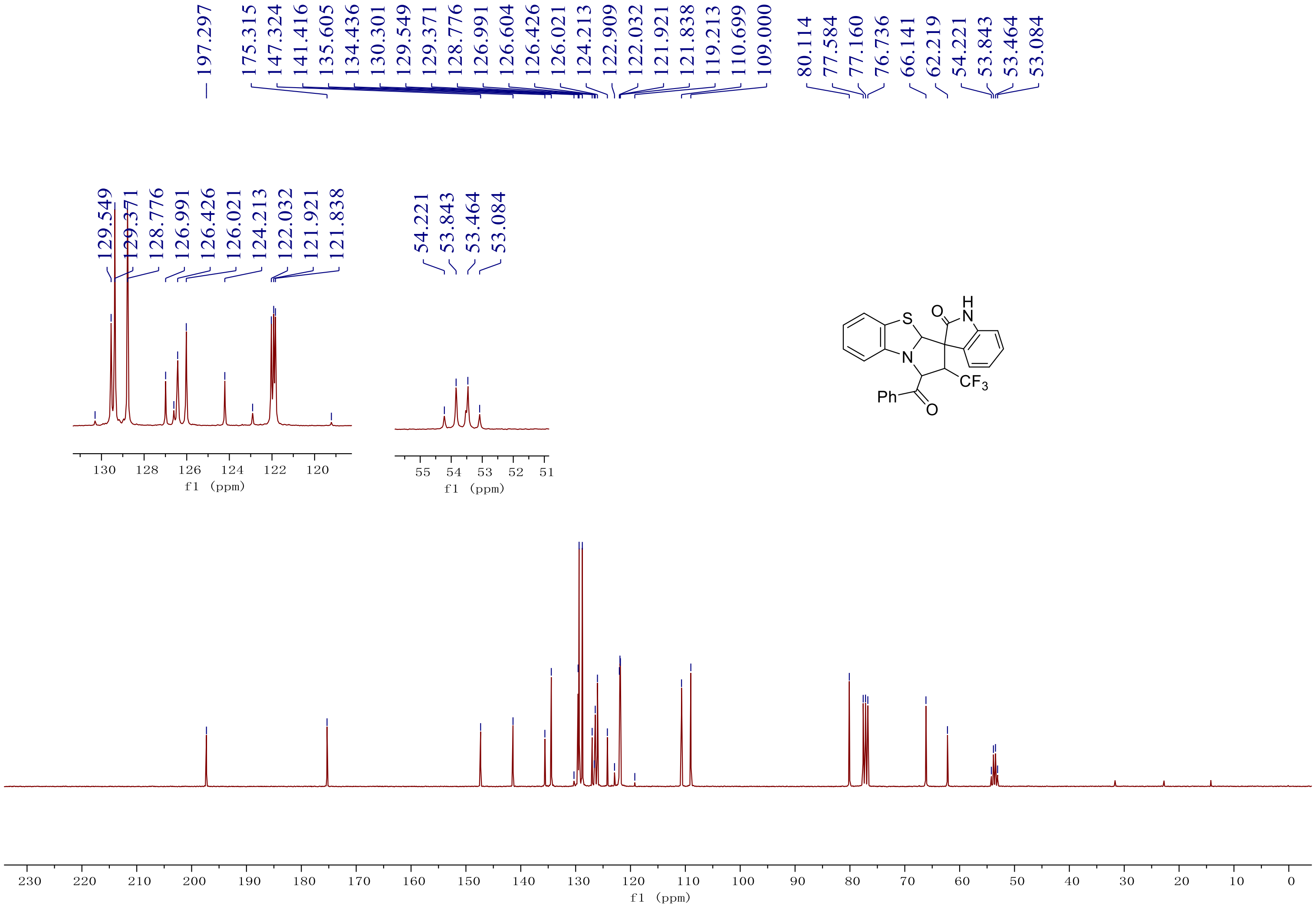

^{1}H NMR (300 MHz, $CDCl_3$) of **5ai**

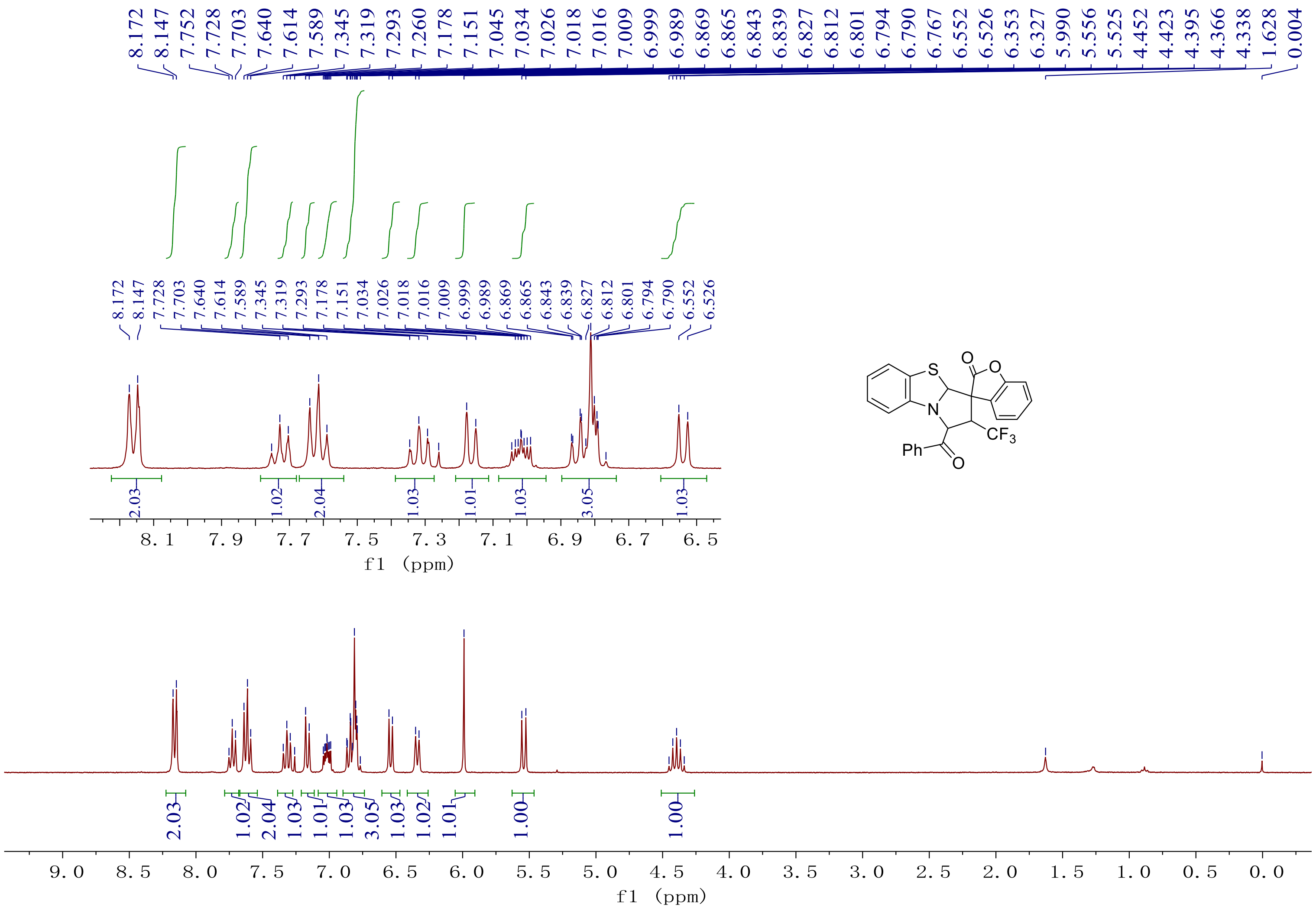

^{13}C NMR (75 MHz, CDCl$_3$) of **5ai**

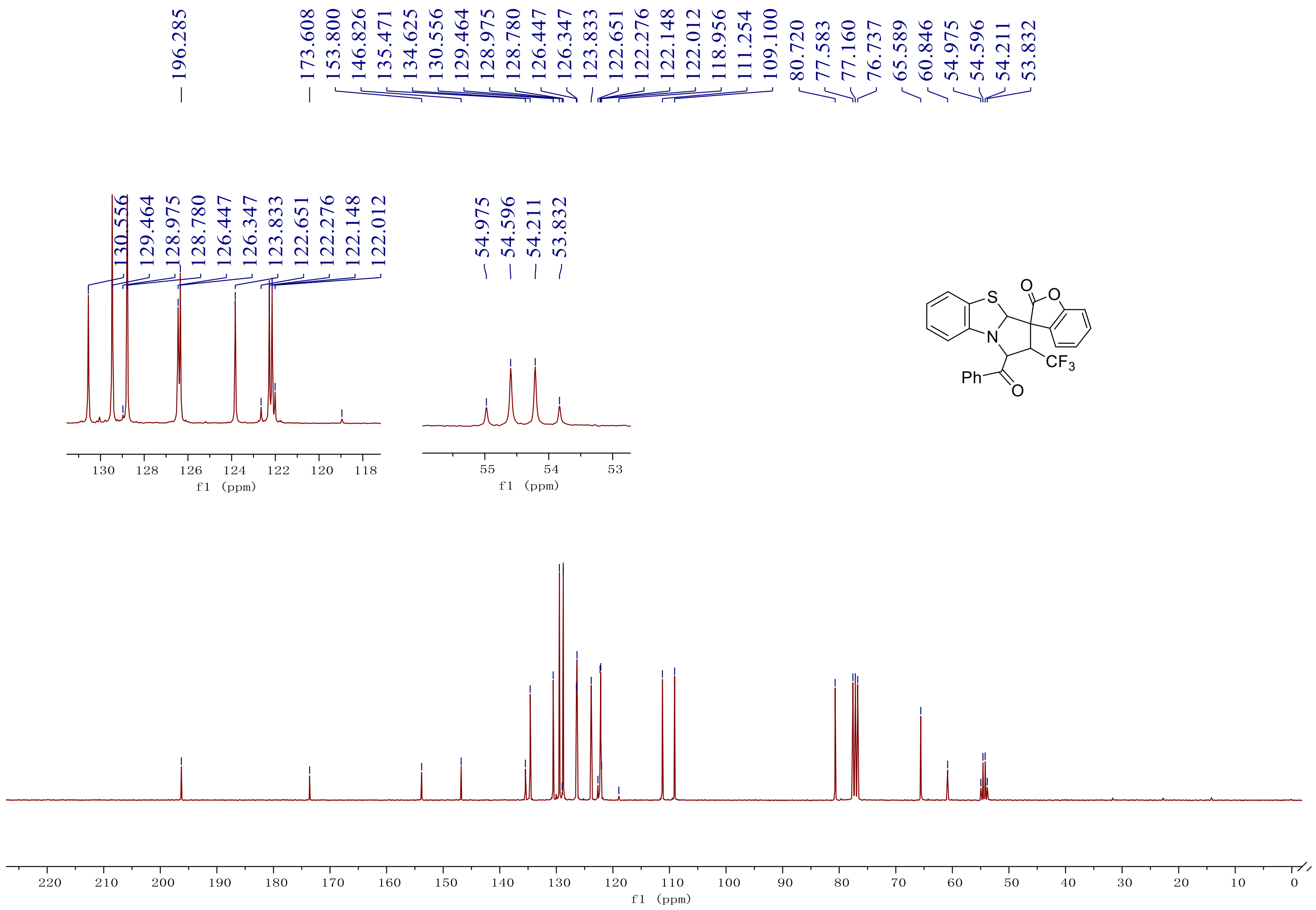

^{1}H NMR (400 MHz, $CDCl_3$) of **7aa**

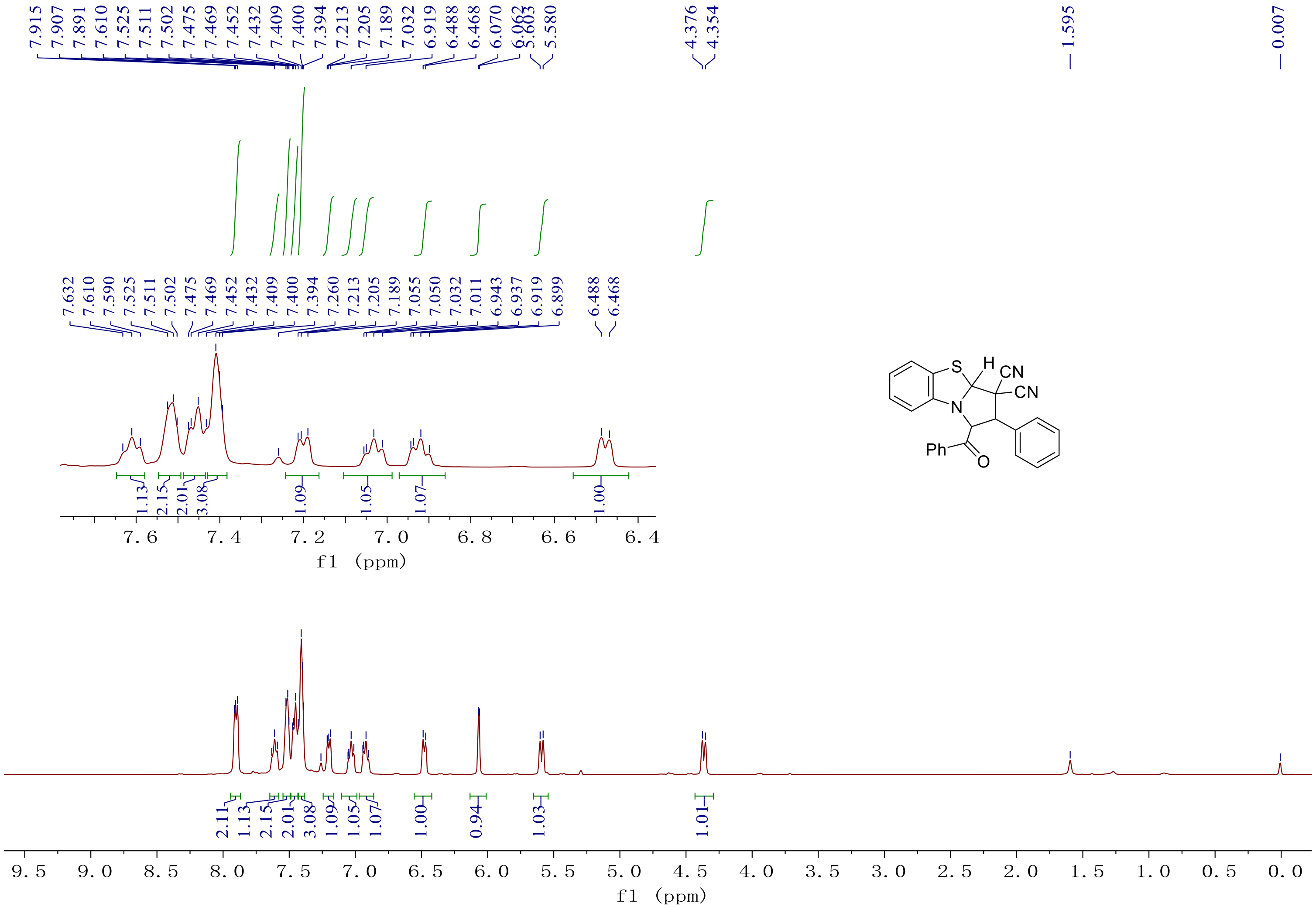

^{13}C NMR (101 MHz, $CDCl_3$) of **7aa**

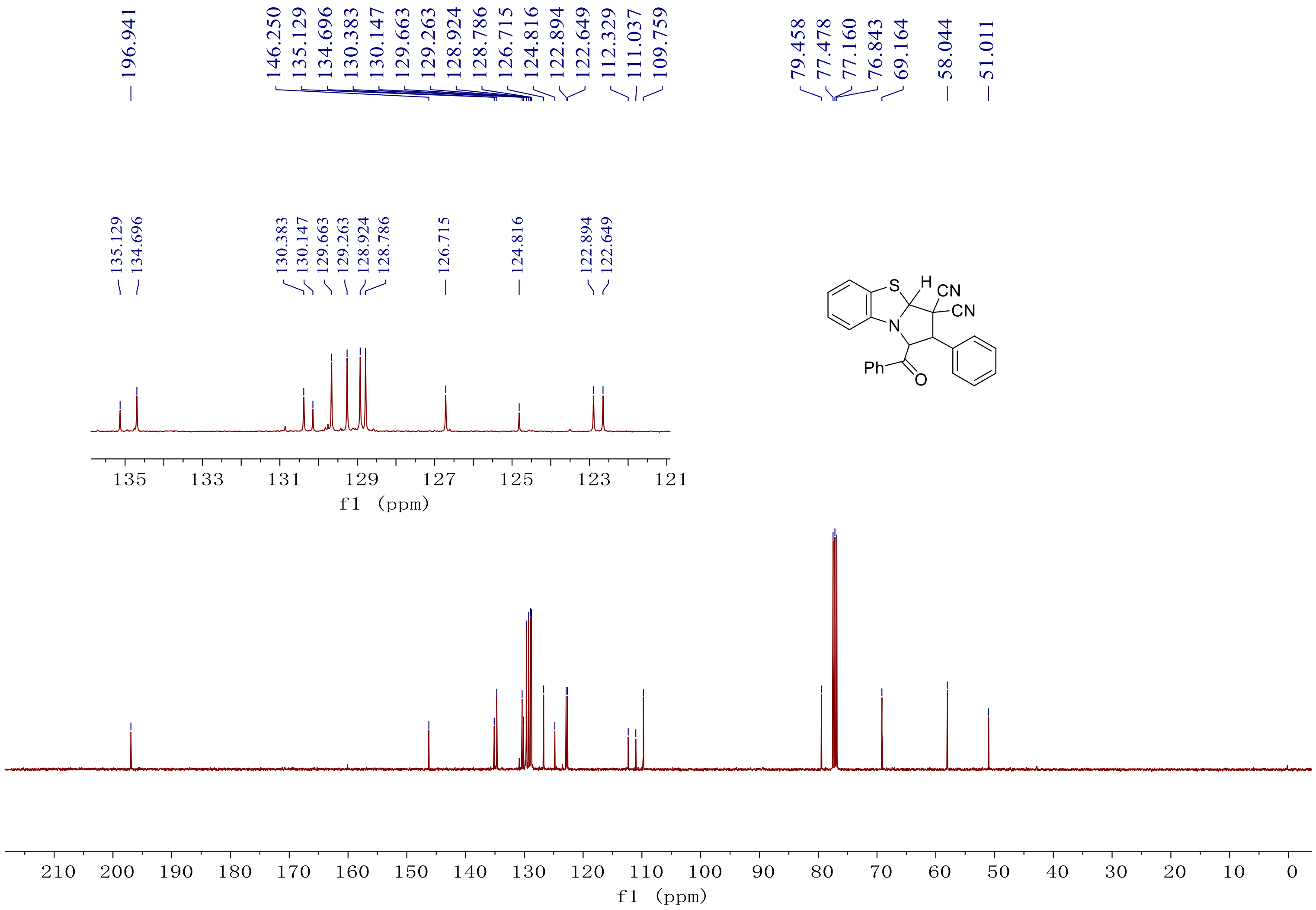

^{1}H NMR (400 MHz, $CDCl_3$) of **7ab**

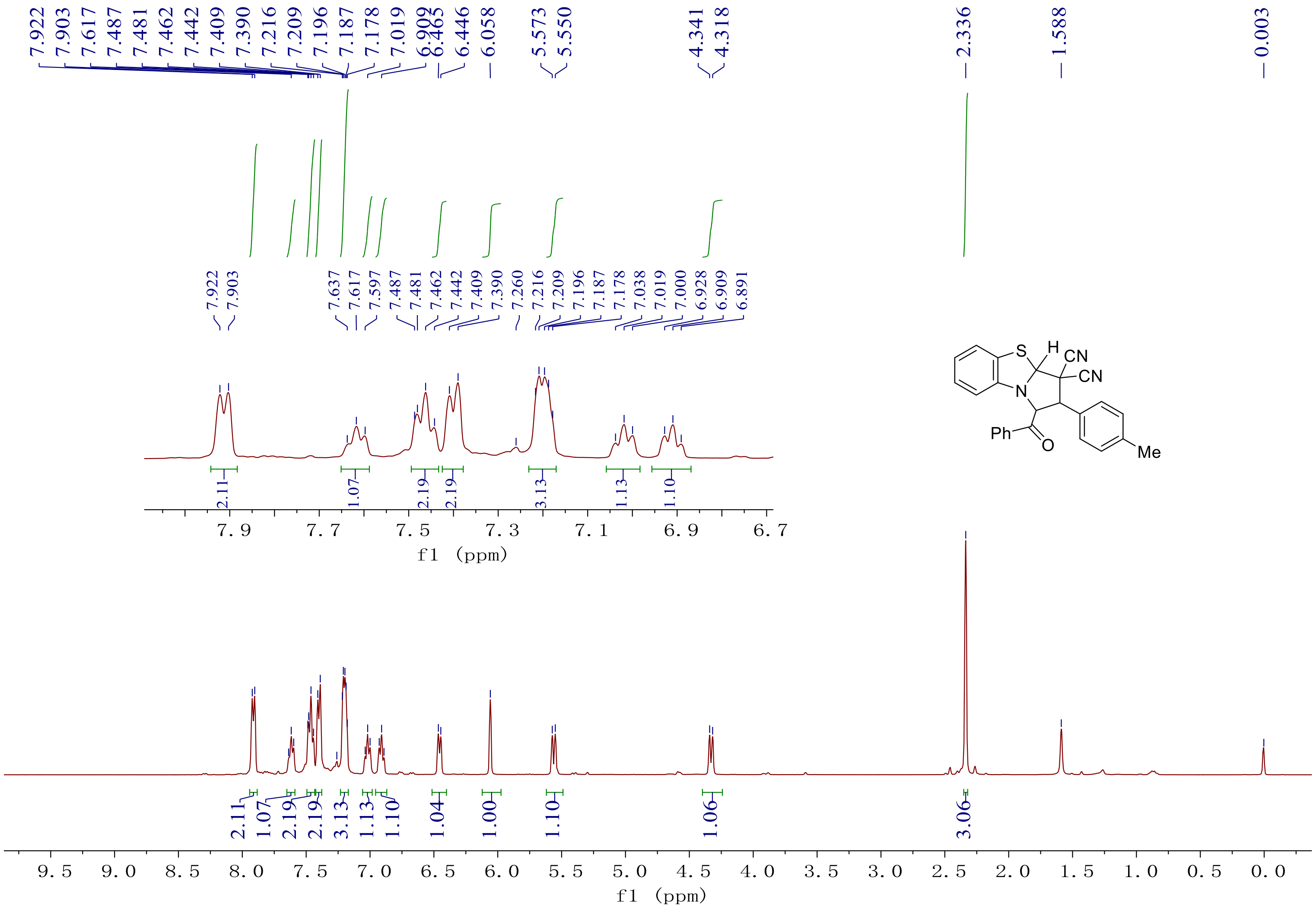

^{13}C NMR (101 MHz, $CDCl_3$) of **7ab**

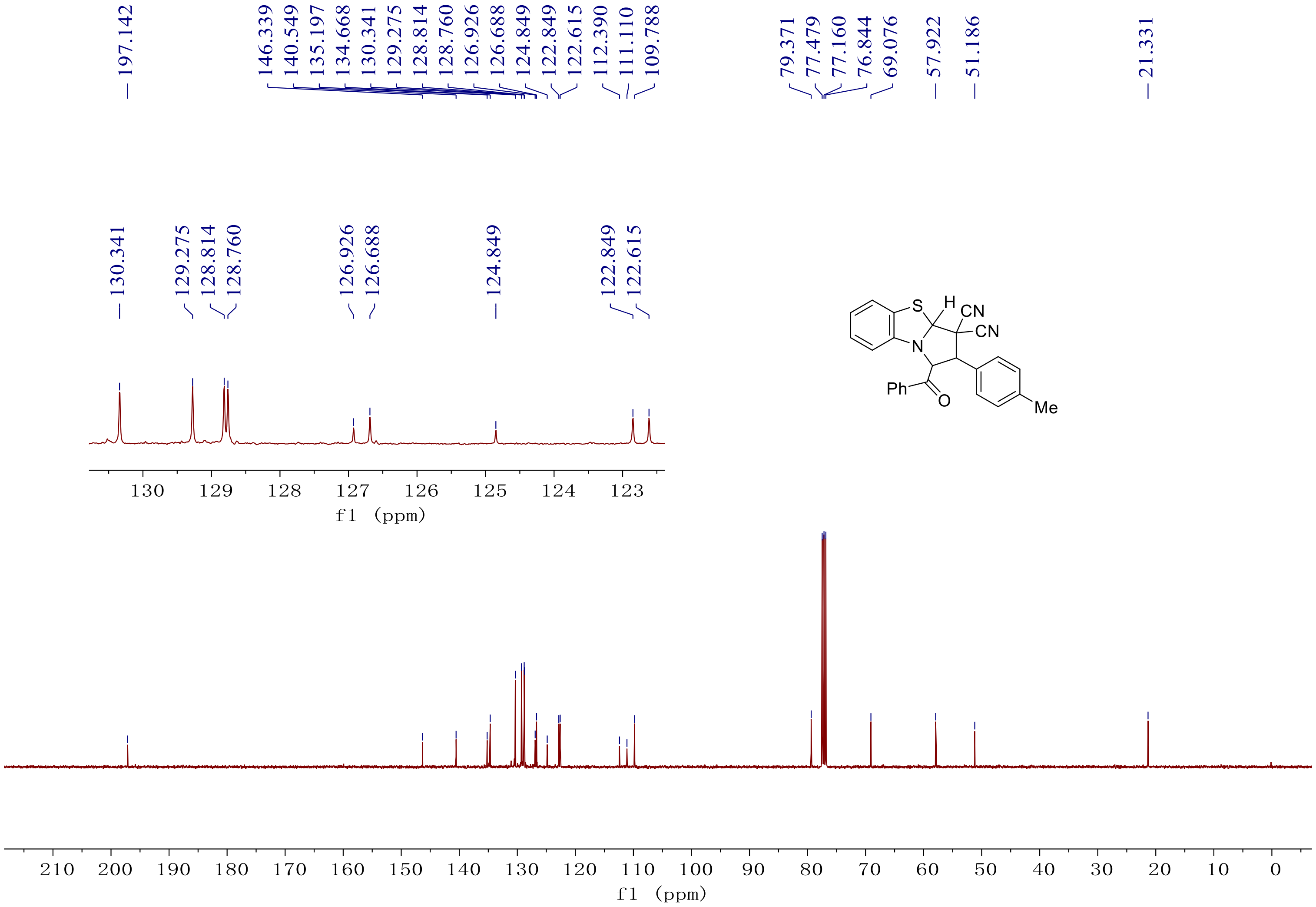

^{1}H NMR (400 MHz, $CDCl_3$) of **7ac**

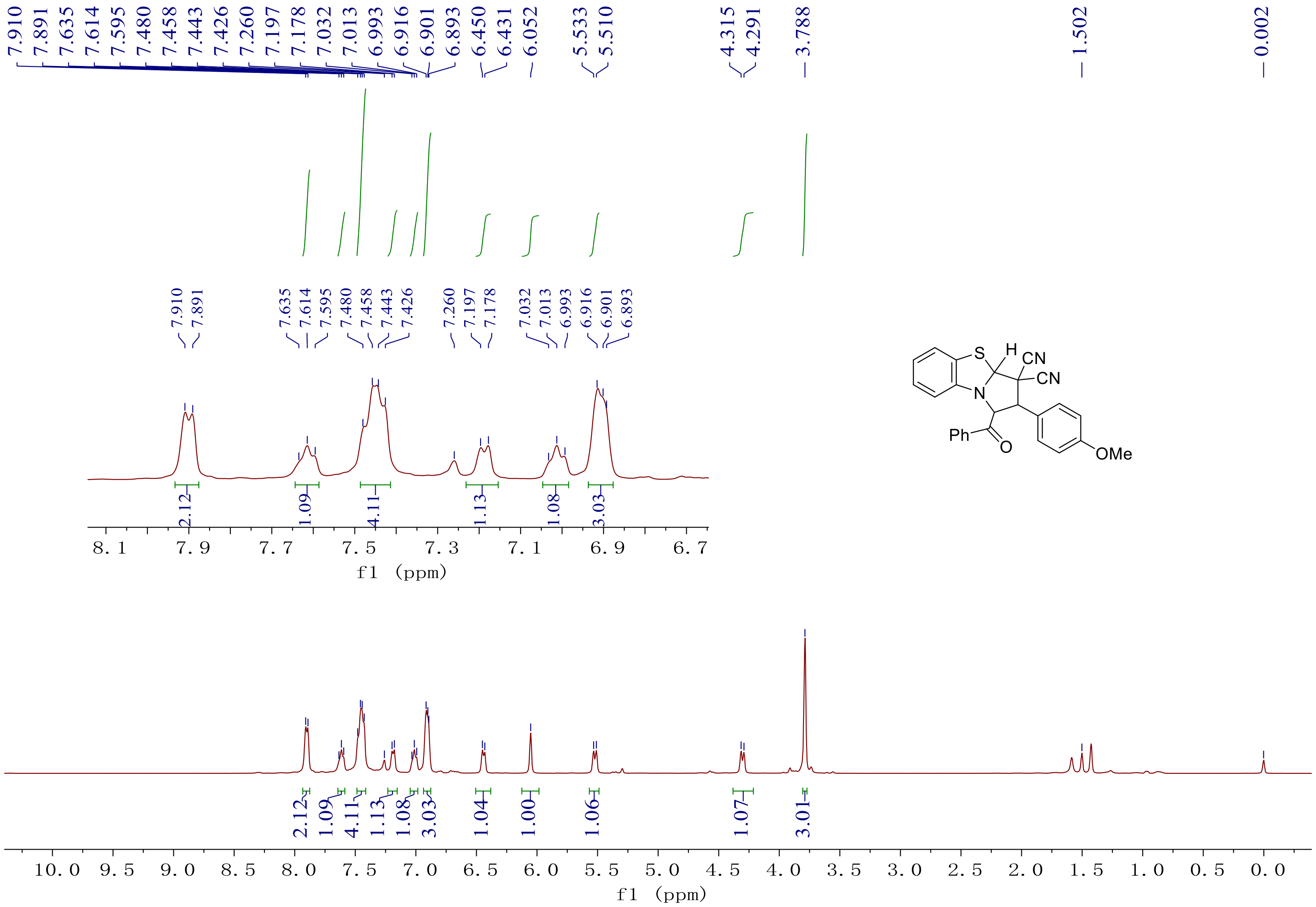

^{13}C NMR (101 MHz, CDCl$_3$) of **7ac**

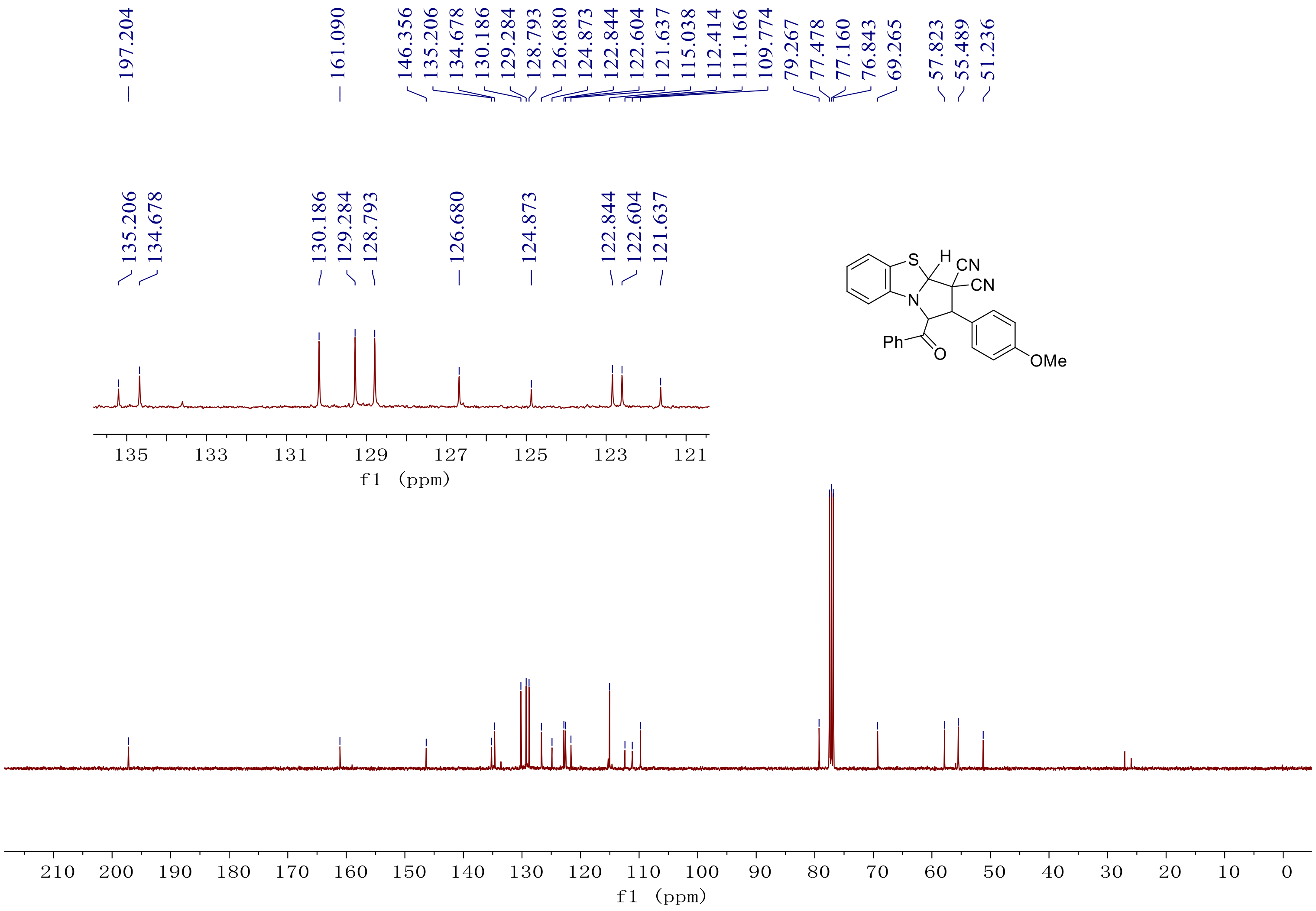

^{1}H NMR (400 MHz, $CDCl_3$) of **7ad**

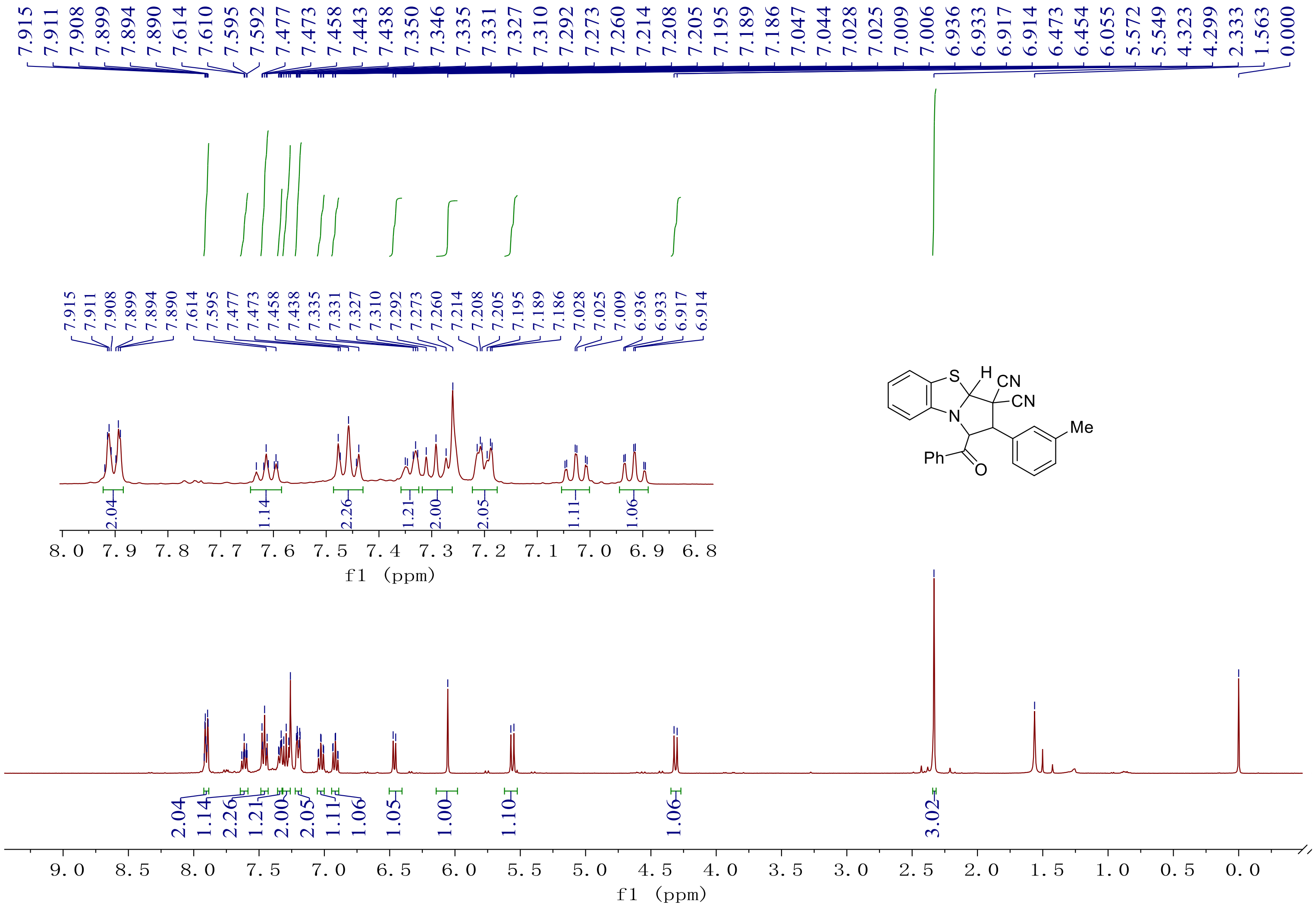

^{13}C NMR (101 MHz, $CDCl_3$) of **7ad**

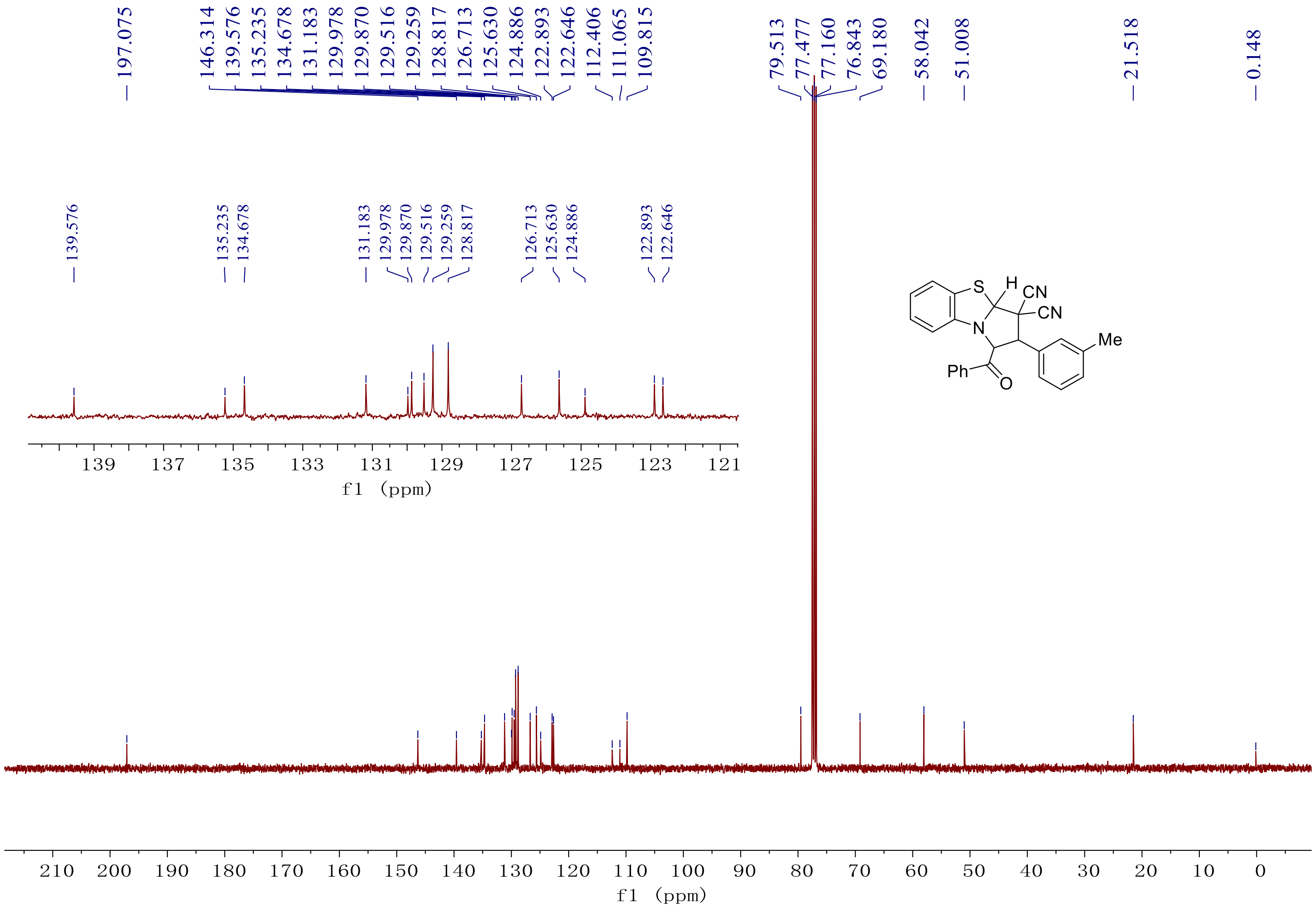

^{1}H NMR (400 MHz, $CDCl_3$) of **7ae**

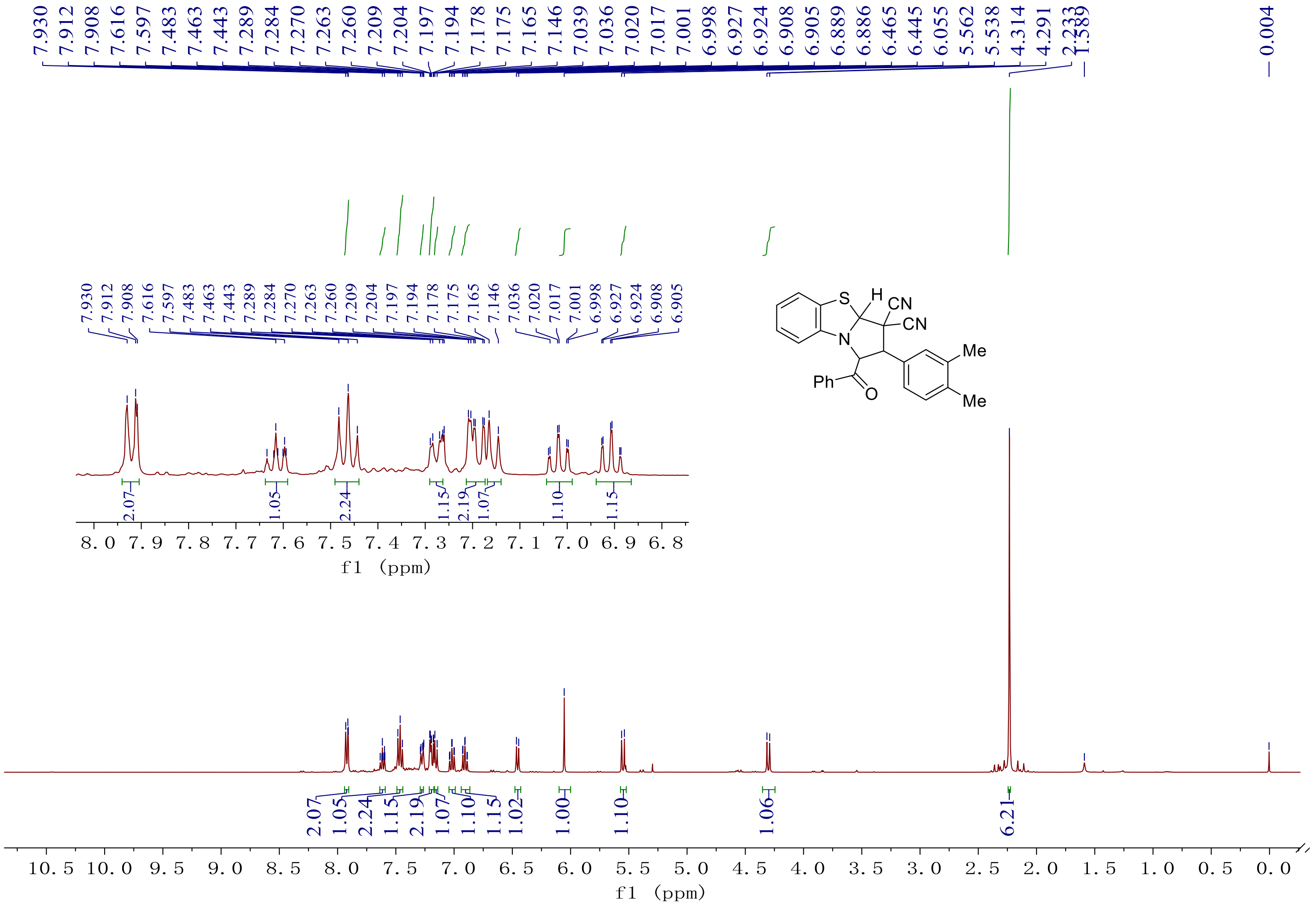

^{13}C NMR (101 MHz, CDCl$_3$) of **7ae**

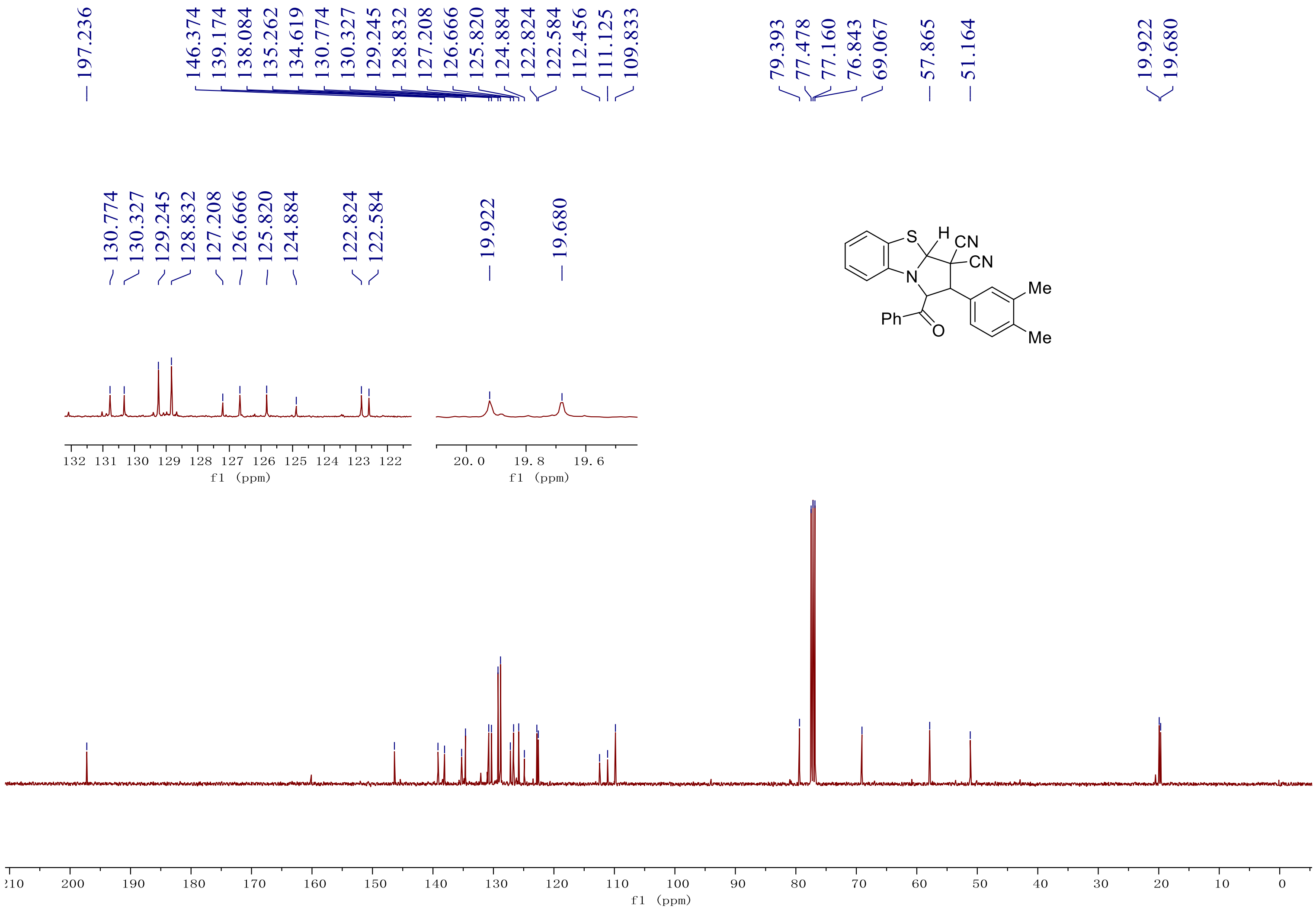

^{1}H NMR (400 MHz, $CDCl_3$) of **7af**

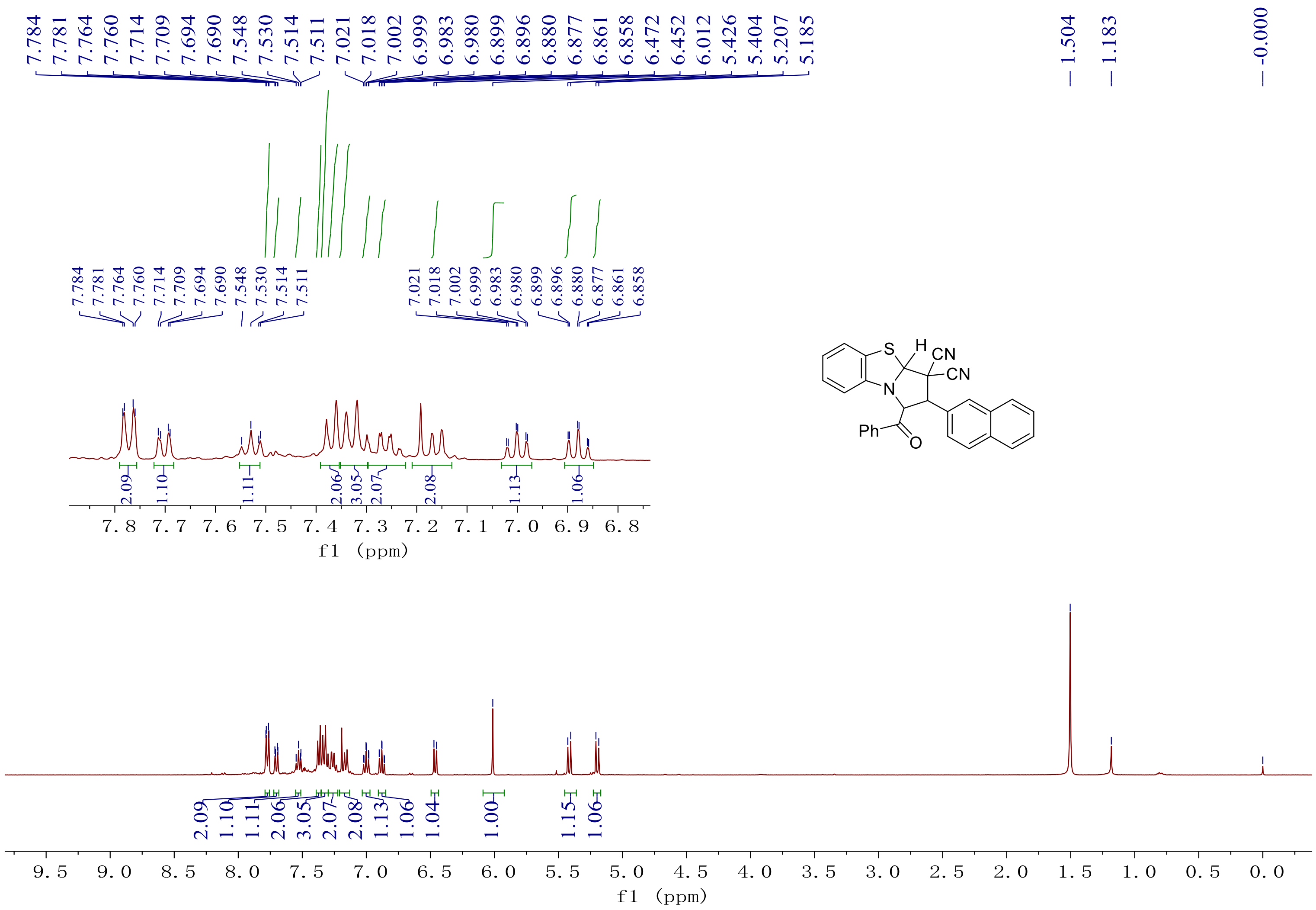

^{13}C NMR (101 MHz, CDCl$_3$) of **7af**

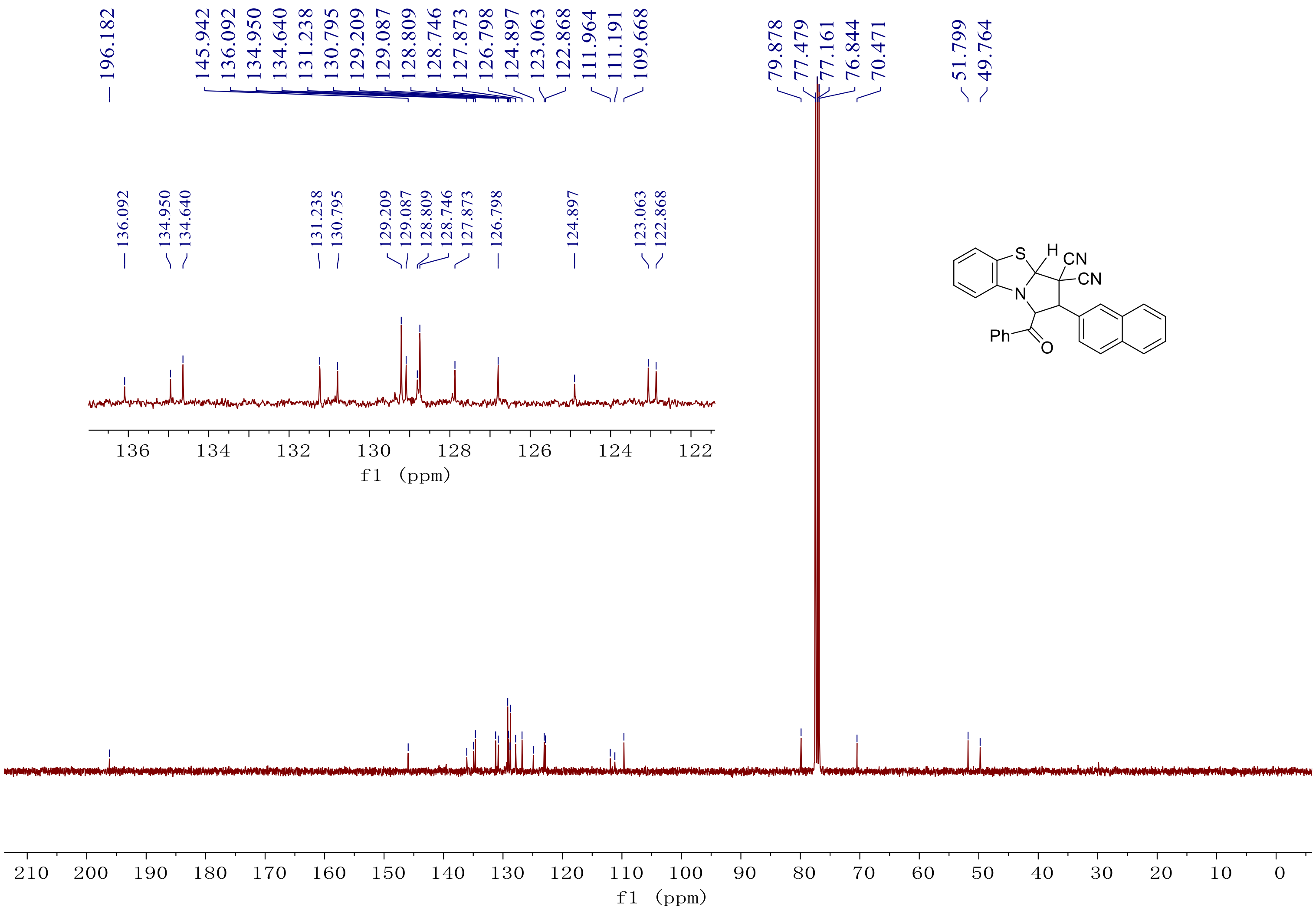

Supplement: Supplementary file 1 [file molecules-28-04410-s001.zip › Supplementary File/supporting information.pdf]
